# Mild and Effective Method for the Nickel-Catalyzed Arylation of Glycosyl Thiols in Aqueous Surfactant Solution

**DOI:** 10.1021/acs.joc.4c02233

**Published:** 2024-11-12

**Authors:** Zoe Beato, Sally Howard Ihle, Xiangming Zhu

**Affiliations:** † Centre for Synthesis and Chemical Biology, UCD School of Chemistry, University College Dublin, Belfield Dublin 4, Ireland; ‡ BiOrbic, Bioeconomy SFI Research Centre, University College Dublin, Belfield Dublin 4, Ireland

## Abstract

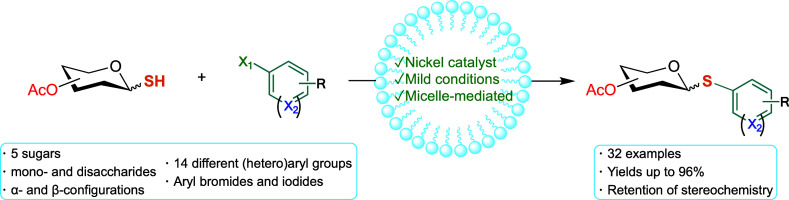

Aryl thioglycosides have broad applicability as both glycosyl donors and glycomimetic compounds. Their synthesis via the cross-coupling of glycosyl thiols with aryl halides has become a popular method for their construction because it allows better selectivity for anomeric configuration as well as a wider functional group tolerance compared to traditional methods. Herein, we report a nickel-catalyzed method for the synthesis of aryl thioglycosides which utilizes an aqueous micellar environment as the reaction medium. This alternative method allows for mild conditions while circumventing expensive palladium, leading to the successful synthesis of over 30 aryl thioglycosides, including challenging 1,2-*cis* thioglycoside products.

## 1 Introduction

Thioglycosides are carbohydrate compounds where oxygen in a glycosidic linkage is replaced by sulfur. These molecules are a rare occurrence in nature (though some examples exist),^1^ but they have become especially useful in synthetic carbohydrate chemistry. One of their main applications is as glycosyl donors: in the presence of thiophilic promoters, the –SR group becomes a leaving group, facilitating nucleophilic attack of a glycosyl acceptor. While there are a variety of glycosyl donors in use, thioglycosides are especially popular for their excellent stability.^2^ They withstand a range of conditions used in protecting group manipulations, as well as activation of other more labile donors. Additionally, varying the protecting groups,^3^ aglycon,^4^ anomeric configuration,^5^ or activation conditions^6^ can allow chemoselective activation between thioglycoside donors. Thus, they are often utilized in one-pot procedures, acting sequentially as acceptors then as donors in the next glycosylation. The aglycon unit of thioglycoside donors is often a simple alkyl or aryl group such as ethyl, phenyl, or tolyl, but there are already reports in literature of the effect of varying the aglycon unit on the reactivity of the donor for both alkyl and substituted aryl groups.^7^ Reactivity differences based on aglycon alone are not always sufficient to effect selective activation. Despite this, there are cases where differently substituted aryl thioglycosides are, in fact, useful in chemoselective glycosylation reactions.

Beyond their use as glycosyl donors, *S*-linked glycosides have also gained popularity as glycomimetics. They often mimic the activity of *O*-glycosides, but the bioisosteric *S*-glycosides are more resistant to enzymatic cleavage. The stability stems from the lower proton affinity of sulfur versus oxygen and the lesser degree of polarization of the C–S compared to the C–O bonds.^8^*S*-Glycosides are also useful as irreversible glycosidase inhibitors^9^ and in the elucidation of enzymatic structures.^10^ Some examples of biologically active thioglycosides include antibiotics such as clindamycin and lincomycin, sotagliflozin^11^ and other SGLT1/2 inhibitors,^12^ immunostimulants,^13^ analgesics,^14^ and antiviral agents.^15^

Due to their widespread usage, many methods have been devised for the synthesis of thioglycosides. The most common method involves the reaction of a per-acetylated sugar with a Lewis acid (often BF_3_ etherate) and an alkyl or aryl thiol to generate an anomeric thioglycoside. Despite its simplicity, this method is limited to the synthesis of mainly 1,2-trans products due to the participation of the 2-*O*-acyl group.^16^ It also lacks tolerance for acid-sensitive functional groups and is generally limited in the variety of commercially available thiols. Other methods, such as photoredox-mediated radical coupling, have come about, but they require large photosensitizers and more intricate thiosulfonate electrophiles.^17^ Recently, there has been an increase in the use of glycosyl thiols as a starting material, which subsequently allows for alkylation or arylation following thiolate formation. Glycosyl thiols are commonly obtained by first forming anomeric thiouronium salts or thioacetates, followed by hydrolysis and deprotection, respectively. One major advantage of using glycosyl thiols is the ability for predetermination of the stereochemistry at the anomeric center since glycosyl thiols are configurationally stable under most reaction conditions. However, the aforementioned routes to glycosyl thiols generally start from acylated sugars and, hence, give mainly the 1,2-trans products. Though not as concise, the available methods for stereoselective synthesis of 1,2-*cis* glycosyl thiols has continued to grow,^18^ meaning their use as precursors to 1,2-*cis* thioglycosides remains one of the best routes to access these challenging substrates.

While alkylation of glycosyl thiols can proceed via facile S_N_2 type reactions,^19^ conjugation with aryl groups is not as simple. Transition-metal-catalyzed cross-coupling of thiolates with aryl halides was first reported by Migita and co-workers^20^ and has since been reported using a broad range of catalysts and substrates.^21^ As such, methods for the synthesis of aryl thioglycosides from glycosyl thiols have emerged in response. Messaoudi and co-workers have contributed many advancements in the area, applying the palladium-catalyzed Buchwald–Hartwig–Migita reaction to the synthesis of thioglycosides.^22^ While these methods could be used to synthesize a broad range of compounds in very good yields, palladium is an expensive and rare metal, for which reason alternative syntheses have been highly sought. Efforts in copper-catalyzed couplings have afforded successful arylation of glycosyl thiols but have tended to be limited by the need for directing groups on the aryl moieties that require subsequent cleavage and greatly limit the reaction scope.^23^

Nickel has risen to the forefront as a purportedly cheap and abundant alternative to palladium in transition metal catalysis.^24^ Despite its catalytic behavior being less well-defined^25^ versus the strict Pd(0)/Pd(II) processes,^26^ many nickel-catalyzed C–S coupling methods have emerged recently, including some methods involving glycosyl thiols.^27^ Of particular interest is the recent advancement by the Lipshutz group,^28^ which utilizes nickel catalysis to perform S(alkyl)–C(aryl) couplings. The reactions proceed smoothly under mild conditions using an air- and moisture-stable nickel(II) precatalyst. Crucially, the work showcases the use of the α-tocopherol-derived surfactant TPGS-750-M also designed by Lipshutz,^29^ allowing the use of water as a reaction medium. As a recent life cycle assessment has shown, bypassing organic solvents actually has a much greater contribution to reducing the environmental impact of a process than changing the metal catalyst alone.^30^ Thus, performing nickel catalysis in an aqueous medium represents a two-factor improvement in the cost (both monetary and environmental) of the reaction versus traditional Migita coupling.

With our group’s ongoing interest in thioglycosides,^5a,6a,31^ the application of this methodology to reactions with glycosyl thiols represents an opportunity for a method to synthesize aryl thioglycosides, which bypasses the need for expensive rare-earth metals, high temperatures, or harmful organic solvents such as DMF or dioxane. Thus, we present herein a method for the cross coupling of glycosyl thiols with aryl halides using a bis-phenanthrolino nickel catalyst carried out in an aqueous surfactant solution.

## 2 Results and Discussion

**Scheme 1:**
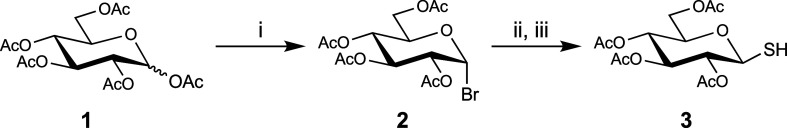
Synthesis of Glycosyl Thiol **3** (i) HBr (33% in AcOH), CH_2_Cl_2_, RT, 2 h; (ii) thiourea, acetone, reflux, 18 h; (iii) Na_2_S_2_O_5_, CH_2_Cl_2_/H_2_O 2:1, 50 °C, 15 h.

Glycosyl thiol **3** was obtained from per-acetylated glucose in three consecutive steps (Scheme 1). The simply protected sugar was chosen initially as it is easy to access and allows facile protecting group manipulation postcoupling. Thiosugar **3** was then utilized in a proof-of-concept reaction which consisted of a mixture of the sugar, base (Cs_2_CO_3_), zinc, iodobenzene (1 equiv), nickel(II) bromide (1 mol %), and 1,10-phenanthroline (2 mol %) in 2 wt % aqueous TPGS-750-m (0.25 M). The reaction bolstered the desired phenyl thioglycoside, however, in low yield (22%).

In the test reaction, the aggregation of the solids around the stir bar was observed, which may have contributed in part to the low yield. Going forward, an ultrasonic bath was used for mixing, which led to a much better emulsion. Consequently, ultrasonication inherently leads to heating of the reaction to approximately 45 °C; thus, reactions are reported at this temperature. The initial reaction relied on catalyst self-assembly in situ, which possibly contributed to low yield. Thus, the bis-phenanthrolino-nickel(II) bromide (Ni(phen)_2_Br_2_) catalyst was next synthesized ex situ by refluxing NiBr_2_ with 1,10-phenanthroline in acetonitrile.^28^ The bright green crude material was filtered off and then recrystallized from hot methanol to yield the deep green crystalline product. With the crystalline catalyst in hand, the reaction was carried out again to a modest improvement in yield (34%). Subsequently, various reaction parameters were systematically investigated to optimize the reaction (Table 1).

**Table 1:**
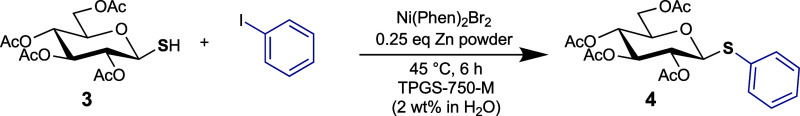
Optimization of the Coupling of **3** with Iodobenzene

entry	[catalyst] (mol %)^a^	conc. [M]^a^	base (equiv)	equiv PhI	yield (%)^b^
1	1	0.25	K_3_PO_4_ (1.2)	1	34
2	3	0.25	K_3_PO_4_ (1.2)	1	42
3	5	0.25	K_3_PO_4_ (1.2)	1	65
4	7.5	0.25	K_3_PO_4_ (1.2)	1	54
5	5	0.125	K_3_PO_4_ (1.2)	1	63
6	5	0.5	K_3_PO_4_ (1.2)	1	54
7	5	0.25	K_3_PO_4_ (2)	1	57
8	5	0.25	Cs_2_CO_3_(1.2)	1	61
9	5	0.25	KO^*t*^Bu(1.2)	1	59
**10**	**5**	**0.25**	**K**_**3**_**PO**_**4**_ (**1**.**2**)	**3**	**88**
11	5	0.25	K_3_PO_4_ (1.2)	5	78

a With respect to glycosyl thiol **3**.

b Isolated yield.

The table shows that the greatest overall improvement in yield was achieved by adjustment of the catalyst loading to 5 mol %, above which no significant change was observed. Increasing the amount of iodobenzene present in the reaction further improved the yield to an excess of 85%. Other factors, including the reaction concentration and the base (identity or amount), showed no significant effect on the yield of the phenyl thioglucoside **4** obtained from the reaction. Further, the necessity of both the catalyst and the zinc were demonstrated in control experiments: in the absence of either metal, the reaction did not proceed, and when using unligated nickel(II) bromide, the reaction returned only a very low yield (Table 2).

**Table 2:**
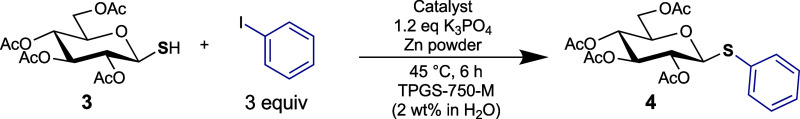
Catalyst Investigation

entry	catalyst (mol %)	Zn equiv	yield (%)^a^
1	Ni(phen)_2_Br_2_ (5)	0.25	88
2	Ni(phen)_2_Br_2_ (5)		
3	NiBr_2_ (5)	0.25	3
4		0.25	
5	1,10-phenanthroline (10)	0.25	

a Isolated yield.

Next, the reaction was attempted in organic solvents while maintaining all other variables to the optimized conditions (as in Table 1, entry 10). In either THF or toluene, no product was observed by TLC or crude ^1^H NMR. Solubility of potassium phosphate in the organic solvents was recognized as an issue, thus the reaction was repeated using a solution of potassium *tert*-butoxide (1 M in THF) as the base, but still no product was observed after heating overnight. Thus, it is evident that the micellar environment promotes the reaction, probably due to the close proximity of the reagents.

With the optimized conditions at hand, we next investigated the tolerance of the reaction toward different protecting groups. 2,3,4,6-Tetra-*O*-benzyl-β-d-glucopyranosyl thiol led to the formation of an inseparable mixture of the glycosyl disulfide as the major product and the desired phenyl thioglucoside in small amounts. It is not uncommon to observe spontaneous disulfide formation in the presence of oxygen, especially considering the electron-rich nature of benzylated pyranose rings; however, the reaction mixture was thoroughly degassed and a nitrogen atmosphere was employed. In this case, the presence of zinc and nickel metals may result in single-electron redox processes or formation of glycosyl metal sulfides that could promote disulfide formation via generation of S-radicals or attack by thiolates at the electrophilic S, respectively.^31d,32^ A corresponding benzoyl-protected glucosyl thiol was also employed in the reaction but did not lead to the identification of the desired product from a complex mixture. One factor in this may be the larger size of the benzoylated sugar compared with the others used, as it appeared poorly emulsified in the surfactant. Repeating the reaction at a lower concentration in an attempt to improve the solubility returned the same result. As such, we chose to maintain the acetyl-protecting group pattern.

During the initial expansion of the substrate scope, we noted an obvious trend in yield in relation to the electron richness of the aryl ring, but only when the aryl halide was liquid (Figure S1). Yields were significantly lower with solid aryl halides regardless of the electronic properties of the aryl substituent. We postulated that in these cases solubility was likely a limiting factor and repeated the experiments with the solid aryl halides as 10 M solutions in tetrahydrofuran, to which we saw improvements in yield of up to 27%. Thus, we amended our optimized conditions such that all solid aryl halides be added as a solution. Where the solubility of the aryl halide in THF did not allow the addition as a solution, the aryl halide and THF were added separately to the reaction mixture, still to the effect of improved yields.

Expansion of the substrate scope led to the synthesis of a number of substituted aryl β-thioglucosides (Table 3). Both *ortho*- and *para*-tolyl thioglucosides (**5** and **6**, respectively) were obtained in very good yields of 69%. The use of **5** as a glycosyl donor is well-reported in literature.^33^ Both aryl bromides and aryl iodides could be employed with good results. In most cases, introducing substituents onto the phenyl ring led to a slight decrease in yield compared to the phenyl thioglycoside, with the exception being the *para*-methoxyphenyl thioglucoside **8**, which was obtained in a yield of 96%. Most oxygen-containing substituents, including even a free phenolic group, were also well tolerated. Coupling of thiol **3** with 4-iodophenol produced compound **7** in a high yield (86%), and thioglycoside **9** could be formed in a very good yield (77%) from 3,4-dimethoxy-1-bromobenzene. The diethyl acetal substituted compound **10** was isolated in a moderate yield of 50% in part due to partial hydrolysis of the acetal functionality during purification.

**Table 3:**
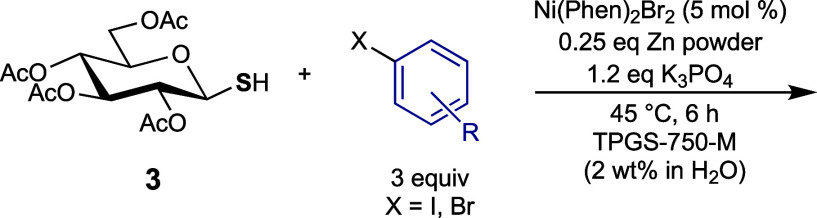

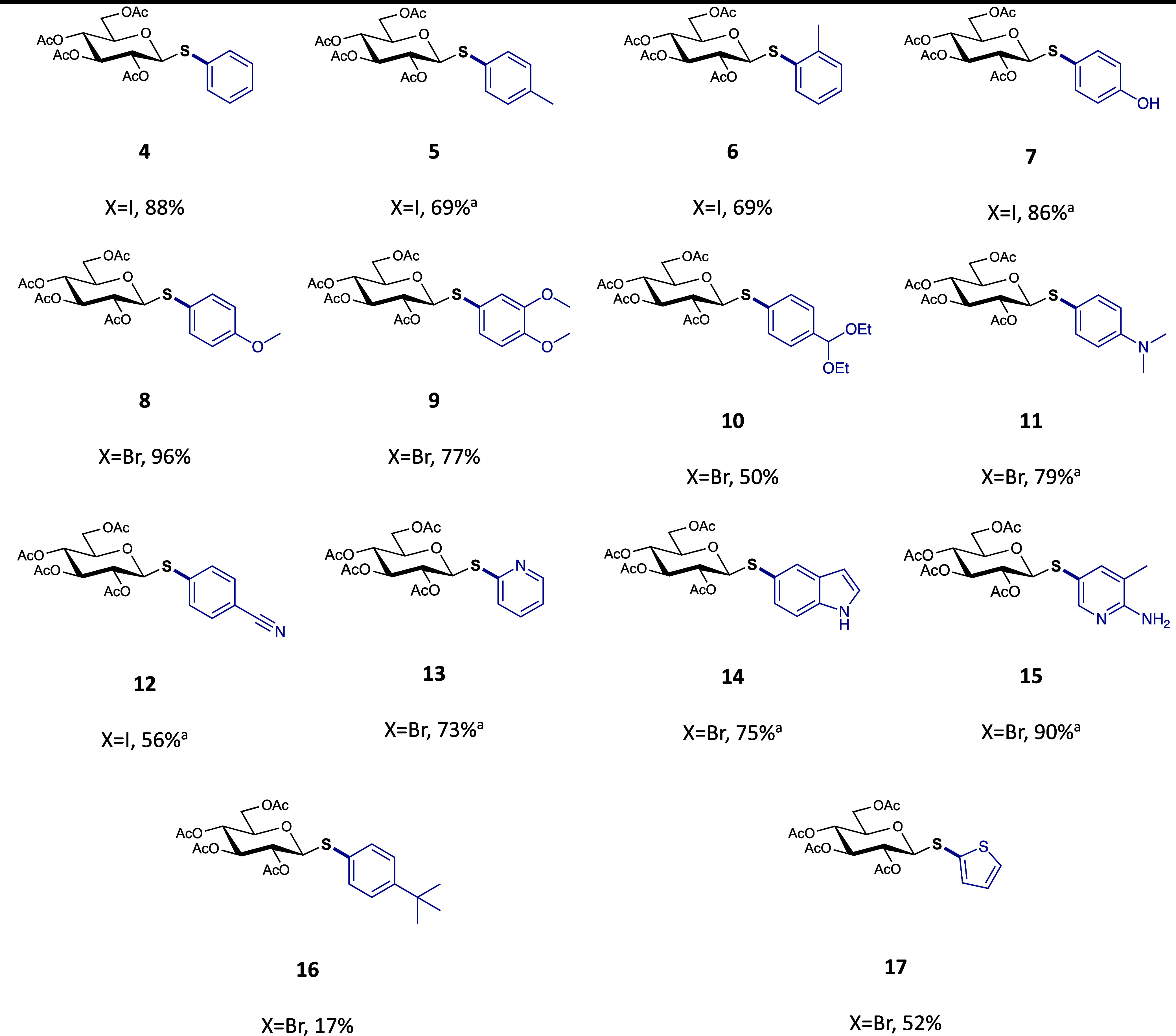
Substrate Scope of the Cross Coupling of Acetylated β-Glucosyl Thiol **3**^a^

a Aryl halide was added as 10 M solution in THF.

Nitrogen-containing substituents as well as *N*-heterocycles were also very well tolerated under the optimized conditions. Treatment of **3** with 4-bromo-*N*,*N*-dimethyl aniline gave compound **11** in a 79% yield. Coupling with *para*-iodobenzonitrile led to the isolation of compound **12** in relatively good yield, with the cyano group as a handle available for further derivatization of the compound. Synthesis of pyridiyl thioglucoside **13** from **3** and 2-bromopyridine proceeded smoothly and in high yield (73%). Pyridyl thioglycosides like **13** have been shown previously to undergo remote activation, which can lead to α-selective glycosylations.^34^ The reaction conditions could bypass the need for protection of active nitrogens such as the indole nitrogen in 5-bromoindole or the free amino group of 2-amino-5-bromo-3-methylpyridine. The aforementioned aryl halides were used as supplied to synthesize compounds **14** and **15**, respectively, in very good yields (75% and 90%, respectively). 2-Thienyl thioglucoside **17** was also generated in a relatively good yield (52%). Substituted 2-thienyl thioglycosides have been shown recently to possess antiviral activity, making **17** an interesting structure for possible further derivatization.^15b^ Under the same conditions, the bulky *para*-*tert*butyl phenyl thioglucoside **16** was the lowest yielding isolated product. We postulate this may be due to steric hindrance, considering that the alkyl substituent should be somewhat activating in this reaction.

**Scheme 2:**
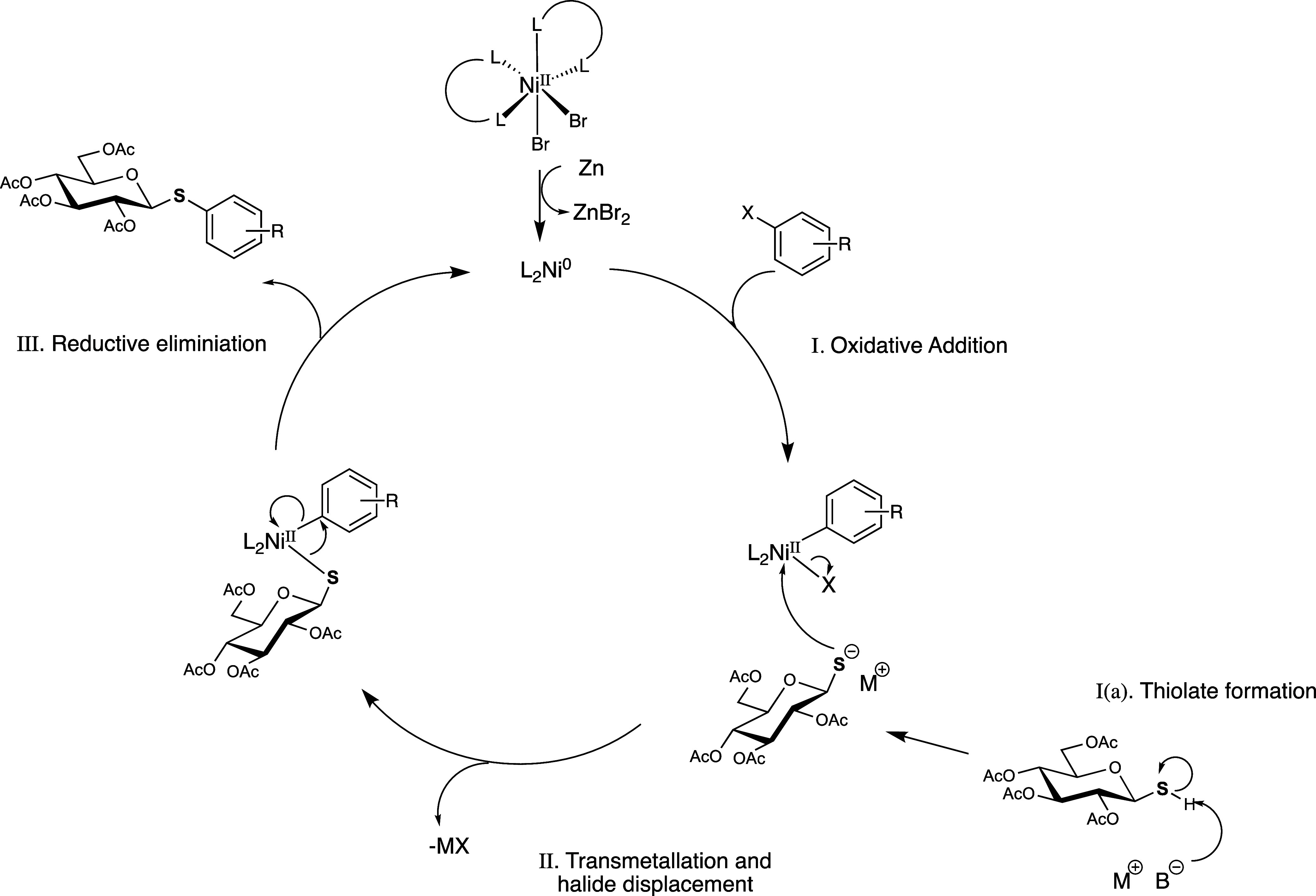
Proposed Catalytic Cycle for the Formation of Aryl Thioglucosides, L = 1,10-Phenanthroline

Overall, the tolerance of the system to various substituent groups appears to be correlated to the electronic properties of the substituent groups. Electron-withdrawing substituents performed more poorly: coupling with 4-iodobenzonitrile to generate compound **12** initially led to one of the lowest isolated yields of product, and the even more electron-deficient aryl halides 4-iodonitrobenzene and iodopentafluorobenzene both failed to deliver the desired products. The trend in the electronic effect is consistent with the assumption that nickel-catalyzed systems are rate-limited in the reductive elimination step, meaning electron-poor aryl rings will be destabilized as positive charge develops on the ring.^35^ Taking into account this observation, we have proposed a catalytic cycle which proceeds via an oxidative addition-transmetalation-reductive elimination pathway similar to a palladium-catalyzed reaction (Scheme 2).

Despite no formation of the *para*-nitrophenyl or pentafluorophenyl thioglucosides mentioned above, full consumption of starting material **3** was observed in the unsuccessful reactions. Similarly, reactions which returned moderate or low yields showed no remaining starting material when analyzed by TLC. The major side product in any of these reactions which could be identified by ^1^H NMR was glycosyl disulfide, which may be formed in side reactions, as mentioned previously. Disulfide formation competes with the cross-coupling; thus, in the case of electron-deficient aryl halides, a lower turnover frequency caused by slower reductive elimination may allow more of the thiolate species to be available for the homocoupling reaction.

**Scheme 3:**
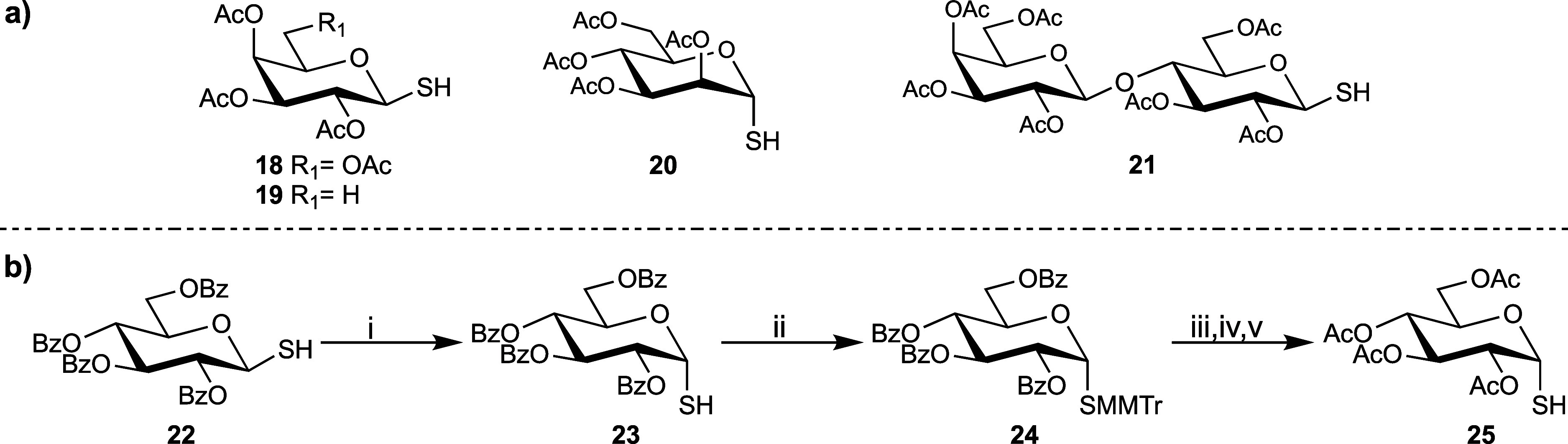
(a) Prepared Glycosyl Thiols **18–21**; (b) Synthesis of α-Glucosyl Thiol **25** (i) Cu(OTf)_2_, CH_2_Cl_2_, rt, 5 h; (ii) MMTrCl, pyridine, rt, 18 h; (iii) NaOMe, MeOH, rt, 3 h; (iv) Ac_2_O, pyridine, rt, 5 h; (v) TFA, Et_3_SiH, CH_2_Cl_2_, 0 °C-rt, 24 h.

We next expanded our investigation of the cross-coupling reaction to sugars beyond β-glucosyl thiol. Galactose, fucose, mannose, and lactose-derived glycosyl thiols **18**–**21** (Scheme 3a) were obtained by the same method as for **3**: treatment of the appropriate glycosyl bromide with thiourea followed by hydrolysis with alkali metal bisulfite. To obtain the α-anomer of **3**, the benzoylated β-glucosyl thiol **22** was epimerized with catalytic Cu(OTf)_2_, followed by protecting group manipulation to achieve the acetylated α-glycosyl thiol **25** (Scheme 3b).

With the array of glycosyl thiols in hand, their transformation to aryl thioglycosides by the optimized conditions (Table 1, entry 10) was investigated. The results are summarized in Tables 4–6. α-Thiol **25** was first subjected to the reaction conditions; unsurprisingly, we observed diminished yields compared to the β-counterparts (Table 4). Coupling of **25** with iodobenzene afforded the desired thioglucoside **26** in a pleasing 62% yield. This represents a concise method for phenyl α-thioglycosides, which remain difficult targets despite their utilization and distinct reactivity in oligosaccharide synthesis.^5,36^ Similarly, the coupling of **25** with 4-iodotoluene returned thioglycoside **27** in a good yield (58%). Subsequently, **25** was treated with 2-bromopyridine, which produced the pyridyl α-thioglucoside **28** in a yield of 22%. When coupled with 4-bromoanisole, **25** was converted to the *para*-methoxyphenyl α-thioglucoside **29** in 36% yield. The results for both **28** and **29** show a stark contrast to the excellent results obtained from the corresponding β-thioglucosides **13** and **8**, respectively. The same reaction was again performed using 4-iodophenol as the coupling partner, which furnished **30** in a 40% yield, once again significantly lower than the 80% yield obtained for β-thioglucoside **7**. The synthesis of **30** represents the first synthesis of a thioglycoside analogue of α-arbutin. While *S*-arbutin (the deacetylated version of **7**) has been reported and demonstrated comparable behavior to the natural arbutin *O*-glycoside,^37^ no investigation of α-*S*-arbutin has been carried out despite the increased activity observed in α-arbutin versus arbutin.^38^ Arbutin and α-arbutin, glycosylated hydroquinone derivatives, are antimelanogenic tyrosinase inhibitors that are used widely in the cosmetic industry.^39^ It should be noted that despite the potential activity of the phenolic hydroxyl group, no side products beyond the glycosyl 1,1-disulfide were observed. The precise mechanistic details underlying the different behavior observed between the α- and β-thiols are not clear. α-Thiosugars are generally considered less nucleophilic due to their axial configuration, and hence lessened S-lone pair repulsion compared to their equatorial epimers.^40^ As well, this configuration of α-glucose makes the C-1 substituent less accessible due to steric crowding, not only in relation to the 1,3-diaxial interactions of the pyranosyl ring but also due to the 1,2-cis relative configuration. These factors both may significantly inhibit the approach and attack of the sugar thiolate to the aryl–nickel–halide complex, which is already relatively crowded due to the smaller atomic radius of nickel and shorter metal–ligand bond lengths compared to palladium complexes.^41^ In addition, the trend in yield relative to the aryl substituents which was observed with the β-glucosyl thiol did not seem to extend to the anomer: the yield of the *para*-methoxyphenyl α-thioglucoside (having the most electron-rich aryl group) was only 36%, lower than that of either the phenyl or tolyl α-thioglucoside. This suggests an alternative limiting factor in the catalytic cycle besides the rate of reductive elimination. Despite the relatively low yields for some of the synthesized α-thioglycosides, a noteworthy advantage of this procedure is that it provides access to aryl α-1,2-*cis* thioglycosides with clean α-configuration, which is difficult to accomplish by other methods. Aryl α-thiomannosides **31** and **32**, which also have the anomeric group in the axial position, returned slightly higher yields, 68% and 61%, respectively, compared to α-glucosyl analogues **26** and **27** (Table 4). The 1,2-*trans* relative configuration in α-mannosides may help alleviate some steric hindrance.

**Table 4:**
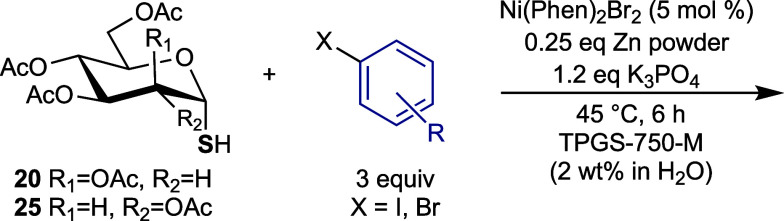

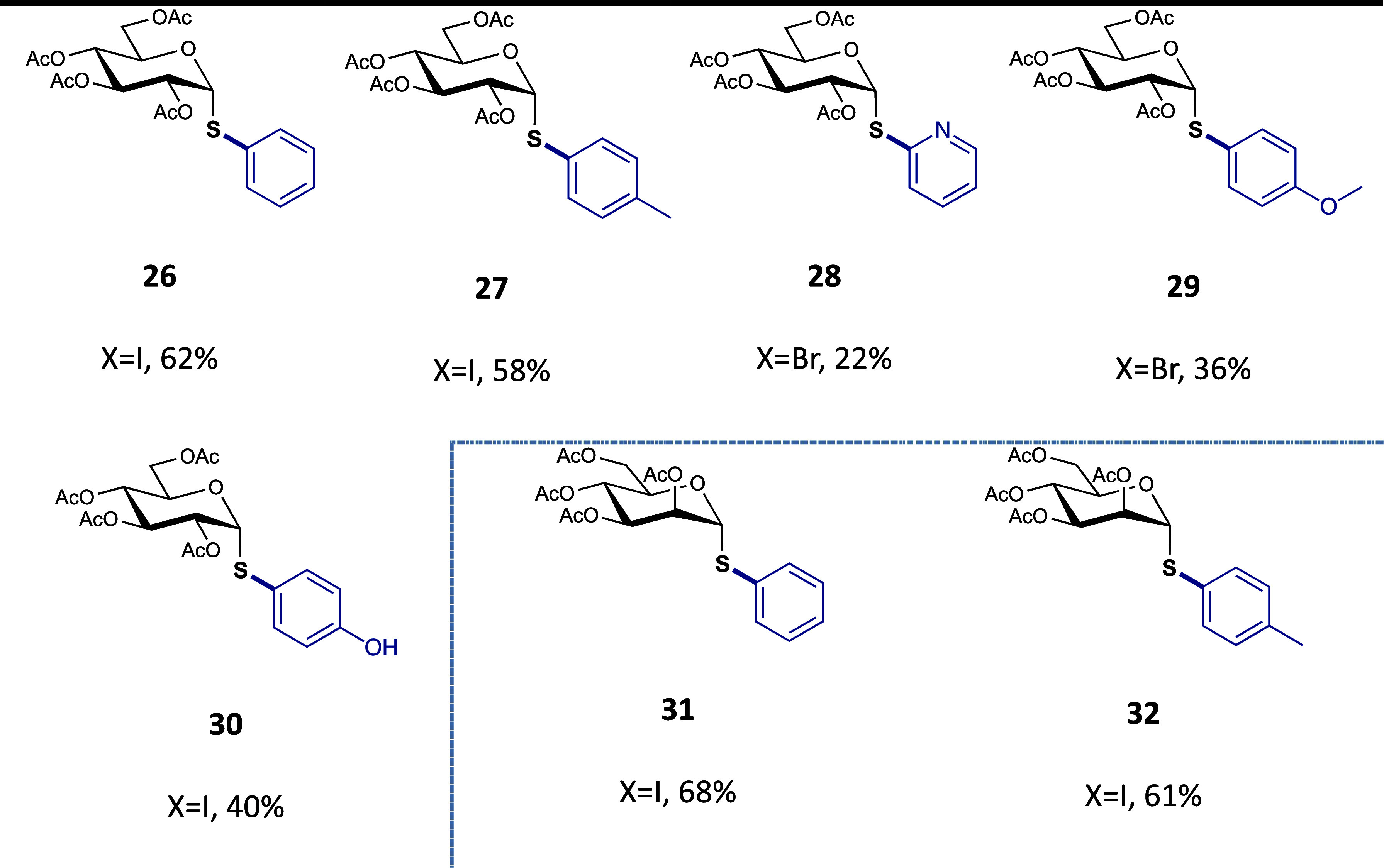
Cross Coupling Products Derived from Glycosyl Thiols **20** and **25**

**Table 5:**
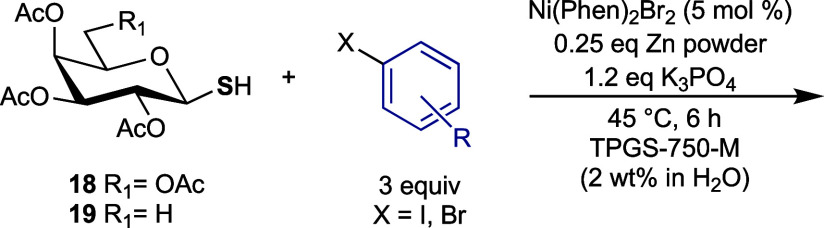

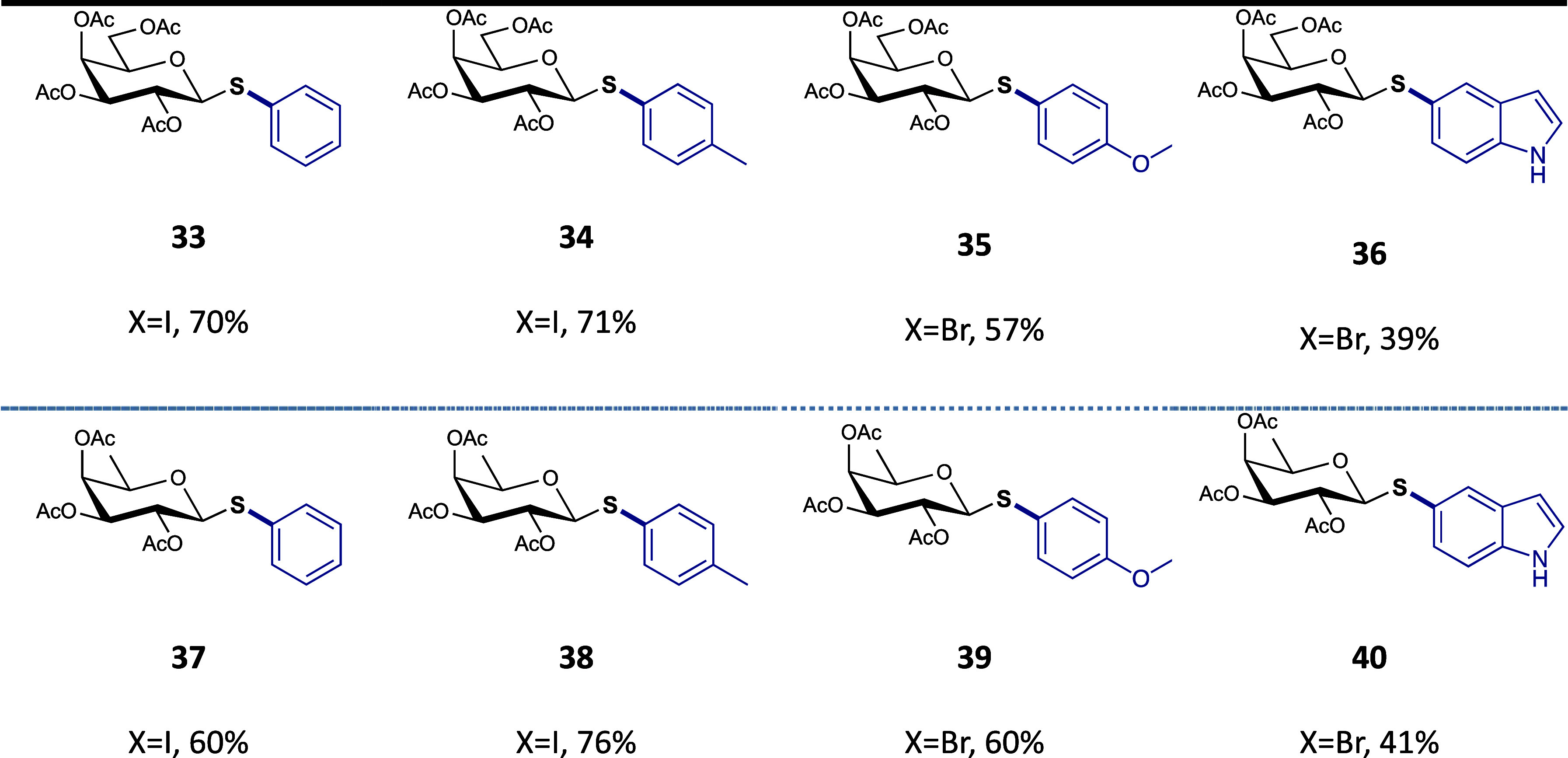
Cross Coupling Products Derived from Glycosyl Thiols **18** and **19**

**Table 6:**
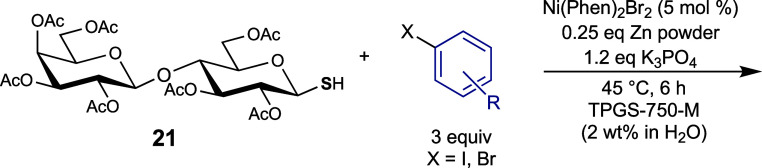

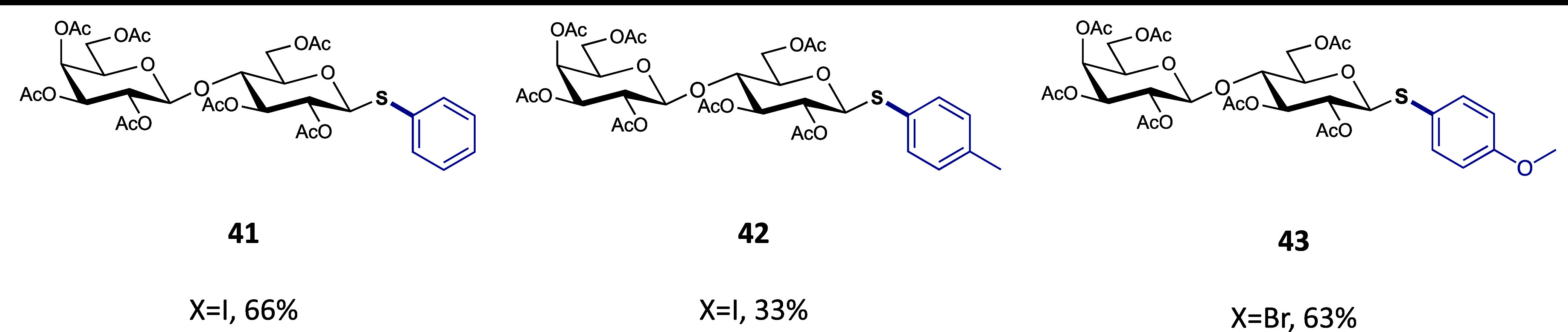
Cross Coupling Products Derived from Lactosyl Thiol **21**

To further explore the scope of this new procedure in the synthesis of aryl thioglycosides, we proceeded to use β-galactosyl thiol **18** as a substrate (Table 5). The coupling of **18** with iodobenzene under the optimized conditions afforded phenyl thioglycoside **33** as expected in a very good yield (70%). *para*-Tolyl thiogalactoside **34** was obtained from the coupling in a similarly high yield of 71%. When **18** was coupled with 4-bromoanisole or 5-bromoindole to form compounds **35** and **36**, respectively, the desired products were obtained in moderate yield (57% for **35** and 39% for **36**). Next, β-d-fucosyl thiol **19** was used as the substrate and was coupled with the same set of aryl halides, as indicated in Table 5. Phenyl and tolyl thiofucosides **37** and **38**, both used as glycosyl donors, could be synthesized in 60% and 76% yields, respectively. Coupling of thiol **19** with 4-bromoanisole returned compound **39** in 60% yield. Likewise, indolyl thiofucoside **40** was isolated in a moderate yield of 41% under the same reaction conditions. While the yields of the tolyl glycosides obtained from **18** and **19** were comparable to the yield of tolyl β-thioglucoside **5**, the heteroatom-substituted and heterocyclic thiogalactosides and thiofucosides showed diminished yields compared with the corresponding β-glucosides. The presence of the C-6 acetyl group appeared to have little effect on the reaction given the similarity of results between galactosyl and fucosyl substrates. Thus, the axial C-4 substituent most likely dictates the deviation of results versus the aryl β-thioglucosides, but whether the effect is steric, electronic, or a combination thereof is unknown.

Finally, we reasoned that this cross-coupling procedure could be extended to disaccharide-derived thiols. Indeed, as demonstrated by the synthesis of aryl thiolactosides **41**–**43** (Table 6), the reactions succeeded. Reaction of β-lactosyl thiol **21** with iodobenzene furnished the desired phenyl thiolactoside **41** in a 66% yield. Coupling of **21** with 4-iodotoluene was carried out under the same conditions, but unfortunately only afforded product **42** in 33% yield. In this case, the lower yield is most likely a result of the size of the lactose molecule as compared to glucose. At the concentration of surfactant solution employed in the optimized conditions, the sugar was poorly emulsified. Repeating the experiment at a lower overall concentration still did not improve the yield of **42** obtained. However, coupling **21** with 4-bromoanisole under the optimized conditions led to a good yield of 63% of the desired product **43**.

## 3 Conclusions

This new cross-coupling reaction which we have applied to the synthesis of a range of aryl and heteroaryl thioglycosides represents a useful method in carbohydrate chemistry. The nickel-catalyzed reaction reduces the reliance on other metals, such as palladium, for this type of reaction. Additionally, the surfactant-mediated reaction is not only accelerated by in situ micelle formation but also is carried out in aqueous media because of this. The mild reaction conditions also tolerate a wide range of functional groups, which means the relatively simple aryl groups included herein could be further functionalized to build more complex aglycon units. Moreover, one of the major advantages of synthesizing thioglycosides by cross-coupling of glycosyl thiols is the ability to access 1,2-*cis* thioglycosides cleanly.

## 4 Experimental Section

### 4.1 General Information

All chemicals were used as purchased, unless otherwise specified. Dry dichloromethane was obtained by distillation over calcium hydride. Dry methanol was obtained by drying it over flame-activated 3 Å molecular sieves. TLC analysis was carried out on silica gel 60 F_254_ Al-backed plates. Plates were visualized by charring after staining with 8% H_2_SO_4_ in MeOH. Column chromatography was carried out using silica 60 0.04–0.063 mm gel. Cross-coupling reactions were carried out in a Fisherbrand Easy 30H ultrasonic bath. ^1^H and ^13^C NMR spectroscopy was carried out on a Varian VnmrS spectrometer at 400 (101 MHz) or 500 (126 MHz) for ^1^H (^13^C). All spectra were collected at 25 °C. Samples were prepared in chloroform-*d* with 0.03% (v/v) TMS. ^1^H spectra were referenced to the residual chloroform at 7.26 ppm. ^13^C spectra were referenced to chloroform at 77.16 ppm. Chemical shifts (δ) are reported in parts per million (ppm). Coupling constants (*J*) are reported in Hz. Melting points were assessed using a Stuart SMP10 melting point apparatus and are reported uncorrected. High-resolution mass spectrometry was carried out on an Agilent 6546 Q-ToF equipped with an Agilent 1260 Infinity Prime II LC system. Results were processed by using Agilent MassHunter software. Total elemental analysis was performed on an Exeter Analytical CE-440 elemental analyzer.

### 4.2 General Procedure for Synthesis of Acetylated 1,2-*trans* Glycosyl Thiols

A fully acetylated sugar (7.5 mmol) was dissolved in dry dichloromethane (0.25 M). The solution was cooled to 0 °C and HBr solution (33% in AcOH) (45 mmol, 6 equiv) was added. The solution was allowed to obtain room temperature and was stirred for 2 h or until TLC indicated full consumption of the starting material. The reaction mixture was diluted with CH_2_Cl_2_ and then poured into water. The organic layer was successively washed with sat. aq NaHCO_3_, water, and brine. The organic layer was separated and then dried over MgSO_4_ and concentrated to yield a light-yellow oil. The oil was used directly in the next step: the crude material was dissolved in acetone (0.125 M) and thiourea (13.5 mmol, 1.8 equiv) was added. The solution was stirred at reflux in a DrySyn for 18 h. The solvent was removed in vacuo. Sodium metabisulfite (10.5 mmol, 1.4 equiv) was added to the flask, and the reagents were dissolved in a 2:1 CH_2_Cl_2_/H_2_O mixture (0.125 M). The solution was heated to 50 °C in a DrySyn for 18 h. The resulting mixture was added to a separating funnel, and the organic layer was collected. The aqueous layer was extracted 3× with CH_2_Cl_2._ The combined organic layers were then washed successively with water and brine before being dried over MgSO_4_ and concentrated in vacuo. The crude material was purified by flash column chromatography (3:1 cHex/EtOAc).

#### 4.2.1 2,3,4,6-Tetra-*O*-acetyl-1-thio-β-d-glucopyranose (**3**)

Glucose pentaacetate (3.00 g, 7.68 mmol) was treated according to the general procedure to yield the title compound as a colorless solid (2.05g, 5.61 mmol, 73%). ^1^H NMR (400 MHz, CDCl_3_): δ 5.19 (t, *J* = 9.3 Hz, 1H, H-3), 5.11 (t, *J* = 9.7 Hz, 1H, H-4), 4.98 (m, 1H, H-2), 4.55 (t, *J* = 9.8 Hz, 1H, H-1), 4.25 (dd, *J* = 12.5, 4.8 Hz, 1H, H-6a), 4.13 (dd, *J* = 12.5, 2.3 Hz, 1H, H-6b), 3.73 (ddd, *J* = 10.0, 4.8, 2.2 Hz, 1H, H-5), 2.32 (d, *J* = 10.0 Hz, 1H, SH), 2.10 (s, 3H, C(O)CH_3_), 2.08 (s, 3H, C(O)CH_3_), 2.03 (s, 3H, C(O)CH_3_), 2.01 (s, 3H, C(O)CH_3_). ^13^C{^1^H} NMR (101 MHz, CDCl_3_): δ 170.8, 170.2, 169.8, 169.5 (4× C=O), 78.9 (C-1), 76.5 (C-5), 73.7(C-3), 73.7 (C-2), 68.2 (C-4), 62.1 (C-6), 20.9, 20.9, 20.7, 20.7 (4× acetyl CH_3_). Spectral data matched literature reports.^18c^

### 4.3 General Procedure for the Cross-Coupling of Glycosyl Thiols with Aryl Halides

An acetylated glycosyl thiol (0.25 mmol, 1.0 equiv), Zn powder (7.5 mg, 0.25 equiv), bis-phenanthroline nickel(II) bromide (7.5 mg, 5 mol %), and K_3_PO_4_ (63.7 mg, 1.2 equiv) were added to a flask and dried under high vacuum for 30 min. The flask was flushed with N_2_ and added via a syringe were TPGS-750-M (2 wt % in H_2_O) (1.0 mL) and an aryl halide (0.75 mmol, 3.0 equiv). If the halide is solid, it was added as a 10 M solution in THF. The mixture was degassed under vacuum and backfilled with N_2_ five times. The flask was sealed with a rubber septum and sonicated in an ultrasonic bath at approximately 45 °C for 6 h. The reaction mixture was diluted with EtOAc (10 mL) and analyzed by TLC (eluent: cHex/EtOAc 2:1). The crude mixture was concentrated in vacuo onto silica gel and purified by column chromatography (cHex/EtOAc 2:1) to yield the thioglycoside product.

#### 4.3.1 Phenyl 2,3,4,6-Tetra-*O*-acetyl-1-thio-β-d-glucopyranoside (**4**)

Thiol **3** (95 mg, 0.25 mmol) and iodobenzene (84 μL, 0.75 mmol) were treated according to the general procedure and purified by column chromatography (cHex/EtOAc 2:1) to produce the title compound as a colorless solid (97 mg, 0.22 mmol, 88% yield). mp 116–117 °C. ^1^H NMR (500 MHz, CDCl_3_): δ 7.50–7.45 (m, 2H, Ar H), 7.31–7.26 (m, 3H, Ar H), 5.20 (td, *J* = 9.3, 0.8 Hz, 1H, H-3), 5.01 (ddd, *J* = 10.2, 9.5, 0.9 Hz, 1H, H-4), 4.94 (ddd, *J* = 10.1, 9.2, 1.0 Hz, 1H, H-2), 4.69 (d, *J* = 10.0 Hz, 1H, H-1), 4.20 (dd, *J* = 12.2, 5.1 Hz, 1H, H-6a), 4.15 (dd, *J* = 12.3, 2.1 Hz, 1H, H-6b), 3.75–3.67 (m, 1H, H-5), 2.06 (s, 3H), 2.05 (s, 3H), 1.99 (s, 3H), 1.96 (s, 3H) (4× acetyl CH_3_). ^13^C{^1^H} NMR (126 MHz, CDCl_3_): δ 170.6, 170.2, 169.4, 169.3 (4× C=O), 133.2 (Ar CH), 131.7 (Ar C–S), 129.0 (Ar CH), 128.5 (Ar CH), 85.7 (C-1), 75.8 (C-5), 74.0 (C-3), 70.0 (C-2), 68.3 (C-4), 62.2 (C-6), 20.8, 20.8, 20.6, 20.6 (4× acetyl CH_3_). Spectra matched literature reports.^16^

#### 4.3.2 *para*-Tolyl 2,3,4,6-Tetra-*O*-acetyl-1-thio-β-d-glucopyranoside (**5**)

Thiol **3** (95 mg, 0.25 mmol) and a solution of 4-iodotoluene (0.164 g, 0.75 mmol) in THF (75 μL, 10 M) were treated according to the general and purified by column chromatography (cHex/EtOAc 2:1) procedure to produce the title compound as a colorless solid (78 mg, 0.17 mmol, 69% yield). mp 111–113 °C. ^1^H NMR (400 MHz CDCl_3_): δ 7.39 (d, *J* = 8.1 Hz, 2H), 7.12 (dd, *J* = 8.6, 0.7 Hz, 2H), 5.20 (t, *J* = 9.4 Hz, 1H, H-3), 5.02 (dd, *J* = 10.1, 9.5 Hz, 1H, H-4), 4.93 (dd, *J* = 10.1, 9.3 Hz, 1H, H-2), 4.63 (d, *J* = 10.1 Hz, 1H, H-1), 4.21 (dd, *J* = 12.3, 4.8 Hz, 1H, H-6a), 4.17 (dd, *J* = 12.3, 2.7 Hz, 1H, H-6b), 3.70 (ddd, *J* = 10.1, 4.8, 2.7 Hz, 1H, H-5), 2.35 (s, 3H, PhCH_3_), 2.09, 2.08, 2.01, 1.98 (4× s, each 3H, acetyl CH_3_). ^13^C{^1^H} NMR (101 MHz, CDCl_3_): δ 170.7, 170.4, 169.5, 169.4 (4× C=O), 139.0 (ArC_q_), 134.0 (ArCH), 129.8 (ArCH), 127.7 (ArC_q_), 86.0 (C-1), 75.9 (C-5), 74.2 (C-3), 70.1 (C-2), 68.4 (C-4), 62.3 (C-6), 21.3 (Ph–CH_3_), 20.9, 20.9, 20.8, 20.7 (4× C(O)CH_3_). Spectra matched literature reports.^16^

#### 4.3.3 *meta*-Tolyl 2,3,4,6-Tetra-*O*-acetyl-1-thio-β-d-glucopyranoside (**6**)

Thiol **3** (95 mg, 0.25 mmol) and 2-iodotoluene (95 mL, 0.75 mmol) were treated according to the general procedure and purified by column chromatography (cHex/EtOAc 2:1) to produce the title compound as a colorless solid (79 mg, 0.17 mmol, 69% yield). mp 93–97 °C. ^1^H NMR (400 MHz, CDCl_3_): δ 7.52 (dt, *J* = 8.0, 0.9 Hz, 1H, ArH), 7.24–7.19 (m, 2H, ArH), 7.18–7.13 (m, 1H, ArH), 5.22 (t, *J* = 9.3 Hz, 1H, H-3), 5.06 (m, 2H, H-2, H-4), 4.67 (d, *J* = 10.1 Hz, 1H, H-1), 4.22 (dd, *J* = 12.3, 5.6 Hz, 1H, H-6a), 4.13 (dd, *J* = 12.2, 2.5 Hz, 1H, H-6b), 3.69 (ddd, *J* = 10.1, 5.5, 2.5 Hz, 1H, H-5), 2.40 (s, 3H, PhCH_3_), 2.09 (s, 3H), 2.07 (s, 3H), 2.02 (s, 3H), 2.00 (s, 3H) (4× acetyl CH_3_). ^13^C{^1^H} NMR (101 MHz, CDCl_3_): δ 170.7, 170.3, 169.5, 169.5 (4× C=O), 140.6 (ArC-CH_3_), 133.3 (ArCH), 132.1 (ArC-S), 130.6 (ArCH), 128.6 (ArCH), 126.8 (ArCH), 86.6 (C-1), 75.8 (C-5), 74.1 (C-3), 70.3 (C-2), 68.5 (C-4), 62.4 (C-6), 21.1 (PhCH_3_), 20.9, 20.8, 20.7, 20.7 (4× acetyl CH_3_). Spectra matched literature reports.^22a^

#### 4.3.4 *para*-Hydroxyphenyl 2,3,4,6-Tetra-*O*-acetyl-1-thio-β-d-glucopyranoside (**7**)

Thiol **3** (95 mg, 0.25 mmol) and a solution of 4-iodophenol (0.165 g, 0.75 mmol) in THF (75 μL, 10 M) were treated according to the general procedure and purified by column chromatography (cHex/EtOAc 2:1) to produce the title compound as a colorless solid (98 mg, 0.21 mmol, 86% yield). mp 136–139 °C. ^1^H NMR (400 MHz, CDCl_3_): δ 7.38 (d, *J* = 8.7 Hz, 2H, ArH), 6.78 (d, *J* = 8.7 Hz, 2H, ArH), 6.15 (s, 1H, PhOH), 5.19 (t, *J* = 9.4 Hz, 1H, H-3), 4.99 (t, *J* = 9.8 Hz, 1H, H-4), 4.88 (dd, *J* = 10.0, 9.2 Hz, 1H, H-2), 4.54 (d, *J* = 10.0 Hz, 1H, H-1), 4.19 (d, *J* = 3.7 Hz, 2H, H-6a + b), 3.68 (dt, *J* = 10.1, 3.7 Hz, 1H, H-5), 2.10, 2.07, 2.00, 1.98 (4× s, each 3H, C(O)CH_3_). ^13^C{^1^H} NMR (101 MHz, CDCl_3_): δ 171.1, 170.5, 169.8, 169.7 (4× C=O), 157.2 (Ar C–OH), 137.0 (Ar CH), 120.5 (Ar C–S), 116.1 (Ar CH), 85.6 (C-1), 75.8 (C-5), 74.2 (C-3), 70.1 (C-2), 68.3 (C-4), 62.2 (C-6), 21.0, 20.9, 20.7, 20.7 (4× acetyl CH_3_). HRMS (ESI+) calcd for C_20_H_24_O_10_SNa [M + Na]^+^, 479.0988; found, 479.0983.

#### 4.3.5 *para*-Methoxyphenyl 2,3,4,6-Tetra-*O*-acetyl-1-thio-β-d-glucopyranoside (**8**)

Thiol **3** (95 mg, 0.25 mmol) and 4-bromoanisole (94 mL, 0.75 mmol) were treated according to the general procedure and purified by column chromatography (cHex/EtOAc 2:1) to produce the title compound as a colorless solid (0.112 g, 0.24 mmol, 96% yield). mp 87–90 °C. ^1^H NMR (400 MHz, CDCl_3_): δ 7.44 (dd, *J* = 8.9, 0.7 Hz, 2H, ArH), 6.84 (dd, *J* = 9.0, 0.8 Hz, 2H, ArH), 5.19 (t, *J* = 9.4, 1H, H-3), 4.99 (t, *J* = 9.8 Hz, 1H, H-4), 4.88 (t, *J* = 9.5, Hz, 1H, H-2), 4.55 (d, *J* = 10.0 Hz, 1H, H-1), 4.23–4.14 (m, 2H, H-6a + b), 3.81 (s, 3H, OCH_3_), 3.71–3.64 (m, 1H, H-5), 2.10 (s, 3H), 2.07 (s, 3H), 2.00 (s, 3H), 1.98 (s, 3H) (4× acetyl CH_3_). ^13^C{^1^H} NMR (101 MHz, CDCl_3_): δ 170.7, 170.3, 169.5, 169.4 (4× C=O), 160.6 (Ar C–OMe), 136.7 (ArCH), 121.0 (Ar C–S), 114.5 (ArCH), 85.8 (C-1), 75.9 (C-5), 74.2 (C-3), 70.0 (C-2), 68.3 (C-4), 62.2 (C-6), 55.5 (PhOCH_3_), 20.9, 20.9, 20.7, 20.7 (4× acetyl CH_3_). Spectra matched literature reports.^22a^

#### 4.3.6 (3′,4′-Dimethoxyphenyl) 2,3,4,6-Tetra-*O*-acetyl-1-thio-β-d-glucopyranoside (**9**)

Thiol **3** (95 mg, 0.25 mmol) and 3,4-dimethoxybromobenzene (0.11 μL, 0.75 mmol) were treated according to the general procedure and purified by column chromatography (cHex/EtOAc 2:1) to produce the title compound as a colorless solid (96 mg, 0.19 mmol, 77% yield). mp 117–118 °C. ^1^H NMR (400 MHz, CDCl_3_): δ 7.08 (dd, *J* = 8.2, 2.1 Hz, 1H, phenyl H-6′), 7.05 (d, *J* = 2.1 Hz, 1H, phenyl H-5′), 6.81 (d, *J* = 8.3 Hz, 1H, phenyl H-2′), 5.19 (t, *J* = 9.4 Hz, 1H, H-3), 4.98 (dd, *J* = 10.1, 9.5 Hz, 1H, H-4), 4.89 (dd, *J* = 10.0, 9.3 Hz, 1H, H-2), 4.57 (d, *J* = 10.0 Hz, 1H, H-1), 4.23 (dd, *J* = 12.3, 4.7 Hz, 1H, H-6a), 4.16 (dd, *J* = 12.3, 2.4 Hz, 1H, H-6b), 3.89 (s, 3H, 4′-OCH_3_), 3.88 (s, 3H, 3′-OCH_3_), 3.68 (ddd, *J* = 10.1, 4.7, 2.4 Hz, 1H, H-5), 2.10 (s, 3H), 2.04 (s, 3H), 2.00 (s, 3H), 1.98 (s, 3H) (4× acetyl CH_3_). ^13^C{^1^H} NMR (101 MHz, CDCl_3_): δ 170.7, 170.3, 169.5, 169.4 (4× C=O), 150.2, 148.9 (Ar C–OMe), 128.4 (Ar CH), 120.7 (Ar C–S), 118.5, 111.3 (Ar CH), 85.5 (C-1), 76.0 (C-5), 74.2 (C-3), 70.0 (C-2), 68.2 (C-4), 62.2 (C-6), 56.1, 56.0 (2× OCH_3_), 21.0, 20.8, 20.7, 20.7 (4× acetyl CH_3_). Spectra matched literature reports.^42^

#### 4.3.7 *para*-α, α-Diethoxytolyl 2,3,4,6-Tetra-*O*-acetyl-1-thio-β-d-glucopyranoside (**10**)

Thiol **3** (95 mg, 0.25 mmol) and 4-bromobenzaldehyde diethyl acetal (155 μL, 0.75 mmol) were treated according to the general procedure and purified by column chromatography (cHex/EtOAc 2:1) to produce the title compound as a colorless solid (68 mg, 0.13 mmol, 50% yield). ^1^H NMR (400 MHz, CDCl_3_): δ 7.47 (d, *J* = 8.5 Hz, 2H, ArH), 7.41 (d, *J* = 8.1 Hz, 2H, ArH), 5.47 (s, 1H, PhCH(OEt)_2_), 5.21 (t, *J* = 9.3 Hz, 1H, H-3), 5.03 (dd, *J* = 10.1, 9.5 Hz, 1H, H-4), 4.97 (dd, *J* = 10.1, 9.2 Hz, 1H, H-2), 4.69 (d, *J* = 10.1 Hz, 1H, H-1), 4.21 (dd, *J* = 12.2, 5.1 Hz, 1H, H-6a), 4.16 (dd, *J* = 12.3, 2.6 Hz, 1H, H-6b), 3.71 (ddd, *J* = 10.1, 5.1, 2.6 Hz, 1H, H-5), 3.65–3.48 (m, 4H, ethyl CH_2_), 2.07 (2× s, 6H, acetyl CH_3_), 2.01 (s, 3H, acetyl CH_3_), 1.98 (s, 3H, acetyl CH_3_), 1.22 (t, *J* = 7.1 Hz, 6H, ethyl CH_3_). ^13^C{^1^H} NMR (101 MHz, CDCl_3_): δ 170.7, 170.3, 169.5, 169.4 (4× C=O), 139.6 (ArC_q_–CH(OEt)_2_), 132.9 (ArCH), 131.8 (ArC_q_-S), 127.4 (ArCH), 101.1 (CH(OEt)_2_), 85.9 (C-1), 75.9 (C-5), 74.1 (C-3), 70.1 (C-2), 68.3 (C-4), 62.3 (C-6), 61.3 (ethyl CH_2_), 20.9, 20.8, 20.7, 20.7 (4× acetyl CH_3_), 15.3 (ethyl CH_3_). HRMS (ESI+) calcd for C_25_H_34_O_11_SNa [M + Na]^+^, 565.1719; found, 565.1715.

#### 4.3.8 *para*-*N*,*N*-Dimethylanilino 2,3,4,6-Tetra-*O*-acetyl-1-thio-β-d-glucopyranoside (**11**)

Thiol **3** (95 mg, 0.25 mmol) and a solution of 4-bromo *N*,*N*-dimethyl aniline (0.150 g, 0.75 mmol) in THF (75 μL, 10 M) were treated according to the general procedure and purified by column chromatography (cHex/EtOAc 2:1) to produce the title compound as a colorless solid (95 mg, 0.20 mmol, 79% yield). mp 147–149 °C. ^1^H NMR (400 MHz, CDCl_3_): δ 7.41–7.32 (m, 2H, ArH), 6.62 (d, *J* = 8.9 Hz, 2H, ArH), 5.18 (t, *J* = 9.4 Hz, 1H, H-3), 4.99 (dd, *J* = 10.1, 9.5 Hz, 1H, H-4), 4.87 (dd, *J* = 10.0, 9.3 Hz, 1H, h-2), 4.49 (d, *J* = 10.0 Hz, 1H, H-1), 4.18 (dd, *J* = 3.7, 1.8 Hz, 2H, H6a + b), 3.65 (ddd, *J* = 10.1, 4.3, 3.1 Hz, 1H, H-5), 2.97 (s, 6H, N–CH_3_), 2.10 (s, 3H), 2.07 (s, 3H), 2.00 (s, 3H), 1.97 (s, 3H)(4× acetyl CH_3_). ^13^C{^1^H} NMR (101 MHz, CDCl_3_): δ 170.8, 170.4, 169.5, 169.4 (4× C=O), 151.1 (ArC-N), 136.8 (ArCH), 114.8 (ArC-S), 112.4 (ArCH), 86.2 (C-1), 75.8 (C-5), 74.3 (C-3), 70.1 (C-2), 68.4 (C-4), 62.3 (C-6), 40.4 (N–CH_3_), 21.0, 20.9, 20.8, 20.7 (4× acetyl CH_3_). HRMS (ESI+) calcd for C_22_H_30_NO_9_S [M + H]^+^, 484.1641; found, 484.1639.

#### 4.3.9 *para*-Cyanophenyl 2,3,4,6-Tetra-*O*-acetyl-1-thio-β-d-glucopyranoside (**12**)

Thiol **3** (95 mg, 0.25 mmol) and 4-iodobenzonitrile (0.172 g, 0.75 mmol) plus THF (75 μL) were treated according to the general procedure and purified by column chromatography (cHex/EtOAc 2:1) to produce the title compound as a colorless solid (66 mg, 0.14 mmol, 56% yield). mp 170–171 °C.·^1^H NMR (400 MHz, CDCl_3_): δ 7.62–7.51 (m, 4H, Ar–H), 5.25 (t, *J* = 9.3 Hz, 1H, H-3), 5.05 (dd, *J* = 10.1, 9.4 Hz, 1H, H-4), 5.01 (dd, *J* = 10.1, 9.2 Hz, 1H, H-2), 4.81 (d, *J* = 10.1 Hz, 1H, H-1), 4.23 (dd, *J* = 12.4, 5.3 Hz, 1H, H-6a), 4.17 (dd, *J* = 12.4, 2.5 Hz, 1H, H-6b), 3.79 (ddd, *J* = 10.1, 5.3, 2.5 Hz, 1H, H-5), 2.08 (s, 3H), 2.06 (s, 3H), 2.02 (s, 3H), 1.99 (s, 3H) (3× acetyl CH_3_). ^13^C{^1^H} NMR (101 MHz, CDCl_3_): δ 170.5, 170.2, 169.5, 169.3 (4× C=O), 139.3 (ArC_q_-S), 132.5 (ArCH), 131.6 (ArCH), 118.4 (ArC_q_-CN), 111.5 (−CN), 84.6 (C-1), 76.2 (C-5), 73.8 (C-3), 69.7 (C-2), 68.1 (C-4), 62.2 (C-6), 20.8, 20.8, 20.7 (acetyl CH_3_). Spectral data matched literature reports.^27d^

#### 4.3.10 (2-Pyridinyl) 2,3,4,6-Tetra-*O*-acetyl-1-thio-β-d-glucopyranoside (**13**)

Thiol **3** (95 mg, 0.25 mmol) and 2-bromopyridine (72 μL, 0.75 mmol) were treated according to the general procedure and purified by column chromatography (cHex/EtOAc 2:1) to produce the title compound as a colorless solid (80 mg, 0.18 mmol, 73% yield). mp 118–121 °C. ^1^H NMR (500 MHz, CDCl_3_): δ 8.50–8.40 (m, 1H, Py H-6), 7.54 (td, *J* = 7.7, 1.8 Hz, 1H, Py H-4), 7.23 (dt, *J* = 8.1, 1.0 Hz, 1H, Py H-3), 7.08 (ddd, *J* = 7.4, 4.9, 1.1 Hz, 1H, Py H-5), 5.78 (d, *J* = 10.5 Hz, 1H, H-1), 5.34 (t, *J* = 9.3 Hz, 1H, H-3), 5.20 (dd, *J* = 10.5, 9.2 Hz, 1H, H-2), 5.14 (dd, *J* = 10.1, 9.4 Hz, 1H, H-4), 4.25 (dd, *J* = 12.4, 4.8 Hz, 1H, H-6a), 4.09 (dd, *J* = 12.4, 2.3 Hz, 1H, H-6b), 3.87 (ddd, *J* = 10.1, 4.8, 2.3 Hz, 1H, H-5), 2.02 (s, 3H), 2.02 (s, 3H), 2.00 (s, 3H), 2.00 (s, 3H)(4× acetyl CH_3_). ^13^C{^1^H} NMR (126 MHz, CDCl_3_): δ 170.8, 170.3, 169.7, 169.6 (4× C=O), 155.3 (Py C-2), 149.6 (Py C-6), 136.9 (Py C-4), 123.7 (Py C-3), 121.1 (Py C-5), 81.8 (C-1), 76.0 (C-5), 74.2 (C-3), 69.6 (C-2), 68.4 (C-4), 62.1 (C-6), 20.8, 20.8, 20.7, 20.7(4× acetyl CH_3_). Spectra matched literature reports.^27c^

#### 4.3.11 (5-Indolyl) 2,3,4,6-Tetra-*O*-acetyl-1-thio-β-d-glucopyranoside (**14**)

Thiol **3** (95 mg, 0.25 mmol) and a solution of 5-bromoindole (0.147 g, 0.75 mmol) in THF (75 μL, 10 M) were treated according to the general procedure and purified by column chromatography (cHex/EtOAc 2:1) to produce the title compound as a light brown oil (90 mg, 0.19 mmol, 75% yield).^1^H NMR (500 MHz, CDCl_3_): δ 8.54 (broad s, 1H, N–H), 7.82 (q, *J* = 1.0 Hz, 1H, indole H-4), 7.31 (d, *J* = 1.2 Hz, 2H, indole H-6 and H-7), 7.22 (dd, *J* = 3.2, 2.4 Hz, 1H, indole H-2), 6.51 (dd, *J* = 3.3, 2.0 Hz, 1H, indole H-3), 5.20 (t, *J* = 9.4 Hz, 1H, H-3), 5.01 (t, *J* = 9.8 Hz, 1H, H-4), 4.94 (dd, *J* = 10.1, 9.3 Hz, 1H, H-2), 4.63 (d, *J* = 10.1 Hz, 1H, H-1), 4.21 (dd, *J* = 12.2, 4.9 Hz, 1H, H-6a), 4.16 (dd, *J* = 12.2, 2.5 Hz, 1H, H-6b), 3.64 (ddd, *J* = 10.1, 4.8, 2.5 Hz, 1H, H-5), 2.12 (s, 3H), 2.03 (s, 3H), 1.99 (s, 3H), 1.96 (s, 3H). ^13^C{^1^H} NMR (126 MHz, CDCl_3_): δ 170.8, 170.6, 169.5, 169.5 (4× C=O), 136.1 (indole C-8), 128.6 (indole C-9), 128.5 (indole C-6), 127.9 (indole C-4), 125.4 (indole C-2), 120.5 (indole C-7), 111.5 (indole C-3), 102.7, 86.8 (C-1), 75.7 (C-5), 74.2 (C-3), 70.2 (C-2), 68.3 (C-4), 62.2 (C-6), 20.9, 20.8, 20.7, 20.7 (4× acetyl CH_3_). Spectra matched literature reports.^27d^

#### 4.3.12 (2-Amino-3-methyl-5-pyridinyl) 2,3,4,6-Tetra-*O*-acetyl-1-thio-β-d-glucopyranoside (**15**)

Thiol **3** (95 mg, 0.25 mmol) and 2-amino-3-methyl-5-bromopyridine (0.176 g, 0.75 mmol) plus THF (75 μL) were treated according to the general procedure and purified by column chromatography (cHex/EtOAc 2:1) to produce the title compound as a light brown oil (0.11 g, 0.23 mmol, 90% yield). ^1^H NMR (500 MHz, CDCl_3_): δ 8.00 (dd, *J* = 2.3, 0.8 Hz, 1H, ArCH), 7.39 (dd, *J* = 2.2, 1.0 Hz, 1H, ArCH), 5.16 (t, *J* = 9.4 Hz, 1H, H-3), 4.95 (t, *J* = 9.8 Hz, 1H, H-4), 4.83 (dd, *J* = 10.0, 9.2 Hz, 1H, H-2), 4.71 (s, 2H, NH_2_), 4.43 (d, *J* = 10.0 Hz, 1H, H-1), 4.15 (m, 2H, H-6a + b), 3.63 (ddd, *J* = 10.1, 4.6, 2.7 Hz, 1H, H-5), 2.09 (s, 3H, pyridinyl CH_3_), 2.08 (s, 3H), 2.04 (s, 3H), 1.97 (s, 3H), 1.95 (s, 3H)(4× acetyl CH_3_). ^13^C{^1^H} NMR (126 MHz, CDCl_3_): δ 170.7, 170.3, 169.5, 169.3 (4× C=O), 157.9 (pyridinyl C-6), 152.4 (pyridinyl C-6), 144.7 (pyridinyl C-4), 116.6 (pyridinyl C-3), 114.1 (pyridinyl C-5), 85.3 (C-1), 75.8 (C-5), 74.1 (C-3), 69.8 (C-2), 68.2 (C-4), 62.0 (C-6), 20.9, 20.8, 20.7, 20.6 (4× acetyl CH_3_), 17.1 (pyridinyl CH_3_). HRMS (ESI+) calcd. for C_20_H_27_N_2_O_9_S [M + H]^+^, 471.1437; found, 471.1431.

#### 4.3.13 *para*-*tert*-Butylphenyl 2,3,4,6-Tetra-*O*-acetyl-1-thio-β-d-glucopyranoside (**16**)

Thiol **3** (95 mg, 0.25 mmol) and 4-bromo *tert*butyl benzene (130 μL, 0.75 mmol) were treated according to the general procedure and purified by column chromatography (cHex/EtOAc 2:1) to produce the title compound as a colorless solid (21 mg, 0.04 mmol, 17% yield). ^1^H NMR (400 MHz, CDCl_3_): δ 7.42 (d, *J* = 8.5 Hz, 2H, ArH), 7.33 (d, *J* = 8.5 Hz, 2H, ArH), 5.21 (t, *J* = 9.4 Hz, 1H, H-3), 5.04 (t, *J* = 9.8 Hz, 1H, H-4), 4.97 (dd, *J* = 10.1, 9.2 Hz, 1H, H-2), 4.66 (d, *J* = 10.1 Hz, 1H, H-1), 4.21 (m, 2H, H-6a + b), 3.76–3.68 (m, 1H, H-5), 2.09 (s, 3H), 2.08 (s, 3H), 2.02 (s, 3H), 1.99 (s, 3H) (4× acetyl CH_3_), 1.32 (s, 9H, ^*t*^Butyl CH_3_). ^13^C{^1^H} NMR (101 MHz, CDCl_3_): δ 170.7, 170.4, 169.6, 169.5 (4× C=O), 163.3 (ArC_q_), 152.0 (ArC_q_), 133.5 (ArCH), 126.2 (ArCH), 86.0 (C-1), 75.9 (C-5), 74.2 (C-3), 70.2 (C-2), 68.4 (C-4), 62.3 (C-6), 34.8 (^*t*^Butyl C_q_), 31.4 (^*t*^Butyl CH_3_), 20.9, 20.9, 20.8, 20.7 (4× acetyl CH_3_). Spectral data matched literature reports.^42^

#### 4.3.14 (2-Thienyl) 2,3,4,6-Tetra-*O*-acetyl-1-thio-β-d-glucopyranoside (**17**)

Thiol **3** (95 mg, 0.25 mmol) and 2-bromo thiophene (72 mL, 0.75 mmol) were treated according to the general procedure and purified by column chromatography (cHex/EtOAc 2:1) to produce the title compound as a colorless solid (58 mg, 0.13 mmol, 52% yield). mp 79–81 °C. ^1^H NMR (400 MHz, CDCl_3_): δ 7.43 (dd, *J* = 5.4, 1.3 Hz, 1H, thiophene H-3), 7.19 (dd, *J* = 3.6, 1.2 Hz, 1H, thiophene H-5), 7.00 (dd, *J* = 5.4, 3.6 Hz, 1H, thiophene H-4), 5.18 (t, *J* = 9.4 Hz, 1H, H-3), 4.99 (t, *J* = 9.8 Hz, 1H, H-4), 4.90 (dd, *J* = 10.0, 9.3 Hz, 1H, H-2), 4.49 (d, *J* = 9.9 Hz, 1H, H-1), 4.18 (m, 2H, H-6a + b), 3.69 (ddd, *J* = 10.1, 4.3, 2.9 Hz, 1H, H-5), 2.09 (s, 3H), 2.06 (s, 3H), 1.99 (s, 3H), 1.96 (s, 3H)(4× acetyl CH_3_). ^13^C{^1^H} NMR (101 MHz, CDCl_3_): δ 170.7, 170.3, 169.5, 169.3 (4× C=O), 136.7 (thiophene C-2), 131.7 (thiophene C-5), 127.6 (thiophene C-3), 127.1 (thiophene C-4), 85.5 (H-1), 75.9 (H-5), 74.0 (H-3), 69.7 (H-2), 68.1 (H-4), 62.1 (H-6), 20.9, 20.7, 20.6 (acetyl CH_3_). Spectral data matches literature reports.^22a^

### 4.4 Synthesis of Glycosyl Thiols **18–22**

#### 4.4.1 2,3,4,6-Tetra-*O*-acetyl-1-thio-β-d-galactopyranose (**18**)

Pentaacetyl galactose (1.95 g, 5 mmol) was treated according to the general procedure outlined in Section 4.2 and purified by column chromatography (cHex/EtOAc 4:1) to afford the title compound as a colorless solid (1.43 g, 3.92 mmol, 78% yield). ^1^H NMR (500 MHz, CDCl_3_): δ 5.44 (dd, *J* = 3.4, 1.3 Hz, 1H, H-4), 5.18 (t, *J* = 9.9 Hz, 1H, H-2), 5.02 (dd, *J* = 10.1, 3.4 Hz, 1H, H-3), 4.54 (t, *J* = 9.8 Hz, 1H, H-1), 4.17–4.10 (m, 2H, H-6a, H-6b), 3.95 (td, *J* = 6.6, 1.3 Hz, 1H, H-5), 2.38 (d, *J* = 9.9 Hz, 1H, SH), 2.17 (s, 3H, (CO)CH_3_), 2.09 (s, 3H, (CO)CH_3_), 2.05 (s, 3H, (CO)CH_3_), 1.99 (s, 3H,(CO)CH_3_). ^13^C{^1^H} NMR (126 MHz, CDCl_3_): δ 170.5, 170.3, 170.1, 170.0 (4× C=O), 79.3 (C-1), 75.1 (C-5), 71.7 (C-3), 71.0 (C-2), 67.4 (C-4), 61.6 (C-6), 21.0, 20.8, 20.8, 20.7 (4× acetyl CH_3_). Spectral data matched literature reports.^18c^

#### 4.4.2 2,3,4-Tri-*O*-acetyl-1-thio-β-d-fucopyranose (**19**)

d-Fucose tetraacetate (2.49 g, 7.50 mmol) was treated according to the general procedure outlined in Section 4.2 and purified by column chromatography (cHex/EtOAc 4:1) to afford the title compound as a colorless solid (1.86 g, 6.06 mmol, 81%). mp 116–117 °C. ^1^H NMR (400 MHz, CDCl_3_): δ 5.26 (dd, *J* = 3.4, 1.1 Hz, 1H, H-4), 5.14 (t, *J* = 9.9 Hz, 1H, H-2), 4.99 (dd, *J* = 10.1, 3.4 Hz, 1H, H-3), 4.48 (t, *J* = 9.8 Hz, 1H, H-1), 3.82 (qd, *J* = 6.4, 1.2 Hz, 1H, H-5), 2.31 (d, *J* = 9.9 Hz, 1H, SH), 2.16 (s, 3H), 2.06 (s, 3H), 1.96 (s, 3H) (3× acetyl CH_3_), 1.20 (d, *J* = 6.4 Hz, 3H, CH_3_-6). ^13^C{^1^H} NMR (101 MHz, CDCl_3_): δ 170.7, 170.1, 170.0 (3x C=O), 78.9 (C-1), 73.9 (C-5), 72.1 (C-3), 71.1 (C-2), 70.5 (C-4), 21.0, 20.8, 20.7 (3× acetyl CH_3_), 16.5 (C-6). HRMS (QTOF/ESI+) *m*/*z* calcd for C_12_H_18_O_7_SNa ([M + Na]^+^), 329.0671; found, 329.0665.

#### 4.4.3 2,3,4,6-Tetra-*O*-acetyl-1-thio-α-d-mannopyranose (**20**)

Mannose pentaacetate (1.95 g, 5 mmol) was treated according to the general procedure outlined in Section 4.2 and purified by column chromatography (cHex/EtOAc 5:1) to afford the title compound as a colorless solid (1.06 g, 2.90 mmol, 58% yield). ^1^H NMR (400 MHz, CDCl_3_): δ 5.51 (dt, *J* = 6.9, 0.9 Hz, 1H, H-1), 5.33–5.21 (m, 3H, H-2/H-3/H-4), 4.34–4.27 (m, 1H, H-5), 4.24 (dd, *J* = 12.2, 5.0 Hz, 1H, H-6a), 4.06 (dd, *J* = 12.2, 2.2 Hz, 1H, H-6b), 2.28 (d, *J* = 6.9 Hz, 1H, SH), 2.11 (s, 3H), 2.04 (s, 3H), 2.00 (s, 3H), 1.94 (s, 3H)(4× acetyl CH_3_). Spectral data matched literature reports.^18c^

#### 4.4.4 2,3,4,6-Tetra-*O*-acetyl-β-d-galactopyranosyl-(1 → 4)-2,3,6-tri-*O*-acetyl-1-thio-β-d-glucopyranose (**21**)

Lactose octaacetate (3.39 g, 5.0 mmol) was treated according to the general procedure outlined in Section 4.2 and purified by column chromatography (cHex/EtOAc 2:1) to obtain the title compound as a colorless solid (2.25 g, 3.37 mmol, 67%). ^1^H NMR (400 MHz, CDCl_3_): δ 5.34 (dd, *J* = 3.5, 1.2 Hz, 1H, H-4), 5.17 (t, *J* = 9.2 Hz, 1H, H-3′), 5.09 (dd, *J* = 10.4, 7.9 Hz, 1H, H-2′), 4.94 (dd, *J* = 10.4, 3.4 Hz, 1H, H-3), 4.87 (t, *J* = 9.6 Hz, 1H, H-2), 4.52 (t, *J* = 9.7 Hz, 1H, H-1′), 4.48–4.42 (m, 2H, H-1, H-6a), 4.12–4.04 (m, 3H, H-6b/H6a′/H-6b′), 3.86 (ddd, *J* = 7.5, 6.4, 1.2 Hz, 1H, H-5), 3.80 (dd, *J* = 10.0, 9.1 Hz, 1H, H-4′), 3.62 (ddd, *J* = 9.9, 5.2, 2.0 Hz, 1H, H-5′), 2.25 (d, *J* = 9.7 Hz, 1H, SH), 2.14 (s, 3H), 2.12 (s, 3H), 2.07 (s, 3H), 2.06 (s, 3H), 2.04 (2× s, 6H), 1.96 (s, 3H) (7× acetyl CH_3_). ^13^C{^1^H} NMR (101 MHz, CDC): δ 170.4, 170.3, 170.1, 170.0, 169.9, 169.6, 169.1 (7× C=O), 101.1 (C-1), 78.5 (C-1′), 77.2 (C-5′), 76.0 (C-4′), 73.9 (C-3′), 73.5 (C-3), 71.0 (C-5), 70.7 (C-2′), 69.1 (C-2), 66.6 (C-4), 62.2 (C-6), 60.8 (C-6′), 20.9, 20.8, 20.7, 20.6, 20.6, 20.5 (Acetyl CH_3_). Spectral data matches literature reports.^43^

#### 4.4.5 2,3,4,6-Tetra-*O*-benzoyl-1-thio-β-d-glucopyranose (**22**)

Glucose pentabenzoate (5.26 g, 7.5 mmol) was treated by the same general procedure outlined in Section 4.2. The crude product was purified by flash chromatography (cHex/EtOAc 6:1) to afford the title compound as a colorless solid (3.22 g, 5.25 mmol, 70% yield). ^1^H NMR (400 MHz, CDCl_3_): δ 8.08–8.00 (m, 2H, Ar–H), 8.00–7.91 (m, 2H, Ar–H), 7.89 (dd, *J* = 8.4, 1.3 Hz, 2H, Ar–H), 7.85–7.77 (m, 2H, Ar–H), 7.60–7.45 (m, 3H, Ar–H), 7.46–7.34 (m, 7H, Ar–H), 7.39–7.30 (m, 2H, Ar–H), 5.89 (t, *J* = 9.6 Hz, 1H, H-4), 5.71 (t, *J* = 9.8 Hz, 1H, H-2), 5.51 (t, *J* = 9.6 Hz, 1H, H-3), 4.90 (t, *J* = 9.7 Hz, 1H, H-1), 4.63 (dd, *J* = 12.3, 3.0 Hz, 1H, H-6a), 4.48 (dd, *J* = 12.3, 5.0 Hz, 1H, H-6b), 4.18 (ddd, *J* = 10.0, 5.0, 3.0 Hz, 1H, H-5), 2.48 (d, *J* = 9.8 Hz, 1H, SH). ^13^C{^1^H} NMR (101 MHz, CDCl_3_): δ 166.3, 165.9, 165.6, 165.3 (4× C=O), 133.6, 133.5, 133.3, 130.0, 123.0, 123.0, 129.9, 129.7, 129.1, 128.9, 128.6, 128.6, 128.5, 128.5 (Aryl C), 79.3 (CH), 76.9 (CH), 74.4 (CH), 74.0 (CH), 69.5 (CH), 63.3 (CH_2_). Spectral data matched literature reports.^18c^

### 4.5 Synthesis of 2,3,4,6-Tetra-*O*-acetyl-1-thio-α-d-glucopyranoside

#### 4.5.1 2,3,4,6-Tetra-*O*-benzoyl-1-thio-α-d-glucopyranose (**23**)

Thiol **22** (1.22 g, 2 mmol) and Cu(OTf)_2_ (0.145 g, 0.40 mmol, 0.2 equiv) were dried under high vacuum before being dissolved in dry CH_2_Cl_2_. The solution was stirred at rt for 5 h, at which time it had become a dark orange color. The solution was filtered through Celite and then concentrated in vacuo. The crude material was purified by column chromatography (cHex/EtOAc 7:1) to afford the title compound as a colorless oil (0.650 g, 1.07 mmol, 53%). ^1^H NMR (400 MHz, CDCl_3_): δ 8.06 (m, 2H, Ar H), 7.99 (m, 2H, Ar H), 7.97–7.93 (m, 2H, Ar H), 7.90–7.86 (m, 2H, Ar H), 7.59–7.49 (m, 3H, Ar H), 7.46–7.42 (m, 3H, Ar H), 7.41–7.34 (m, 4H, Ar H), 7.31 (m, 2H, Ar H), 6.20 (t, *J* = 5.7 Hz, 1H, H-1), 6.08 (t, *J* = 9.9 Hz, 1H, H-3), 5.72 (t, *J* = 9.8 Hz, 1H, H-4), 5.50 (dd, *J* = 10.2, 5.7 Hz, 1H, H-2), 4.87 (ddd, *J* = 10.1, 4.7, 2.8 Hz, 1H, H-5), 4.64 (dd, *J* = 12.4, 2.8 Hz, 1H, H-6a), 4.49 (dd, *J* = 12.3, 4.7 Hz, 1H, H-6b), 2.08 (d, *J* = 5.7 Hz, 1H, SH). Spectral data matched literature reports.^18c^

#### 4.5.2 Monomethoxy Trityl-2,3,4,6-tetra-*O*-benzoyl-1-thio-α-d-glucopyranoside (**24**)

To a solution of thiol **23** (1.50 g, 2.45 mmol) in pyridine (10 mL) was added monomethoxy trityl chloride (0.830 g, 2.69 mmol, 1.1 equiv). The solution was stirred at room temperature for 18 h, at which time TLC (2:1 cHex/EtOAc) indicated the presence of a single product. The reaction mixture was concentrated in vacuo, then diluted with ethyl acetate before washing with 8% dilute aqueous HCl, water, and brine. The organic layer was dried over MgSO_4_, filtered, and concentrated in vacuo. The crude oil was purified by flash chromatography (8:1 → 6:1 cHex/EtOAc) to afford the title compound as a colorless solid (1.78 g, 2.00 mmol, 82% yield). ^1^H NMR (400 MHz, CDCl_3_): δ 8.10–8.04 (m, 2H), 7.99 (dd, *J* = 8.3, 1.4 Hz, 2H), 7.89 (dd, *J* = 8.4, 1.4 Hz, 2H), 7.85–7.80 (m, 2H), 7.59–7.48 (m, 2H), 7.48–7.41 (m, 3H), 7.40–7.34 (m, 7H), 7.32 (d, *J* = 7.8 Hz, 2H), 7.27–7.23 (m, 4H), 7.19–7.09 (m, 6H), 6.69–6.58 (m, 2H) (34 Ar–H), 5.98 (dd, *J* = 10.6, 9.3 Hz, 1H, H-3), 5.69 (t, *J* = 9.6 Hz, 1H, H-4), 5.50 (dd, *J* = 10.6, 5.6 Hz, 1H, H-2), 5.30 (d, *J* = 5.6 Hz, 1H, H-1), 4.77 (d, *J* = 10.0 Hz, 1H, H-5), 4.35 (d, *J* = 3.3 Hz, 2H, H-6a + b), 3.71 (s, 3H, PhOMe). ^13^C{^1^H} NMR (101 MHz, CDCl_3_): δ 166.3, 165.8, 165.2, 165.2 (4× C=O), 158.5 (Ar C–OCH_3_), 144.8, 144.7, 136.3 (3x Ar C–CS), 133.7, 133.5, 133.3, 133.1 (4× benzoyl Ar–CH), 131.2, 130.2, 130.0, 129.9, 129.8, 129.8, 129.8, 129.2, 129.1, 129.1, 128.7, 128.5, 128.5, 128.1, 128.0, 127.1, 113.3 (Ar CH), 83.0 (C-1), 71.3 (C-3), 70.9 (C-2), 69.7 (C-4), 69.6 (C-5), 69.2 (S-C-(Ph)_2_PhOMe), 63.0 (C-6), 55.3 (OCH_3_). HRMS (ESI+/QTOF): *m*/*z* calculated for C_54_H_44_O_10_SNa ([M + Na]^+^), 907.2553; found, 907.2547.

#### 4.5.3 2,3,4,6-Tetra-*O*-acetyl-1-thio-α-d-glucopyranose (**25**)

Compound **24** (1.78 g, 2.00 mmol) was dried under a high vacuum and then dissolved in dry MeOH (30 mL). NaOMe (22 mg, 0.40 mmol, 0.2 equiv) was added, and the mixture was stirred at rt for 3 h, at which time TLC (2:1 cHex/EtOAc) showed a single spot on the baseline. The reaction mixture was neutralized with Amberlite IRA H^+^ resin, filtered, and concentrated in vacuo. The resulting oil was azeotroped with toluene (2×, 5 mL). The crude solid was dissolved in pyridine (20 mL) and acetic anhydride (1.1 mL, 12 mmol, 6 equiv) was added. The solution was stirred at rt for 5 h. The reaction mixture was concentrated in vacuo before diluting with ethyl acetate (100 mL). The organic layer was washed sequentially with 8% aq. HCl, water, and brine. The organic layer was collected and dried over anhydrous MgSO_4_, filtered, and concentrated in vacuo. The crude material was dried under a high vacuum and dissolved in dry CH_2_Cl_2_. The solution was cooled to 0 °C and trifluoroacetic acid (1.5 mL, 20 mmol, 10 equiv) was added dropwise, upon which a bright orange color developed. Et_3_SiH (1.7 mL, 16 mmol, 0.8 equiv) was then added slowly, and the mixture allowed to obtain room temperature. The solution was stirred for 16 h, after which time it became colorless. The mixture was concentrated in vacuo and purified by column chromatography (cHex/EtOAc 5:1 → 3:1) to afford the title compound as a colorless solid (0.652 g, 1.72 mmol, 86%). mp 87–89 °C. ^1^H NMR (400 MHz, CDCl_3_): δ 5.93 (t, *J* = 5.7 Hz, 1H, H-1), 5.38 (dd, *J* = 10.1, 9.5 Hz, 1H, H-3), 5.09–5.00 (m, 2H, H-2/H-4), 4.43 (ddd, *J* = 10.3, 4.3, 2.3 Hz, 1H, H-5), 4.29 (dd, *J* = 12.4, 4.3 Hz, 1H, H-6a), 4.10 (dd, *J* = 12.5, 2.3 Hz, 1H, H-6b), 2.09 (s, 3H), 2.08 (s, 3H), 2.04 (s, 3H), 2.02 (s, 3H) (4× acetyl CH_3_), 1.91 (d, *J* = 5.8 Hz, 1H, SH). ^13^C{^1^H} NMR (101 MHz, CDCl_3_): δ 170.8, 170.1, 169.8, 169.7 (4× C=O), 77.3 (C-1), 70.4 (C-2), 70.0 (C-3), 68.4 (C-4), 68.4 (C-5), 61.8 (C-6), 20.8, 20.8, 20.8, 20.7 (4× acetyl CH_3_). Spectral data matched literature reports.^19b^

### 4.6 Synthesis of Aryl Thioglycosides from Glycosyl Thiols **18–21** and **25**

#### 4.6.1 Phenyl 2,3,4,6-Tetra-*O*-acetyl-1-thio-α-d-glucopyranose (**26**)

Thiol **25** (95 mg, 0.25 mmol) and iodobenzene (84 μL, 0.75 mmol) were treated according to the general procedure and purified by column chromatography (cHex/EtOAc 2:1) to produce the title compound as a colorless solid (68 mg, 0.15 mmol, 62% yield). mp 85–86 °C. ^1^H NMR (500 MHz, CDCl_3_): δ 7.46–7.42 (m, 2H, ArH), 7.33–7.27 (m, 3H, ArH), 5.92 (d, *J* = 5.8 Hz, 1H, H-1), 5.44 (dd, *J* = 10.5, 9.2 Hz, 1H, H-3), 5.12–5.05 (m, 2H, H-2, H-4), 4.57 (ddd, *J* = 10.2, 5.1, 2.2 Hz, 1H, H-5), 4.28 (dd, *J* = 12.3, 5.2 Hz, 1H, H-6a), 4.03 (dd, *J* = 12.3, 2.3 Hz, 1H, H-6b), 2.10 (s, 3H), 2.05 (s, 3H), 2.04 (s, 3H), 2.02 (s, 3H) (4× acetyl CH_3_). ^13^C{^1^H} NMR (126 MHz, CDCl_3_): δ 170.7, 170.1, 170.0, 169.8 (4× C=O), 132.6 (Ar C–S), 132.0, 129.3, 128.0 (3× Ar C–H), 85.1 (C-1), 70.9 (C-2), 70.6 (C-3), 68.72 (C-4), 68.3 (C-5), 62.1 (C-6), 20.9, 20.8, 20.8 (acetyl CH_3_). Spectral data matched literature reports.^17^

#### 4.6.2 *para*-Tolyl 2,3,4,6-Tetra-*O*-acetyl-1-thio-α-d-glucopyranoside (**27**)

Thiol **25** (95 mg, 0.25 mmol) and a solution of 4-iodotoluene (0.164 g, 0.75 mmol) in THF (75 μL, 10 M) were treated according to the general procedure and purified by column chromatography (cHex/EtOAc 2:1) to produce the title compound as a colorless solid (66 mg, 0.15 mmol, 58% yield). mp 93–95 °C. ^1^H NMR (400 MHz, CDCl_3_): δ 7.32 (d, *J* = 8.1 Hz, 2H), 7.11 (dt, *J* = 8.1, 0.7 Hz, 2H), 5.83 (d, *J* = 5.8 Hz, 1H, H-1), 5.43 (dd, *J* = 10.3, 9.3 Hz, 1H, H-3), 5.11–5.04 (m, 2H, H-2, H-4), 4.58 (ddd, *J* = 10.2, 5.0, 2.3 Hz, 1H, H-5), 4.27 (dd, *J* = 12.3, 5.1 Hz, 1H, H-6a), 4.03 (dd, *J* = 12.3, 2.3 Hz, 1H, H-6b), 2.32 (s, 3H, PhCH_3_), 2.10 (s, 3H), 2.05 (s, 3H), 2.03 (2× s, each 3H) (4× acetyl CH_3_). ^13^C{^1^H} NMR (101 MHz, CDCl_3_): δ 170.7, 170.1, 170.0, 169.8 (4× C=O), 138.3 (ArC_*q*_-S), 132.7, 130.1, 130.1 (3× ArCH), 128.7 (ArC_*q*_CH_3_), 85.6 (C-1), 71.0 (C-2), 70.6 (C-3), 68.8 (C-4), 68.2 (C-5), 62.1 (C-6), 21.3, 20.9, 20.8, 20.8 (4× acetyl CH_3_). Spectral data matched literature reports.^17^

#### 4.6.3 (2-Pyridinyl) 2,3,4,6-Tetra-*O*-acetyl-1-thio-α-d-glucopyranoside (**28**)

Thiol **25** (95 mg, 0.25 mmol) and 2-bromopyridine (84 μL, 0.75 mmol) were treated according to the general procedure and purified by column chromatography (cHex/EtOAc 2:1) to produce the title compound as a colorless oil (24 mg, 0.05 mmol, 22% yield). ^1^H NMR (500 MHz, CDCl_3_): δ 8.46 (ddd, *J* = 4.9, 1.9, 0.9 Hz, 1H, pyridine H-6), 7.54 (td, *J* = 7.7, 1.9 Hz, 1H, pyridine H-4), 7.29 (dt, *J* = 8.1, 1.0 Hz, 1H, pyridine H-3), 7.07 (ddd, *J* = 7.5, 4.9, 1.1 Hz, 1H, pyridine H-5), 6.66 (d, *J* = 5.7 Hz, 1H, H-1), 5.40 (dd, *J* = 10.3, 9.3 Hz, 1H, H-3), 5.25 (dd, *J* = 10.3, 5.7 Hz, 1H, H-2), 5.12 (dd, *J* = 10.3, 9.2 Hz, 1H, H-4), 4.37 (ddd, *J* = 10.3, 4.5, 2.3 Hz, 1H, H-5), 4.26 (dd, *J* = 12.4, 4.5 Hz, 1H, H-6a), 4.00 (dd, *J* = 12.4, 2.3 Hz, 1H, H-6b), 2.03 (s, 3H), 2.03 (s, 3H), 2.02 (s, 3H), 1.98 (s, 3H) (4× acetyl CH_3_). ^13^C{^1^H} NMR (126 MHz, CDCl_3_): δ 170.7, 170.1, 169.9, 169.7 (4× C=O), 155.5 (pyridine C-2), 150.0 (pyridine C-6), 136.8 (pyridine C-4), 123.9 (pyridine C-3), 121.1 (pyridine C-5), 81.7 (C-1), 71.1 (C-3), 70.0 (C-2), 69.5 (C-5), 68.5 (C-4), 61.9 (C-6), 20.8, 20.8, 20.8, 20.7 (4× acetyl CH_3_). Spectral data matched literature reports.^17^

#### 4.6.4 *para*-Methoxyphenyl 2,3,4,6-Tetra-*O*-acetyl-1-thio-α-d-glucopyranoside (**29**)

Thiol **25** (95 mg, 0.25 mmol) and 4-bromoanisle (94 μL, 0.75 mmol) were treated according to the general procedure and purified by column chromatography (cHex/EtOAc 2:1) to produce the title compound as a colorless oil (42 mg, 0.09 mmol, 36% yield). ^1^H NMR (400 MHz, CDCl_3_): δ 7.36 (d, *J* = 8.9 Hz, 2H, ArH), 6.83 (d, *J* = 8.9 Hz, 2H, ArH), 5.74 (d, *J* = 5.7 Hz, 1H, H-1), 5.43 (dd, *J* = 10.5, 9.2 Hz, 1H, H-3), 5.08–5.04 (m, 2H, H-2/H-4), 4.60 (ddd, *J* = 10.3, 5.1, 2.3 Hz, 1H, H-5), 4.27 (dd, *J* = 12.3, 5.1 Hz, 1H, H-6a), 4.04 (dd, *J* = 12.3, 2.3 Hz, 1H, H-6b), 3.78 (s, 3H, OCH_3_), 2.11 (s, 3H), 2.05 (s, 3H), 2.04 (s, 3H), 2.03 (s, 3H) (4× acetyl CH_3_). ^13^C{^1^H} NMR (101 MHz, CDCl_3_): δ 170.7, 170.1, 170.0, 169.8 (4× C=O), 160.1 (ArC_q_-OMe), 135.1 (ArCH), 122.5 (ArC_q_-S), 114.9 (ArCH), 86.2 (C-1), 71.0 (C-2), 70.6 (C-3), 68.8 (C-4), 68.1 (C-5), 62.2 (C-6), 55.5 (OCH_3_), 20.9, 20.8, 20.8, 20.8 (4× acetyl CH_3_). Spectral data matched literature reports.^17^

#### 4.6.5 *para*-Hydroxyphenyl 2,3,4,6-Tetra-*O*-acetyl-1-thio-α-d-glucopyranoside (**30**)

Thiol **25** (95 mg, 0.25 mmol) and a solution of 4-iodophenol (0.165 g, 0.75 mmol) in THF (75 μL, 10 M) were treated according to the general procedure and purified by column chromatography (cHex/EtOAc 2:1) to produce the title compound as a colorless oil (45 mg, 0.10 mmol, 40% yield). ^1^H NMR (500 MHz, CDCl_3_): δ 7.29 (d, *J* = 8.6 Hz, 2H, ArH), 6.76 (d, *J* = 8.6 Hz, 2H, ArH), 5.73 (d, *J* = 5.7 Hz, 1H, H-1), 5.42 (dd, *J* = 10.3, 9.3 Hz, 1H, H-3), 5.09–5.03 (m, 2H, H-2 + H-4), 4.60 (ddd, *J* = 10.3, 5.0, 2.3 Hz, 1H, H-5), 4.27 (dd, *J* = 12.3, 5.0 Hz, 1H, H-6a), 4.05 (dd, *J* = 12.3, 2.3 Hz, 1H, H-6b), 2.11 (s, 3H, acetyl CH_3_), 2.05 (s, 6H, 2× acetyl CH_3_), 2.03 (s, 3H, acetyl CH_3_). ^13^C{^1^H} NMR (126 MHz, CDCl_3_): δ 171.1, 170.3, 170.3, 169.9 (4× C=O), 156.7 (Ar C–OH), 135.4 (Ar CH), 122.2 (Ar C–S), 116.5 (Ar CH), 86.2 (C-1), 71.1 (C-2), 70.6 (C-3), 68.8 (C-4), 68.0 (C-5), 62.2 (C-6), 20.9, 20.8, 20.8, 20.8 (4× acetyl CH_3_). HRMS (ESI+) calcd for C_20_H_28_NO_10_SNa [M + NH_4_]^+^, 474.1428; found, 474.1429.

#### 4.6.6 Phenyl 2,3,4,6-Tetra-*O*-acetyl-1-thio-α-d-mannopyranoside (**31**)

Thiol **20** (95 mg, 0.25 mmol) and iodobenzene (84 μL, 0.75 mmol) were treated according to the general procedure and purified by column chromatography (cHex/EtOAc 2:1) to produce the title compound as a colorless oil (75 mg, 0.17 mmol, 68% yield). ^1^H NMR (400 MHz, CDCl_3_): δ 7.51–7.45 (m, 2H, ArH), 7.34–7.27 (m, 3H, ArH), 5.53–5.45 (m, 2H, H-1/H-2), 5.35–5.28 (m, 2H, H-3/H-4), 4.57–4.48 (m, 1H, H-5), 4.29 (dd, *J* = 12.2, 5.9 Hz, 1H, H-6a), 4.09 (dd, *J* = 12.3, 2.4 Hz, 1H, H-6b), 2.14 (s, 3H), 2.06 (s, 3H), 2.04 (s, 3H), 2.01 (s, 3H) (4× acetyl CH_3_). ^13^C{^1^H} NMR (101 MHz, CDCl_3_): δ 170.6, 170.0, 169.9, 169.8 (4× C=O), 132.7 (ArC_q_), 132.2, 129.3, 128.2 (3× ArCH), 85.8 (C-1), 71.0 (C-2), 69.6 (C-5), 69.5 (C3/C4), 66.5 (C3/C4), 62.5 (C-6), 21.0, 20.8, 20.8, 20.7 (4× acetyl CH_3_). Spectral data matched literature reports.^16^

#### 4.6.7 *para*-Methylphenyl 2,3,4,6-Tetra-*O*-acetyl-1-thio-α-d-mannopyranoside (**32**)

Thiol **20** (95 mg, 0.25 mmol) and a solution of 4-iodotoluene (0.164 g, 0.75 mmol) in THF (75 μL, 10 M) were treated according to the general procedure and purified by column chromatography (cHex/EtOAc 2:1) to produce the title compound as a colorless oil (69 mg, 15 mmol, 61% yield). ^1^H NMR (400 MHz, CDCl_3_): δ 7.36 (d, *J* = 8.1 Hz, 2H, ArH), 7.14–7.06 (m, 2H, ArH), 5.50–5.44 (m, 1H, H-2), 5.40 (dd, *J* = 1.7, 0.6 Hz, 1H, H-1), 5.34–5.27 (m, 2H, H-3/H-4), 4.58–4.49 (m, 1H, H-5), 4.28 (dd, *J* = 12.2, 5.9 Hz, 1H, H-6a), 4.09 (dd, *J* = 12.2, 2.4 Hz, 1H, H-6b), 2.31 (s, 3H, PhCH_3_), 2.13 (s, 3H), 2.06 (s, 3H), 2.04 (s, 3H), 2.00 (s, 3H) (4× acetyl CH_3_). ^13^C{^1^H} NMR (101 MHz, CDCl_3_): δ 170.6, 167.0, 169.9, 169.8 (4× C=O), 138.5 (ArC_q_), 132.7 (ArCH), 130.0 (ArCH), 128.9 (ArC_q_), 86.1 (C-1), 71.0 (C-2), 69.5 (C-5), 69.5 (C-3/C-4), 66.5 (C-3/C-4), 62.6 (C-6), 21.2 (PhCH_3_), 20.9, 20.8, 20.8, 20.7 (4× acetyl CH_3_). Spectral data matched literature reports.^16^

#### 4.6.8 Phenyl 2,3,4,6-Tetra-*O*-acetyl-1-thio-β-d-galactopyranoside (**33**)

Thiol **18** (95 mg, 0.25 mmol) and iodobenzene (84 μL, 0.75 mmol) were treated according to the general procedure and purified by column chromatography (cHex/EtOAc 2:1) to produce the title compound as a colorless solid (0.77 g, 0.17 mmol, 70% yield). ^1^H NMR (400 MHz, CDCl_3_): δ 7.53–7.47 (m, 2H, ArH), 7.33–7.29 (m, 3H, ArH), 5.41 (dd, *J* = 3.4, 1.1 Hz, 1H, H-4), 5.23 (t, *J* = 10.0 Hz, 1H, H-3), 5.04 (dd, *J* = 9.9, 3.3 Hz, 1H, H-2), 4.71 (d, *J* = 10.0 Hz, 1H, H-1), 4.18 (dd, *J* = 11.4, 7.0 Hz, 1H, H-6a), 4.11 (dd, *J* = 11.4, 6.1 Hz, 1H, H-6b), 3.93 (ddd, *J* = 7.2, 6.2, 1.1 Hz, 1H, H-5), 2.11 (s, 3H), 2.09 (s, 3H), 2.03 (s, 3H), 1.97 (s, 3H)(4× acetyl CH_3_). ^13^C{^1^H} NMR (101 MHz, cdcl_3_): δ 170.5, 170.3, 170.2, 169.5 (4× C=O), 132.7 (Ar CH), 132.6 (Ar C–S), 129.0 (Ar CH), 128.3 (ArCH), 86.7 (C-1), 74.5 (C-5), 72.1 (C-2), 67.4, 67.3 (C-4, C-3), 61.7 (C-6), 21.0, 20.8, 20.7, 20.7 (4× acetyl CH_3_). Spectral data matched literature reports.^23^

#### 4.6.9 *para*-Methylphenyl 2,3,4,6-Tetra-*O*-acetyl-1-thio-β-d-galactopyranoside (**34**)

Thiol **18** (95 mg, 0.25 mmol) and a solution of 4-iodotoluene (0.164 g, 0.75 mmol) in THF (75 μL, 10 M) were treated according to the general procedure and purified by column chromatography (cHex/EtOAc 2:1) to produce the title compound as a colorless solid (80 mg, 0.18 mmol, 71% yield). mp 111–113 °C. ^1^H NMR (400 MHz, CDCl_3_): δ 7.41 (d, *J* = 8.1 Hz, 2H, ArH), 7.12 (dt, *J* = 8.0, 0.7 Hz, 2H, ArH), 5.40 (dd, *J* = 3.4, 1.1 Hz, 1H, H-4), 5.21 (t, *J* = 10.0 Hz, 1H, H-2), 5.03 (dd, *J* = 10.0, 3.4 Hz, 1H, H-3), 4.64 (d, *J* = 10.0 Hz, 1H, H-1), 4.18 (dd, *J* = 11.3, 6.9 Hz, 1H, H-6a), 4.10 (dd, *J* = 11.3, 6.3 Hz, 1H, H-6b), 3.90 (ddd, *J* = 7.0, 6.4, 1.1 Hz, 1H, H-5), 2.34 (s, 3H, PhCH_3_), 2.11 (s, 3H), 2.09 (s, 3H), 2.04 (s, 3H), 1.97 (s, 3H)(4× acetyl CH_3_). ^13^C{^1^H} NMR (101 MHz, CDCl_3_): δ 170.5, 170.3, 170.2, 169.6 (4× C=O), 138.6 (ArC_*q*_CH_3_), 133.3(ArCH), 129.8 (ArCH), 128.8 (ArC_*q*_S), 87.1 (C-1), 74.5 (C-5), 72.2 (C-3), 67.4 (C-2), 67.4 (C-4), 61.7 (C-6), 21.3 (PhCH_3_), 21.0, 20.8, 20.8, 20.7 (4× acetyl CH_3_). Spectral data matched literature reports.^16^

#### 4.6.10 *para*-Methoxyphenyl 2,3,4,6-Tetra-*O*-acetyl-1-thio-β-d-galactopyranoside (**35**)

Thiol **18** (95 mg, 0.25 mmol) and 4-bromoanisle (94 μL, 0.75 mmol) were treated according to the general procedure and purified by column chromatography (cHex/EtOAc 2:1) to produce the title compound as a colorless oil (67 mg, 0.14 mmol, 57% yield).^1^H NMR (400 MHz, CDCl_3_): δ 7.49–7.42 (m, 2H, ArH), 6.88–6.81 (m, 2H, ArH), 5.37 (dd, *J* = 3.4, 1.1 Hz, 1H, H-4), 5.16 (t, *J* = 9.9 Hz, 1H, H-2), 5.01 (dd, *J* = 9.9, 3.3 Hz, 1H, H-3), 4.55 (d, *J* = 10.0 Hz, 1H, H-1), 4.16 (dd, *J* = 11.3, 6.8 Hz, 1H, H-6a), 4.08 (dd, *J* = 11.2, 6.4 Hz, 1H, H-6b), 3.87 (td, *J* = 6.6, 1.1 Hz, 1H, h-5), 3.79 (s, 3H, PhOCH_3_), 2.10 (s, 3H), 2.08 (s, 3H), 2.02 (s, 3H), 1.95 (s, 3H) (4× acetyl CH_3_). ^13^C{^1^H} NMR (101 MHz, CDCl_3_): δ 170.5, 170.3, 170.2, 169.5 (4× C=O), 160.4 (ArC_q_-OMe), 136.0 (ArCH), 122.1 (ArC_q_-S), 114.5 (ArCH), 87.1 (C-1), 74.4 (C-5), 72.2 (C-3), 67.4 (C-2), 67.3 (C-4), 61.6 (C-6), 55.4 (OCH_3_), 21.0, 20.8, 20.7, 20.7 (4× acetyl CH_3_). Spectral data matches literature reports.^22a^

#### 4.6.11 (5-Indolyl) 2,3,4,6-Tetra-*O*-acetyl-1-thio-β-d-galactopyranoside (**36**)

Thiol **18** (95 mg, 0.25 mmol) and a solution of 5-bromoindole (0.147 g, 0.75 mmol) in THF (75 μL, 10 M) were treated according to the general procedure and purified by column chromatography (cHex/EtOAc 2:1) to produce the title compound as a colorless oil (46 mg, 0.10 mmol, 39% yield).^1^H NMR (500 MHz, CDCl_3_): δ 8.42 (s, 1H, N–H), 7.86 (dt, *J* = 1.5, 0.8 Hz, 1H, Indole H-4), 7.37–7.31 (m, 2H, Indole H-6 + H-7), 7.23 (dd, *J* = 3.2, 2.4 Hz, 1H, Indole H-2), 6.52 (td, *J* = 2.1, 1.0 Hz, 1H, Indole H-3), 5.38 (dd, *J* = 3.3, 1.1 Hz, 1H, H-4), 5.23 (t, *J* = 10.0 Hz, 1H, H-2), 5.02 (dd, *J* = 10.0, 3.4 Hz, 1H, H-3), 4.64 (d, *J* = 10.1 Hz, 1H, H-1), 4.19 (dd, *J* = 11.3, 6.8 Hz, 1H, H-6a), 4.10 (dd, *J* = 11.3, 6.4 Hz, 1H, H-6b), 3.85 (td, *J* = 6.6, 1.1 Hz, 1H, H-5), 2.13 (s, 3H), 2.06 (s, 3H), 2.00 (s, 3H), 1.96 (s, 3H) (acetyl CH_3_). ^13^C{^1^H} NMR (126 MHz, CDCl_3_): δ 170.6, 170.4, 170.3, 169.7 (4× C=O), 136.0 (indole C-8), 128.6 (indole C-9), 128.1 (indole C-6), 127.3 (indole C-4), 125.4 (indole C-2), 121.8 (indole C-5), 111.5 (indole C-7), 102.8 (indole C-3), 88.2 (C-1), 74.4 (C-5), 72.2 (C-3), 67.6 (C-2), 67.4 (C-4), 61.7 (C-6), 21.1, 20.8, 20.7, 20.7 (4× acetyl CH_3_). HRMS (ESI+/QTOF) *m*/*z* calcd for C_22_H_26_NO_9_S [M + H]^+^, 480.1328; found, 480.1323.

#### 4.6.12 Phenyl 2,3,4-Tri-*O*-acetyl-1-thio-β-d-fucopyranoside (**37**)

Thiol **19** (76 mg, 0.25 mmol) and iodobenzene (84 μL, 0.75 mmol) were treated according to the general procedure and purified by column chromatography (cHex/EtOAc 2:1) to produce the title compound as a colorless oil (57 mg, 0.15 mmol, 60% yield).^1^ H NMR (400 MHz, CDCl_3_): δ 7.50 (dd, *J* = 7.3, 2.4 Hz, 2H, Ar H), 7.35–7.27 (m, 3H, Ar H), 5.25 (dd, *J* = 3.4, 1.1 Hz, 1H, H-4), 5.22 (t, *J* = 9.9 Hz, 1H, H-2), 5.05 (dd, *J* = 9.9, 3.4 Hz, 1H, H-3), 4.70 (d, *J* = 9.9 Hz, 1H, H-1), 3.83 (qd, *J* = 6.4, 1.1 Hz, 1H, H-5), 2.14 (s, 3H), 2.07 (s, 3H), 1.96 (s, 3H) (3× acetyl CH_3_), 1.23 (d, *J* = 6.4 Hz, 3H, H-6). ^13^C{^1^H} NMR (101 MHz, CDCl_3_): δ 170.7, 170.2, 169.6 (3× C=O), 132.98 (Ar C–S), 132.4, 129.0, 128.0 (3× Ar CH), 86.5 (C-1), 73.2 (C-5), 72.5 (C-3), 70.4 (C-4), 67.5 (C-2), 20.9, 20.8, 20.7 (3× acetyl CH_3_), 16.6 (C-6). Spectral data matches literature reports.^44^

#### 4.6.13 *para*-Methylphenyl 2,3,4-Tri-*O*-acetyl-1-thio-β-d-fucopyranoside (**38**)

Thiol **19** (76 mg, 0.25 mmol) and a solution of 4-iodotoluene (0.164 g, 0.75 mmol) in THF (75 μL, 10 M) were treated according to the general procedure and purified by column chromatography (cHex/EtOAc 2:1) to produce the title compound as a colorless solid (75 mg, 0.19 mmol, 76% yield). ^1^H NMR (400 MHz, CDCl_3_): δ 7.40 (d, *J* = 8.1 Hz, 2H, Ar H), 7.12 (d, *J* = 7.8 Hz, 2H, Ar H), 5.24 (dd, *J* = 3.4, 1.0 Hz, 1H, H-4), 5.19 (t, *J* = 9.9 Hz, 1H, H-2), 5.03 (dd, *J* = 9.9, 3.4 Hz, 1H, H-3), 4.63 (d, *J* = 9.9 Hz, 1H, H-1), 3.79 (qd, *J* = 6.4, 1.1 Hz, 1H, H-5), 2.33 (s, 3H), 2.13 (s, 3H), 2.07 (s, 3H), 1.96 (s, 3H) (3x acetyl CH_3_), 1.22 (d, *J* = 6.4 Hz, 3H, H-6). ^13^C{^1^H} NMR (101 MHz, CDCl_3_): δ 170.7, 170.2, 169.6 (3× C=O), 138.3 (Ar C–CH_3_), 133.0 (Ar CH), 129.7 (Ar CH), 129.2 (Ar C–S), 86.9 (C-1), 73.2 (C-5), 72.6 (C-3), 70.5 (C-4), 67.5 (C-2), 21.3 (tolyl CH_3_), 21.0, 20.8, 20.7 (3× acetyl CH_3_), 16.6 (C-6). Spectral data matched literature reports.^45^

#### 4.6.14 *para*-Methoxyphenyl 2,3,4-Tri-*O*-acetyl-1-thio-β-d-fucopyranoside (**39**)

Thiol **19** (76 mg, 0.25 mmol) and 4-bromoanisle (94 μL, 0.75 mmol) were treated according to the general procedure and purified by column chromatography (cHex/EtOAc 2:1) to produce the title compound as a colorless solid (62 mg, 0.15 mmol, 60% yield). ^1^H NMR (400 MHz, CDCl_3_): δ 7.45 (d, *J* = 8.8 Hz, 2H), 6.83 (d, *J* = 8.8 Hz, 2H), 5.21 (dd, *J* = 3.3, 1.1 Hz, 1H, H-4), 5.13 (t, *J* = 9.9 Hz, 1H, H-2), 5.00 (dd, *J* = 10.0, 3.3 Hz, 1H, H-3), 4.53 (d, *J* = 9.9 Hz, 1H, H-1), 3.80–3.72 (m, 4H, H-5 + OCH_3_), 2.10 (s, 3H), 2.08 (s, 3H), 1.94 (s, 3H) (3× acetyl CH_3_), 1.19 (d, *J* = 6.4 Hz, 3H, CH_3_-6). ^13^C{^1^H} NMR (101 MHz, CDCl_3_): δ 170.7, 170.2, 169.7(3× C=O), 160.2 (Ar C–O), 135.7 (Ar CH), 122.6 (Ar C–S), 114.4 (Ar C–H), 87.0 (C-1), 73.1 (C-5), 72.6 (C-3), 70.4 (C-4), 67.5 (C-2), 55.4 (OCH_3_), 21.0, 20.7, 20.7 (3× acetyl CH_3_), 16.5 (C-6). HRMS (ESI+/QTOF): *m*/*z* calcd for C_19_H_24_O_8_ SNa ([M + Na]^+^), 435.1090; found, 435.1083.

#### 4.6.15 (5-Indolyl) 2,3,4-Tri-*O*-acetyl-1-thio-β-d-fucopyranoside (**40**)

Thiol **19** (76 mg, 0.25 mmol) and a solution of 5-bromoindole (0.147 g, 0.75 mmol) in THF (75 μL, 10 M) were treated according to the general procedure and purified by column chromatography (cHex/EtOAc 2:1) to produce the title compound as a colorless solid (43 mg, 0.10 mmol, 41% yield). ^1^H NMR (400 MHz, CDCl_3_): δ 8.39 (s, 1H), 7.87 (dt, *J* = 1.6, 0.8 Hz, 1H), 7.39–7.31 (m, 2H), 7.22 (dd, *J* = 3.2, 2.4 Hz, 1H), 6.53 (ddd, *J* = 3.2, 2.0, 0.8 Hz, 1H), 5.25–5.17 (m, 2H, H-2 + H-4), 5.03 (dd, *J* = 9.9, 3.4 Hz, 1H, H-3), 4.63 (d, *J* = 10.0 Hz, 1H, H-1), 3.73 (qd, *J* = 6.4, 1.1 Hz, 1H, H-5), 2.12 (s, 3H), 2.09 (s, 3H), 1.96 (s, 3H) (3× acetyl CH_3_), 1.21 (d, *J* = 6.4 Hz, 3H, H-6). ^13^C{^1^H} NMR (101 MHz, CDCl_3_): δ 170.9, 170.3, 169.8 (3× C=O), 135.9 (indole C-8), 128.6 (indole C-9), 128.0 (indole C-6), 127.1 (indole C-4), 125.3 (indole C-2), 122.4 (indole C-5), 111.5 (indole C-7), 102.8 (indole C-3), 88.3 (C-1), 73.2 (C-5), 72.7 (C-3), 70.6 (C-4), 67.8 (C-2), 21.1, 20.8, 20.8 (3× acetyl CH_3_), 16.6 (C-6). HRMS (ESI+/QTOF): *m*/*z* calcd for C_20_H_23_NO_7_SNa ([M + Na]^+^), 444.1092; found, 444.1086.

#### 4.6.16 Phenyl 2,3,4,6-Tetra-*O*-acetyl-β-d-galactopyranosyl-(1 → 4)-2,3,6-tri-*O*-acetyl-1-thio-β-d-glucopyranoside (**41**)

Thiol **21** (0.167 g, 0.25 mmol) and iodobenzene (84 μL, 0.75 mmol) were treated according to the general procedure and purified by column chromatography (cHex/EtOAc 1:1) to produce the title compound as a colorless solid (0.121 g, 0.17 mmol, 66% yield). mp 158–161 °C. ^1^H NMR (400 MHz, CDCl_3_): δ 7.48–7.43 (m, 2H, ArH), 7.32–7.27 (m, 3H, ArH), 5.32 (d, *J* = 3.4 Hz, 1H, H-4), 5.20 (t, *J* = 9.1 Hz, 1H, H-3′), 5.08 (dd, *J* = 10.2, 7.9 Hz, 1H, H-2), 4.93 (dd, *J* = 10.4, 3.4 Hz, 1H, H-3), 4.88 (t, *J* = 9.5 Hz, 1H, H-2′), 4.66 (d, *J* = 10.1 Hz, 1H, H-1′), 4.51 (d, *J* = 11.1 Hz, 1H, H-6a), 4.46 (d, *J* = 7.9 Hz, 1H, H-1), 4.13–4.00 (m, 3H, H-6b/H-6a′/H-6b′), 3.85 (t, *J* = 6.1 Hz, 1H, H-5), 3.73 (t, *J* = 9.5 Hz, 1H, H-4′), 3.62 (dd, *J* = 10.1, 3.3 Hz, 1H, H-5′), 2.12 (s, 3H), 2.08 (s, 3H), 2.07 (s, 3H), 2.02 (m, 9H), 1.94 (s, 3H) (7× acetyl CH_3_). ^13^C{^1^H} NMR (101 MHz, CDCl_3_): δ 170.4, 170.4, 170.2, 170.1, 169.8, 169.7, 169.1 (7× C=O), 133.1 (ArCH), 131.9 (ArC_q_-S), 129.0 (ArCH), 128.4 (ArCH), 101.1 (C-1), 85.6 (C-1′), 76.8 (C-5′), 76.2 (C-4′), 74.0 (C-3′), 71.1 (C-3), 70.8 (C-5), 70.4 (C-2′), 69.2 (C-2), 66.7 (C-4), 62.2 (C-6), 60.9 (C-6′), 20.92, 20.9, 20.7, 20.7, 20.6 (acetyl CH_3_). Spectral data matched literature reports.^16^

#### 4.6.17 *para*-Methylphenyl 2,3,4,6-Tetra-*O*-acetyl-β-d-galactopyranosyl-(1 → 4)-2,3,6-tri-*O*-acetyl-1-thio-β-d-glucopyranoside (**42**)

Thiol **21** (0.167 g, 0.25 mmol) and a solution of 4-iodotoluene (0.164 g, 0.75 mmol) in THF (75 μL, 10 M) were treated according to the general procedure and purified by column chromatography (cHex/EtOAc 1:1) to produce the title compound as a colorless solid (61 mg, 0.08 mmol, 33% yield). mp 150–152 °C. ^1^H NMR (400 MHz, CDCl_3_): δ 7.36 (d, *J* = 8.1 Hz, 2H, ArH), 7.14–7.07 (m, 2H, ArH), 5.33 (dd, *J* = 3.5, 1.2 Hz, 1H, H-4), 5.19 (t, *J* = 9.2 Hz, 1H, H-3′), 5.08 (dd, *J* = 10.4, 7.9 Hz, 1H, H-2), 4.93 (dd, *J* = 10.4, 3.4 Hz, 1H, H-3), 4.85 (dd, *J* = 10.1, 9.2 Hz, 1H, H-2′), 4.59 (d, *J* = 10.1 Hz, 1H, H-1′), 4.51 (dd, *J* = 11.9, 2.1 Hz, 1H, H-6a), 4.45 (d, *J* = 7.8 Hz, 1H, H-1), 4.15–4.02 (m, 3H, H-6b/H6′a + b), 3.85 (ddd, *J* = 7.5, 6.3, 1.2 Hz, 1H, H-5), 3.72 (dd, *J* = 9.9, 9.1 Hz, 1H, H-4′), 3.60 (ddd, *J* = 10.0, 5.5, 2.1 Hz, 1H, H-5′), 2.33 (s, 3H, PhCH_3_), 2.13 (s, 3H), 2.10 (s, 3H), 2.08 (s, 3H), 2.03 (s, 3H), 2.02 (s, 3H), 2.01 (s, 3H), 1.95 (s, 3H) (7× acetyl CH_3_). ^13^C{^1^H} NMR (101 MHz, CDCl_3_): δ 170.4, 170.4, 170.3, 170.2, 169.8, 169.7, 169.1 (7× C=O), 138.8 (ArC_q_-CH_3_), 133.9 (ArCH), 129.8 (ArCH), 127.8 (ArC_q_-S), 101.1 (C-1), 85.8 (C-1′), 76.8 (C-5′), 76.2 (C-4′), 74.0 (C-3′), 71.1 (C-3), 70.8 (C-5), 70.4 (C-2′), 69.2 (C-2), 66.7 (C-4), 62.2 (C-6), 60.9 (C-6′), 21.3 (PhCH_3_), 21.0, 20.9, 20.9, 20.8, 20.7, 20.6 (acetyl CH_3_). Spectral data matched literature reports.^46^

#### 4.6.18 *para*-Methoxyphenyl 2,3,4,6-Tetra-*O*-acetyl-β-d-galactopyranosyl-(1 → 4)-2,3,6-tri-*O*-acetyl-1-thio-β-d-glucopyranoside (**43**)

Thiol **21** (0.167 g, 0.25 mmol) and 4-bromoanisle (94 μL, 0.75 mmol) were treated according to the general procedure and purified by column chromatography (cHex/EtOAc 1:1) to produce the title compound as a colorless solid (0.119 g, 0.16 mmol, 63% yield).^1^H NMR (400 MHz, CDCl_3_): δ 7.43–7.36 (m, 2H, ArH), 6.81 (d, *J* = 8.8 Hz, 2H, ArH), 5.31 (d, *J* = 3.5 Hz, 1H, H-4), 5.16 (t, *J* = 9.2 Hz, 1H, H-3′), 5.06 (dd, *J* = 10.3, 7.9 Hz, 1H, H-2), 4.91 (dd, *J* = 10.4, 3.4 Hz, 1H, H-3), 4.78 (t, *J* = 9.6 Hz, 1H, H-2′), 4.55–4.45 (m, 2H, H-6a/H-1′), 4.44 (d, *J* = 7.9 Hz, 1H, H-1), 4.12–3.99 (m, 3H, H-6b/H-6′a^+^b^+^), 3.83 (ddd, *J* = 7.5, 6.3, 1.2 Hz, 1H, H-5), 3.78 (s, 3H, PhOCH_3_), 3.68 (t, *J* = 9.4 Hz, 1H, H-4′), 3.56 (ddd, *J* = 10.0, 5.2, 2.0 Hz, 1H, H-5′), 2.11 (s, 3H), 2.07 (d, *J* = 1.2 Hz, 6H), 2.03–1.97 (m, 9H), 1.93 (s, 3H). ^13^C{^1^H} NMR (101 MHz, CDCl_3_): δ 170.4, 170.3, 170.2, 170.1, 169.8, 169.6, 169.1 (7× C=O), 160.4 (Ar C–OMe), 136.6 (Ar CH), 121.1 (Ar C–S), 114.4 (Ar CH), 101.1 (C-1), 85.5 (C-1′), 76.7 (C-5), 76.1 (C-5′), 74.0 (C-4′), 71.0 (C-3′), 70.7 (C-3), 70.3 (C-2′), 69.2 (C-2), 66.67 (C-4), 62.0 (C-6′), 60.8 (C-6), 55.4 (OCH_3_), 20.9, 20.9, 20.9, 20.7, 20.7, 20.6 (acetyl CH_3_). Spectral data matched literature reports.^22a^

## Data Availability

The data underlying this study are available in the published article and its Supporting Information.

## Supporting Information Available

Copies of ^1^H and ^13^C NMR spectra of compounds **3**–**19**, **21**, **24**, and **26**–**43**; copies of ^1^H NMR spectra of compounds **20**, **22**, **23**, and **25**; procedure and microanalysis data for *bis*-phenanthrolino nickel(II) bromide; and data showing correlation between aryl halide substituent Hammett value and product yield (PDF)
