# Supplementary material for: Mild and Effective Method for the Nickel-Catalyzed Arylation of Glycosyl Thiols in Aqueous Surfactant Solution
Source: J Org Chem. 2024 Nov 12;89(23):17502–17. doi: 10.1021/acs.joc.4c02233 (PMC11629379; doi:10.1021/acs.joc.4c02233)
Supplement: Supplementary file 1 — jo4c02233_si_001.pdf [file jo4c02233_si_001.pdf]

Supporting Information

A Mild and Effective Method for the Nickel-Catalysed Arylation of Glycosyl Thiols in Aqueous Surfactant Solution

Zoe Beato,[†,‡] Sally Howard Ihle,[†] and Xiangming Zhu*,[†,‡]

[†] *Centre for Synthesis and Chemical Biology, UCD School of Chemistry, University College Dublin, Belfield, Dublin 4, Ireland*

[‡] *BiOrbic, Bioeconomy SFI Research Centre, University College Dublin, Belfield, Dublin 4, Ireland*

Tel: +353 17162386; Fax: +353 17162501; e-mail: Xiangming.Zhu@ucd.ie

Table of Contents

Preparation of *Bis*-Phenanthroline Nickel (II) Bromide S3
Correlation of Aryl Substituent with Yield S4
^{1}H and ^{13}C{^{1}H} spectra of compound **3** S5
^{1}H and ^{13}C{^{1}H} spectra of compound **4** S6
^{1}H and ^{13}C{^{1}H} spectra of compound **5** S7
^{1}H and ^{13}C{^{1}H} spectra of compound **6** S8
^{1}H and ^{13}C{^{1}H} spectra of compound **7** S9
^{1}H and ^{13}C{^{1}H} spectra of compound **8** S10
^{1}H and ^{13}C{^{1}H} spectra of compound **9** S11
^{1}H and ^{13}C{^{1}H} spectra of compound **10** S12
^{1}H and ^{13}C{^{1}H} spectra of compound **11** S13
^{1}H and ^{13}C{^{1}H} spectra of compound **12** S14
^{1}H and ^{13}C{^{1}H} spectra of compound **13** S15
^{1}H and ^{13}C{^{1}H} spectra of compound **14** S16
^{1}H and ^{13}C{^{1}H} spectra of compound **15** S17
^{1}H and ^{13}C{^{1}H} spectra of compound **16** S18

^{1}H and $^{13}C\{^{1}H\}$ spectra of compound **17** S19
^{1}H and $^{13}C\{^{1}H\}$ spectra of compound **18** S20
^{1}H and $^{13}C\{^{1}H\}$ spectra of compound **19** S21
^{1}H NMR spectrum of compound **20** S22
^{1}H and $^{13}C\{^{1}H\}$ spectra of compound **21** S23
^{1}H and $^{13}C\{^{1}H\}$ spectra of compound **22** S24
^{1}H NMR spectrum of compound **23** S25
^{1}H and $^{13}C\{^{1}H\}$ spectra of compound **24** S26
^{1}H and $^{13}C\{^{1}H\}$ spectra of compound **25** S27
^{1}H and $^{13}C\{^{1}H\}$ spectra of compound **26** S28
^{1}H and $^{13}C\{^{1}H\}$ spectra of compound **27** S29
^{1}H and $^{13}C\{^{1}H\}$ spectra of compound **28** S30
^{1}H and $^{13}C\{^{1}H\}$ spectra of compound **29** S31
^{1}H and $^{13}C\{^{1}H\}$ spectra of compound **30** S32
^{1}H and $^{13}C\{^{1}H\}$ spectra of compound **31** S33
^{1}H and $^{13}C\{^{1}H\}$ spectra of compound **32** S34
^{1}H and $^{13}C\{^{1}H\}$ spectra of compound **33** S35
^{1}H and $^{13}C\{^{1}H\}$ spectra of compound **34** S36
^{1}H and $^{13}C\{^{1}H\}$ spectra of compound **35** S37
^{1}H and $^{13}C\{^{1}H\}$ spectra of compound **36** S38
^{1}H and $^{13}C\{^{1}H\}$ spectra of compound **37** S39
^{1}H and $^{13}C\{^{1}H\}$ spectra of compound **38** S40
^{1}H and $^{13}C\{^{1}H\}$ spectra of compound **39** S41
^{1}H and $^{13}C\{^{1}H\}$ spectra of compound **40** S42
^{1}H and $^{13}C\{^{1}H\}$ spectra of compound **41** S43
^{1}H and $^{13}C\{^{1}H\}$ spectra of compound **42** S44
^{1}H and $^{13}C\{^{1}H\}$ spectra of compound **43** S45
References S46

Preparation of Bis-Phenanthroline Nickel (II) Bromide[1]

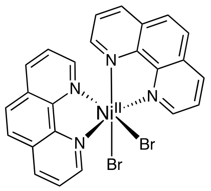

Nickel (II) bromide (1.09 g, 5.00 mmol) and 1,10-phenanthroline (1.80 g, 10.0 mmol) were stirred in acetonitrile (20 mL) at 50 °C for 24 h. The light green suspension was filtered *in vacuo* and the solid was washed with EtOAc (10 mL) followed by pentane (2x 10 mL). The remaining solid was dried overnight. The crude material was recrystallised from hot methanol with slow infusion of EtOAc to achieve the deep green crystalline product (1.60 g, 55% yield). Microanalysis calc. for C24H16Br2N4Ni: C 49.79, H 2.79, N 9.68, Br 27.60 %; Found: C 49.65, H 2.59, N 9.59, Br 27.29 %.

Correlation of Aryl Substituent with Yield

Upon our initial substrate expansion of β-glucosyl thiol **3** with substituted phenyl rings (aryl thioglycoside products **4-12**), we noted a correlation between supposed electron richness of the aryl ring and the yield of thioglycoside obtained. When we compared this quantitatively by plotting yield against the Hammett parameter[2] assigned to each substituent, a trend appeared: in the case that the aryl halide was a liquid, a strong negative correlation between yield and Hammett parameter existed ($R^2 = 0.97$). (Figure S1) In the case of solid aryl halides, a similar negative trend was observed but the correlation was weaker ($R^2 = 0.71$). As a result, we postulated that there was an additional limiting factor when solid aryl halides were used, as solubility and emulsion of the reaction mixture already appeared to be important to reaction outcome. Thus, we repeated the experiments with the solid aryl halides, adding them as solutions (10 M in THF). Upon this, significant increases in yield were observed as outlined in Table S1.

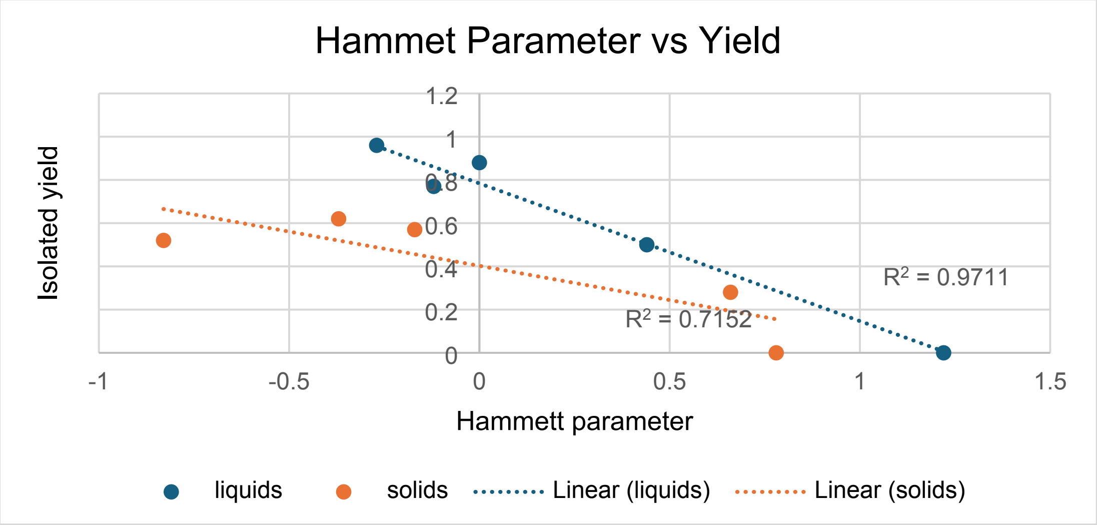

Figure S1 *Relationship between Hammett parameter of aryl substituent and isolated yield of the aryl thioglycoside*

Table S1 *Changes in yield after adding aryl halide to reaction mixture as solution (10 M in THF)*

	Product	Initial yield (%)	Yield[a] (%)
**5**	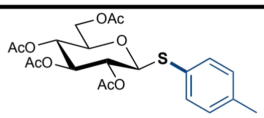	57	69
**7**	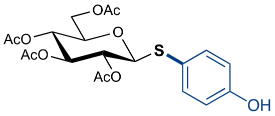	62	86
**11**	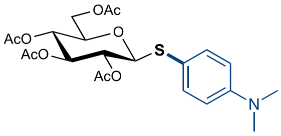	52	79
**12**	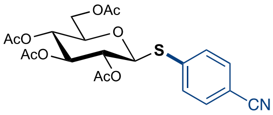	28	56

a) yield when solid aryl halide was added as solution (10 M in THF)

^{1}H NMR Spectrum of compound 3 (400 MHz, $CDCl_3$)

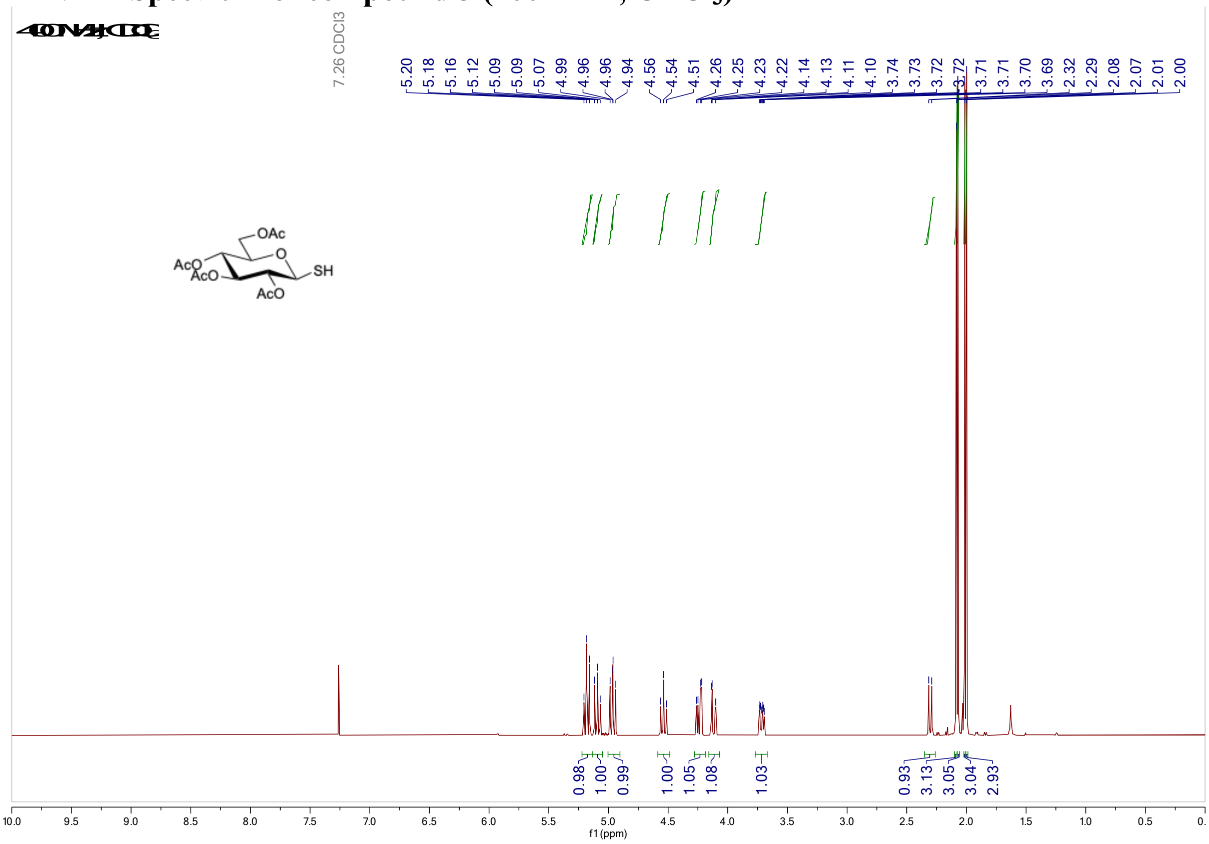

^{13}C{^{1}H} NMR Spectrum of compound 3 (101 MHz, $CDCl_3$)

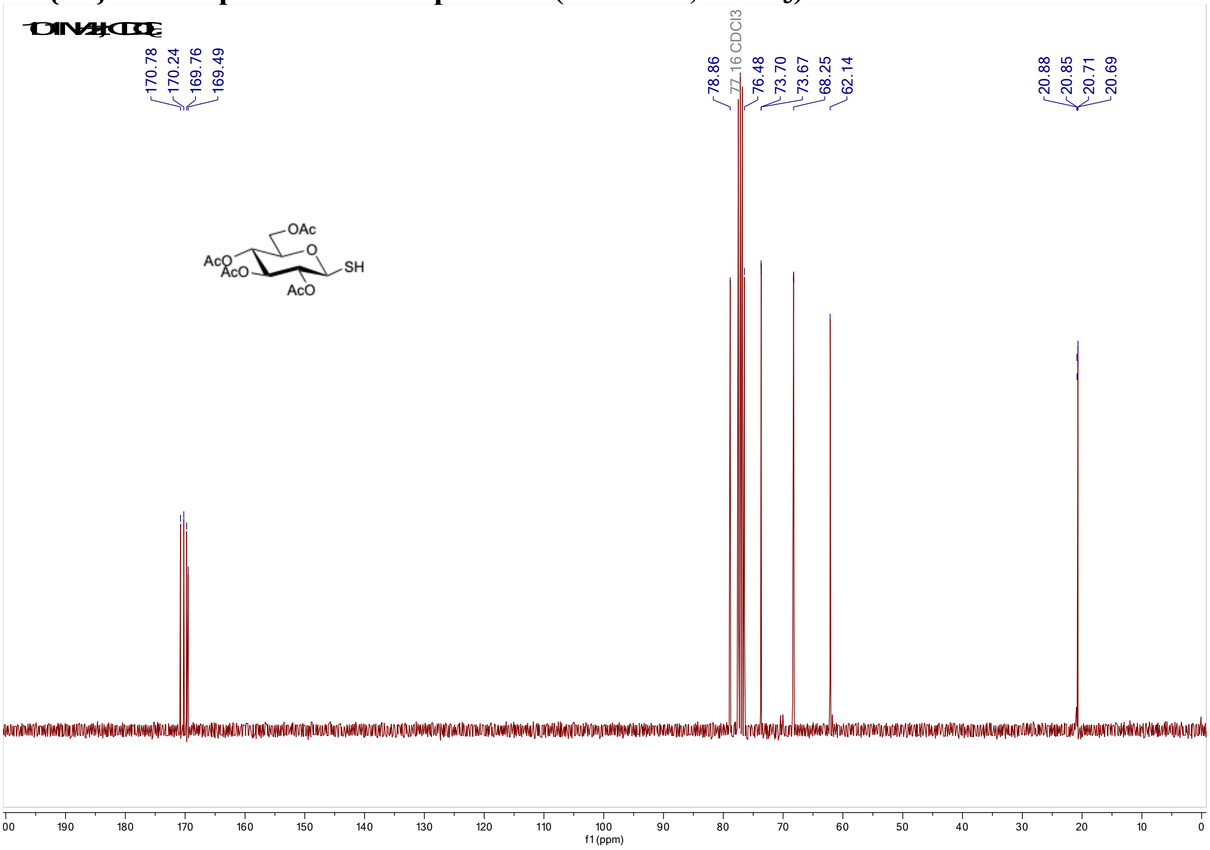

^{1}H NMR Spectrum of compound 4 (500 MHz, $CDCl_3$)

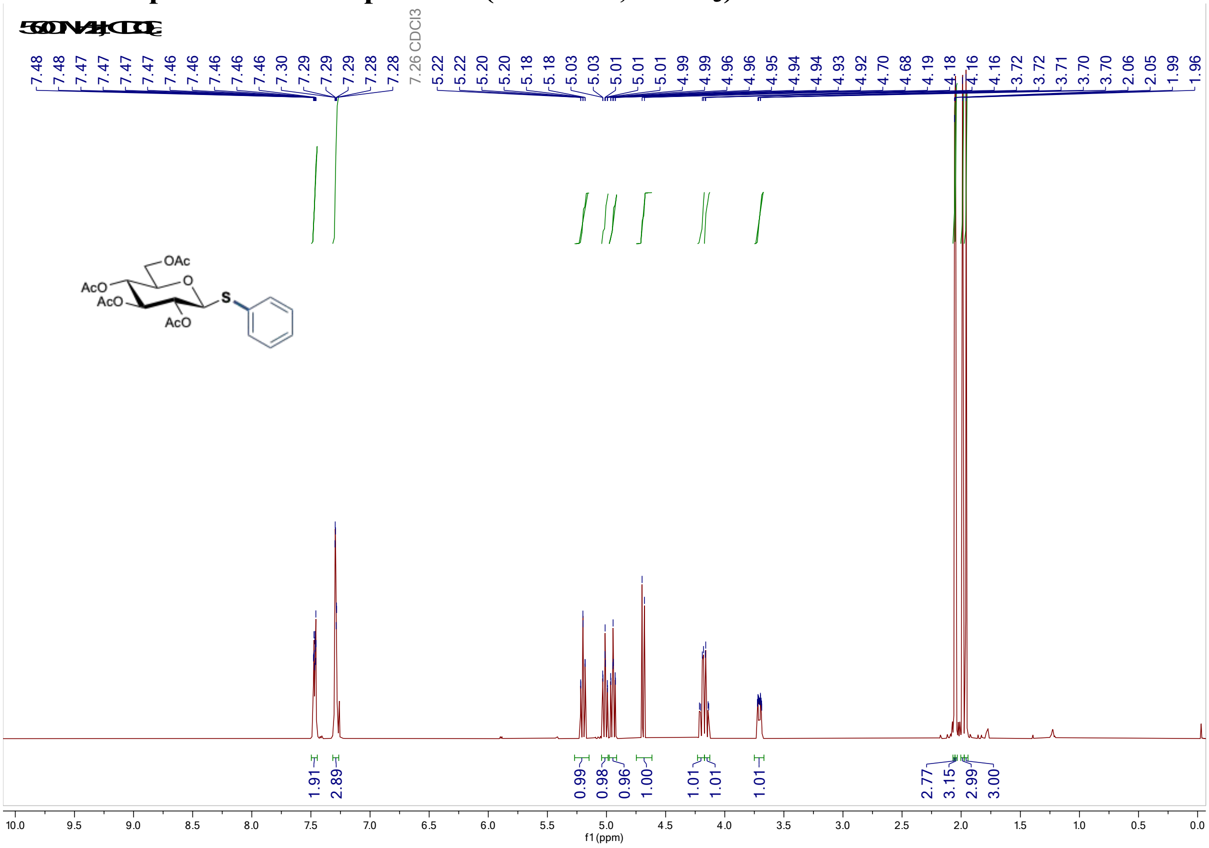

^{13}C{^{1}H} NMR Spectrum of compound 4 (126 MHz, $CDCl_3$)

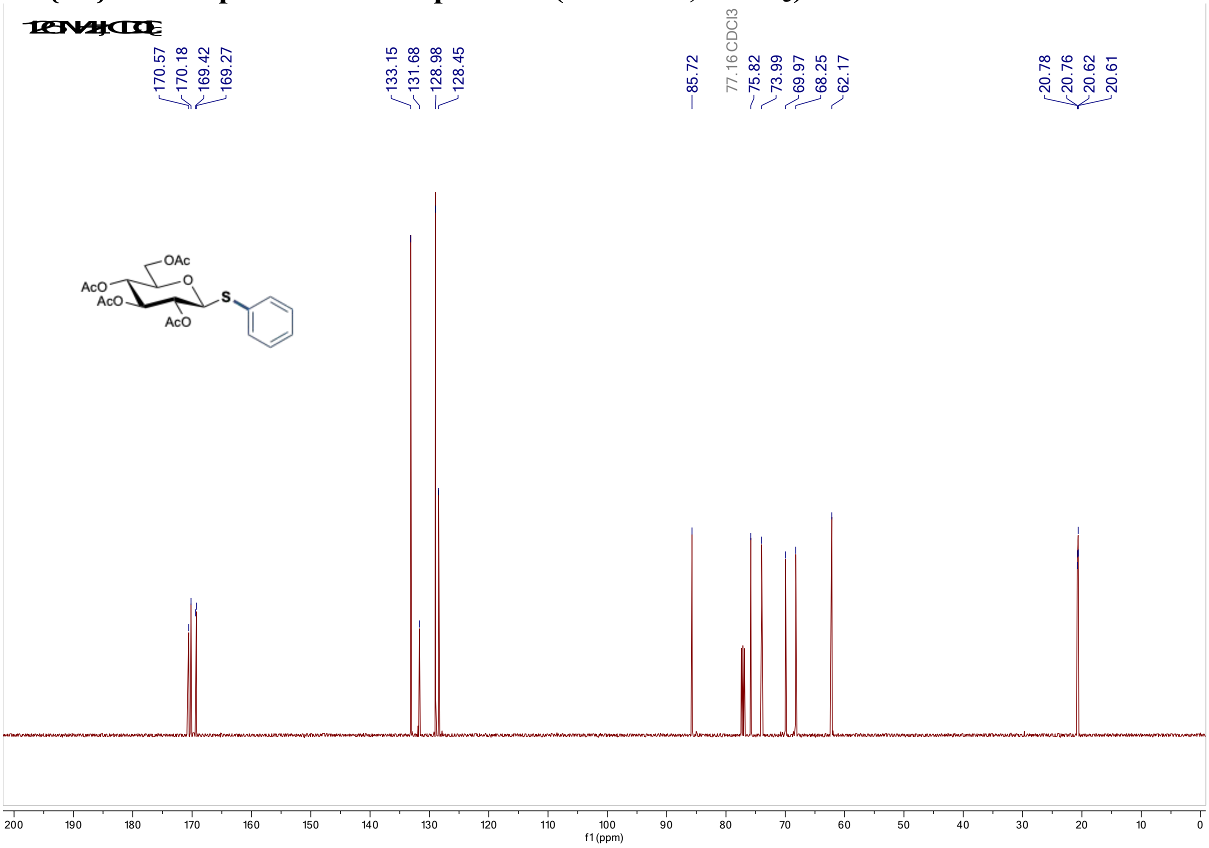

^{1}H NMR Spectrum of compound 5 (400 MHz, $CDCl_3$)

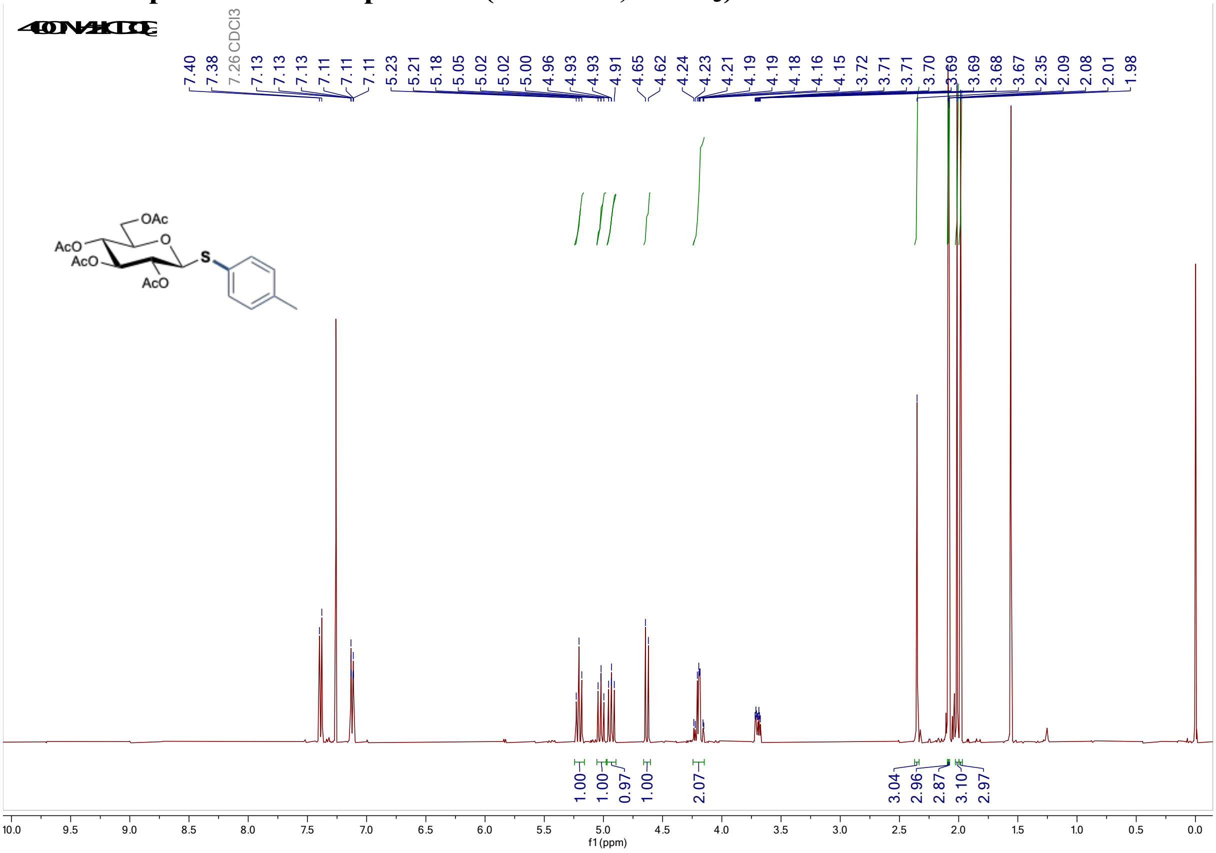

$^{13}C\{^{1}H\}$ NMR Spectrum of compound 5 (101 MHz, $CDCl_3$)

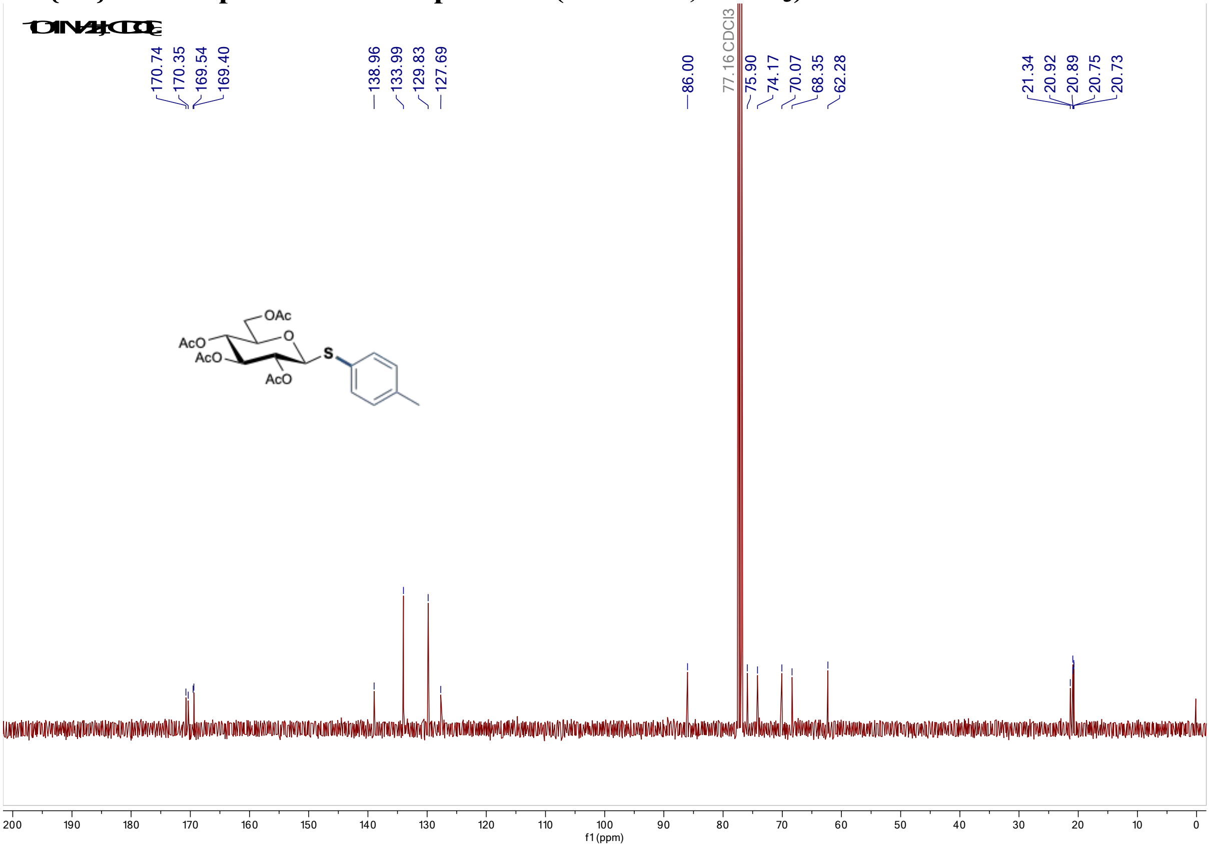

^{1}H NMR Spectrum of compound 6 (400 MHz, $CDCl_3$)

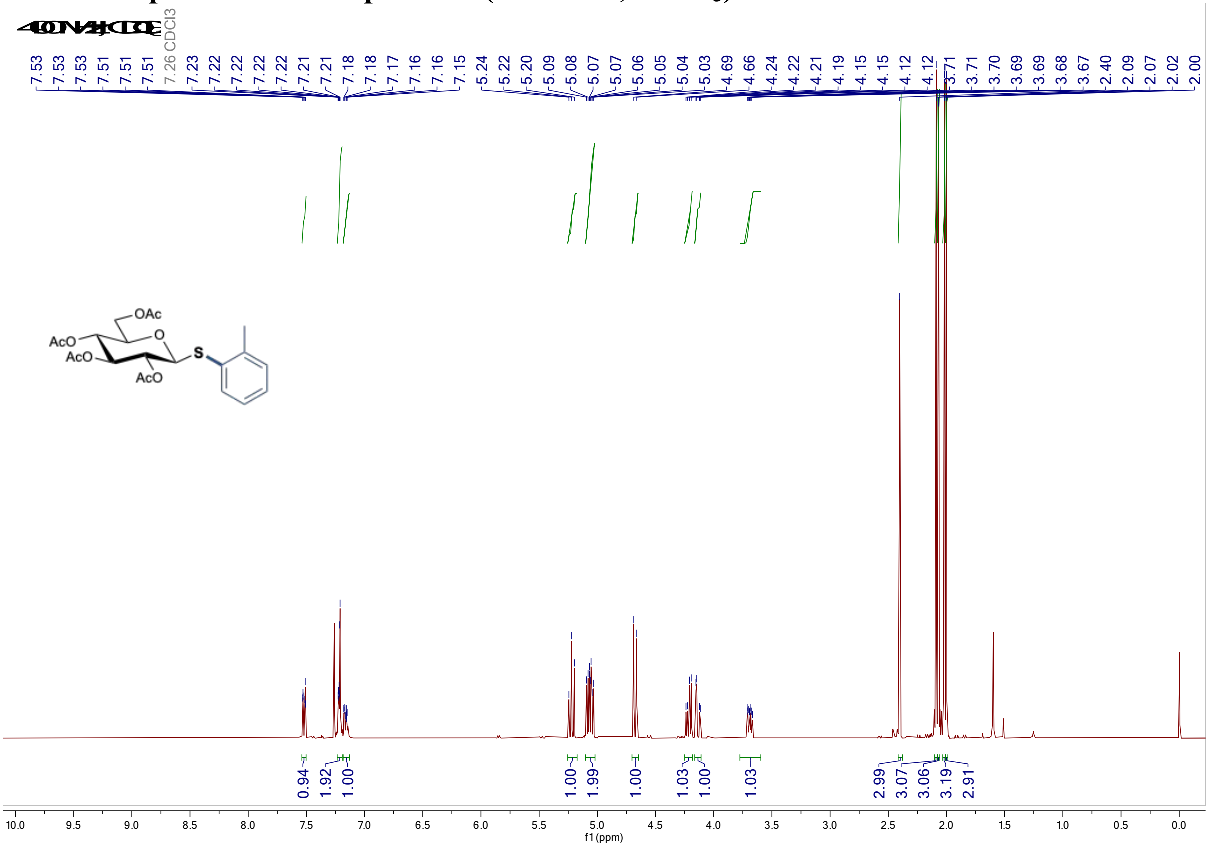

^{13}C{^{1}H} NMR Spectrum of compound 6 (101 MHz, $CDCl_3$)

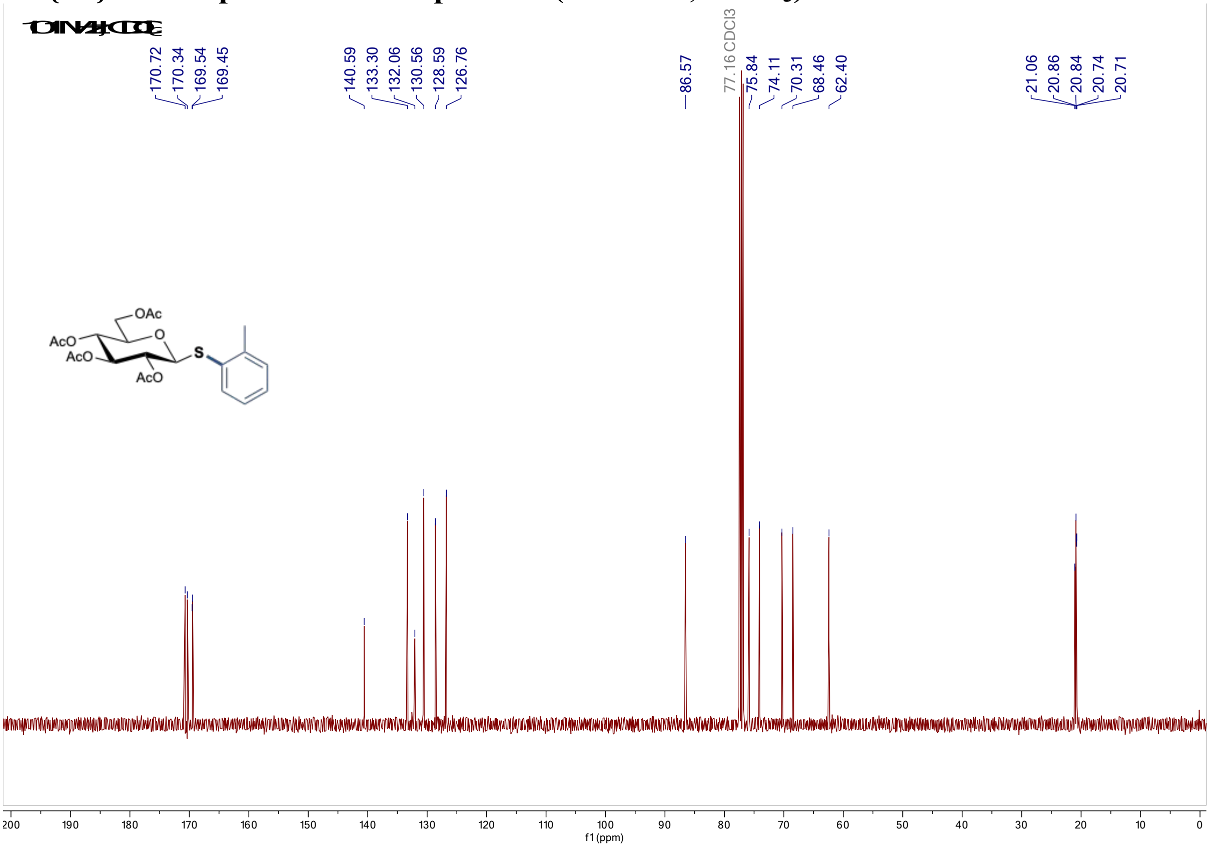

^{1}H NMR Spectrum of compound 7 (400 MHz, $CDCl_3$)

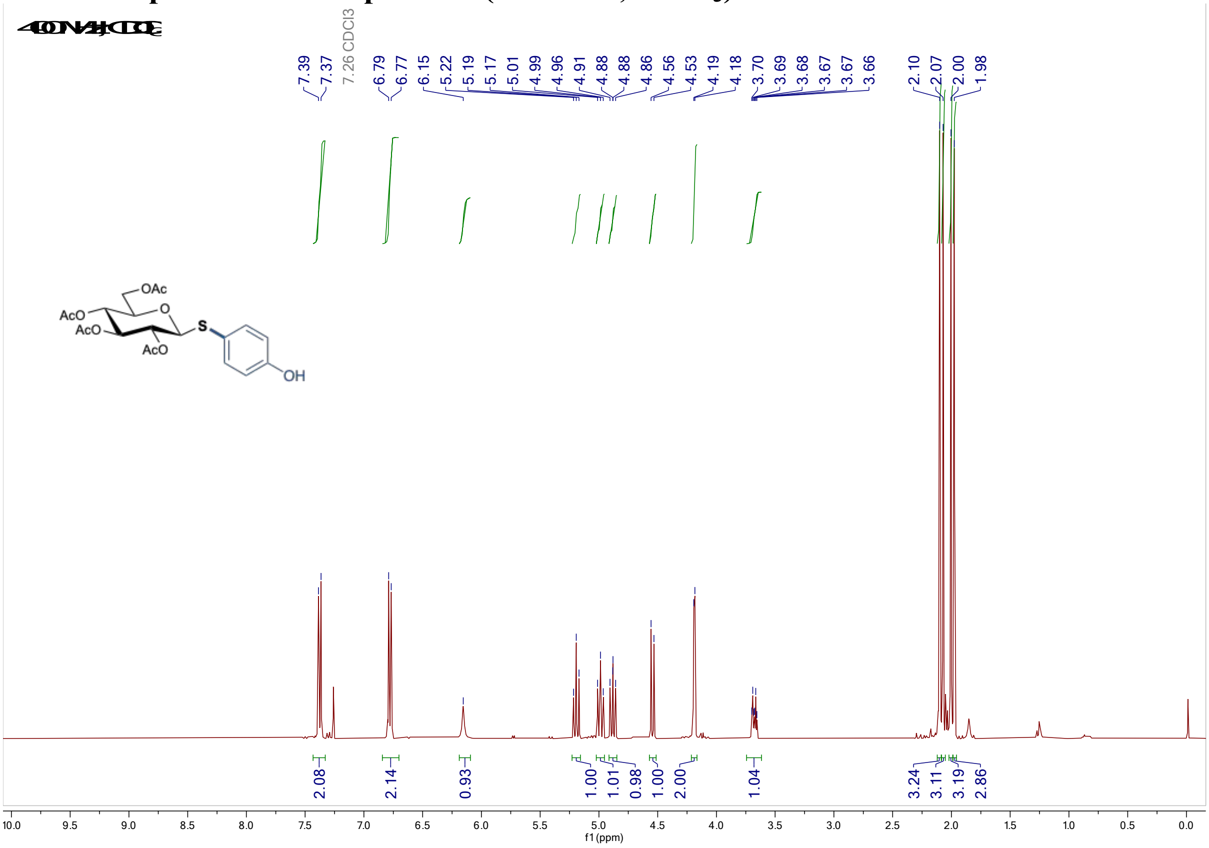

$^{13}C\{^{1}H\}$ NMR Spectrum of compound 7 (101 MHz, $CDCl_3$)

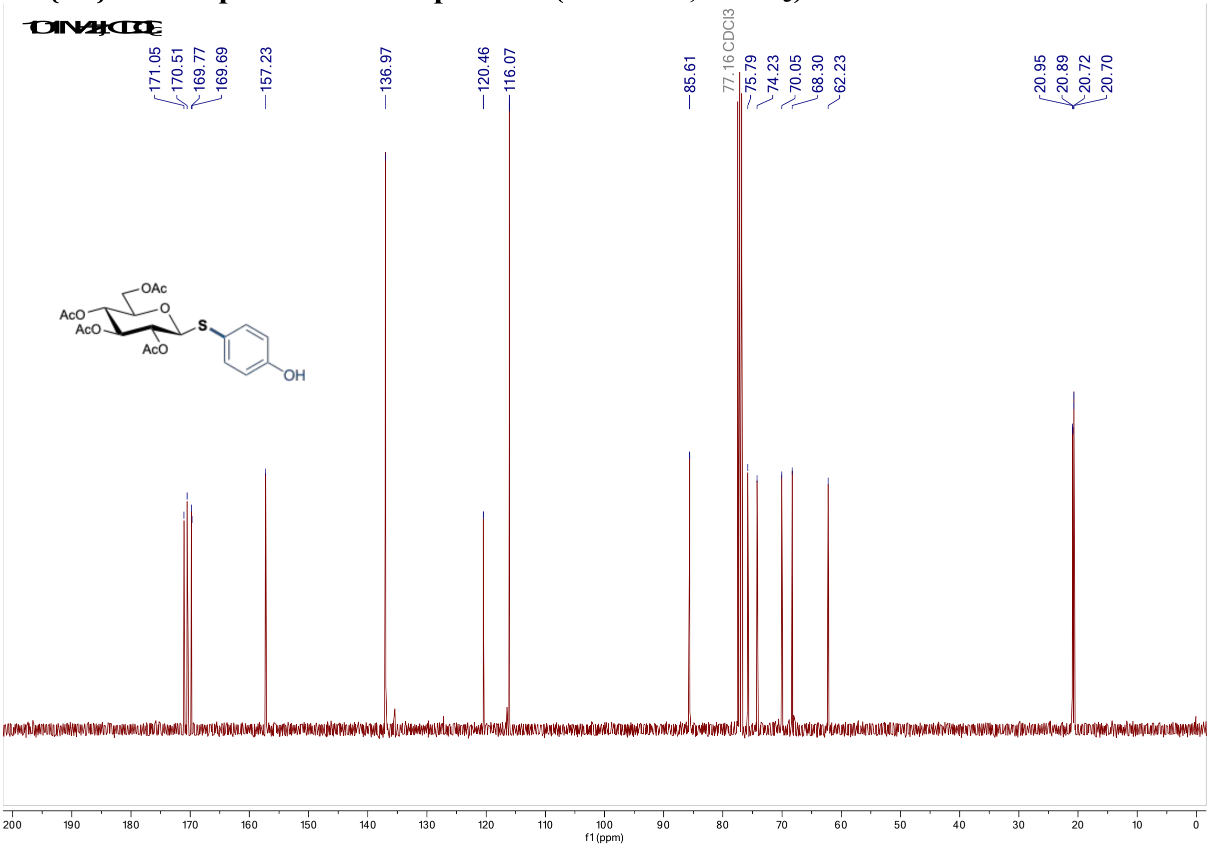

^{1}H NMR Spectrum of compound 8 (400 MHz, $CDCl_3$)

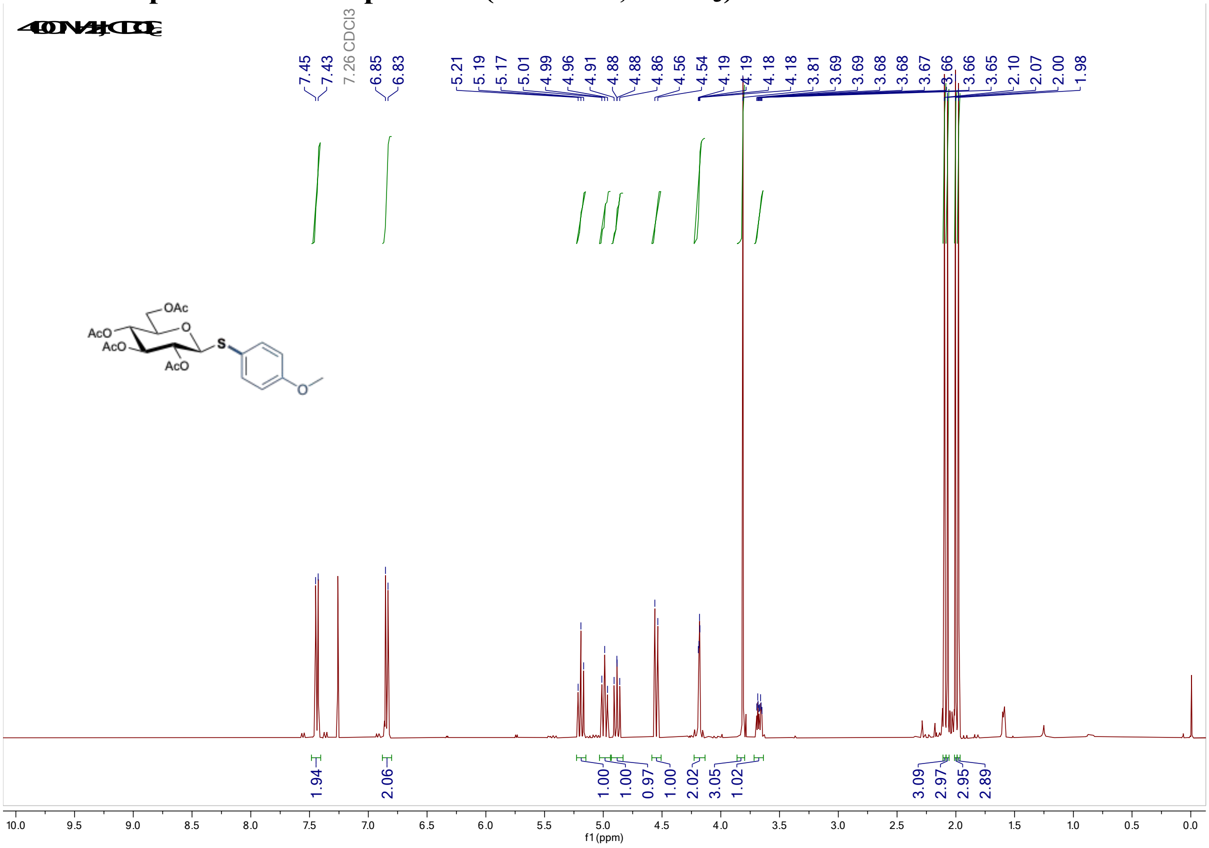

$^{13}C\{^{1}H\}$ NMR Spectrum of compound 8 (101 MHz, $CDCl_3$)

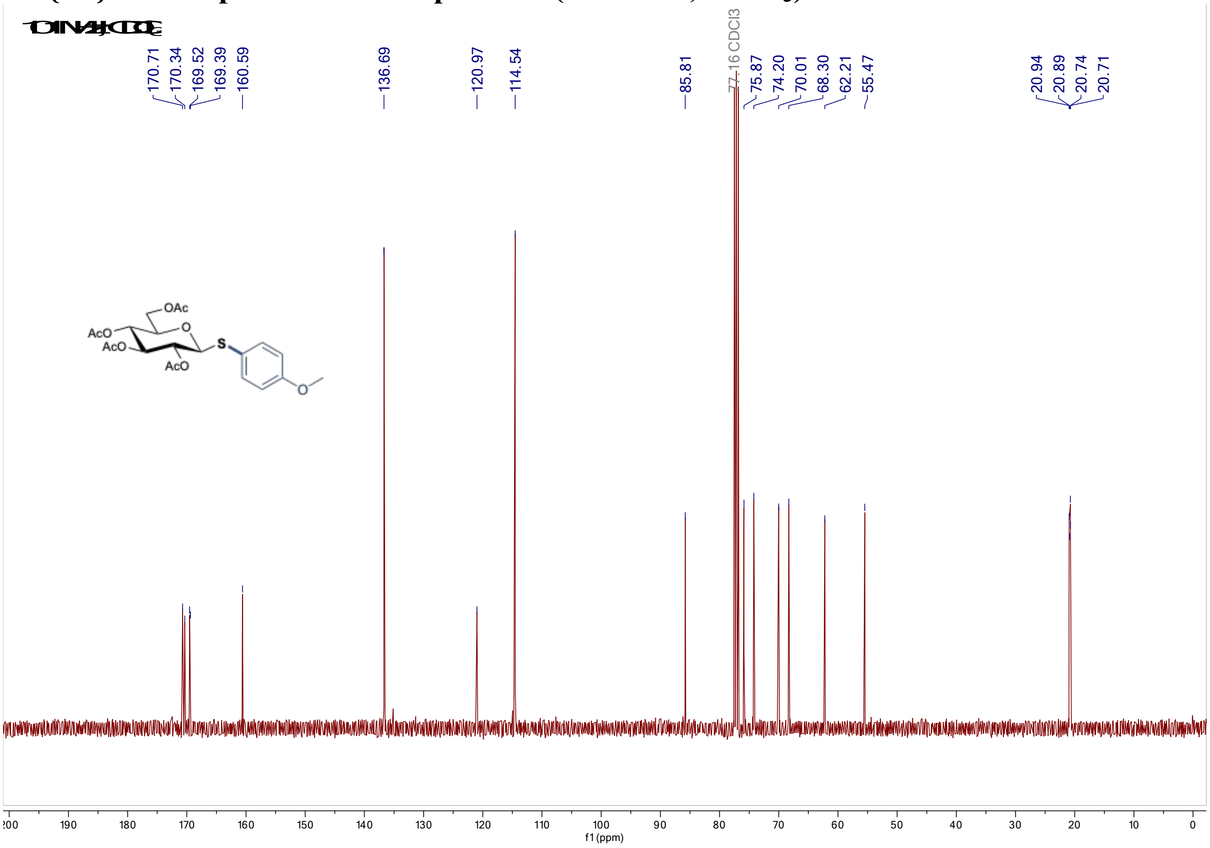

^{1}H NMR Spectrum of compound 9 (400 MHz, $CDCl_3$)

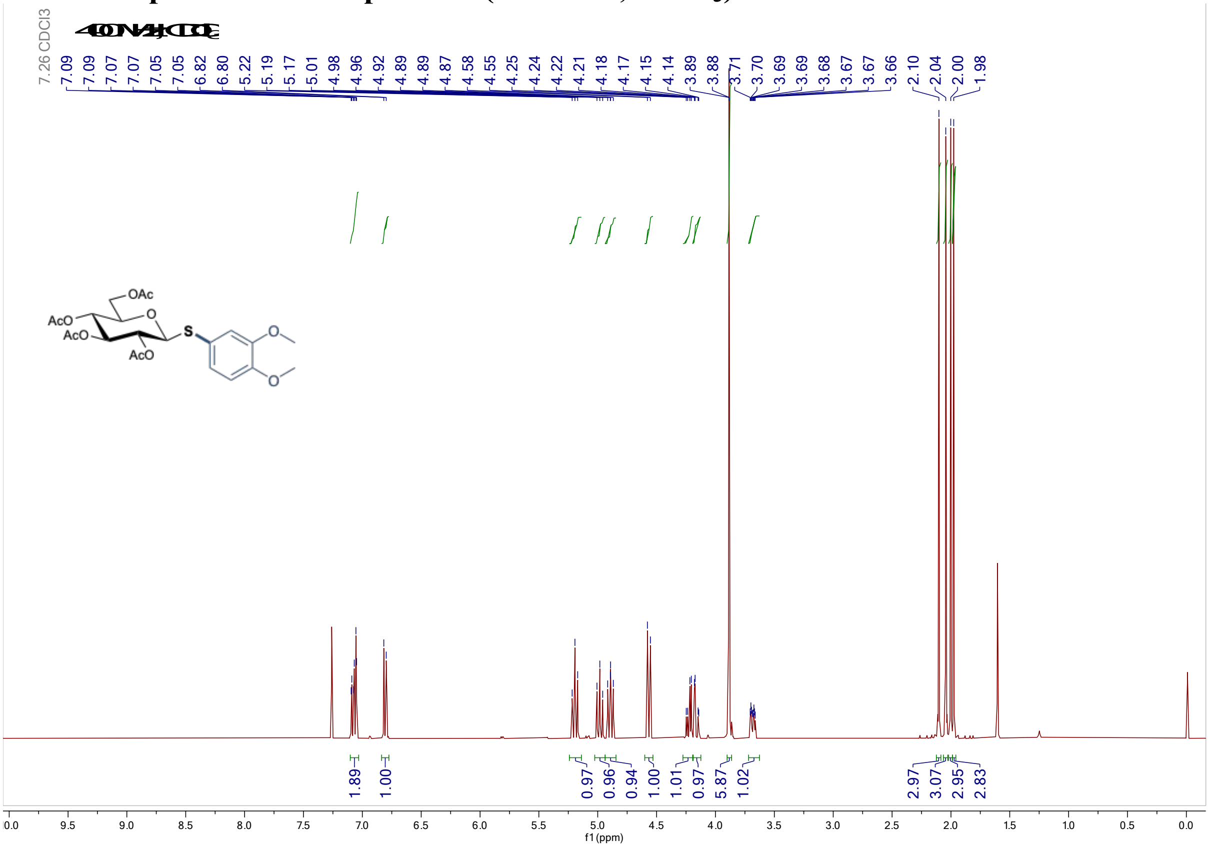

^{13}C{^{1}H} NMR Spectrum of compound 9 (101 MHz, $CDCl_3$)

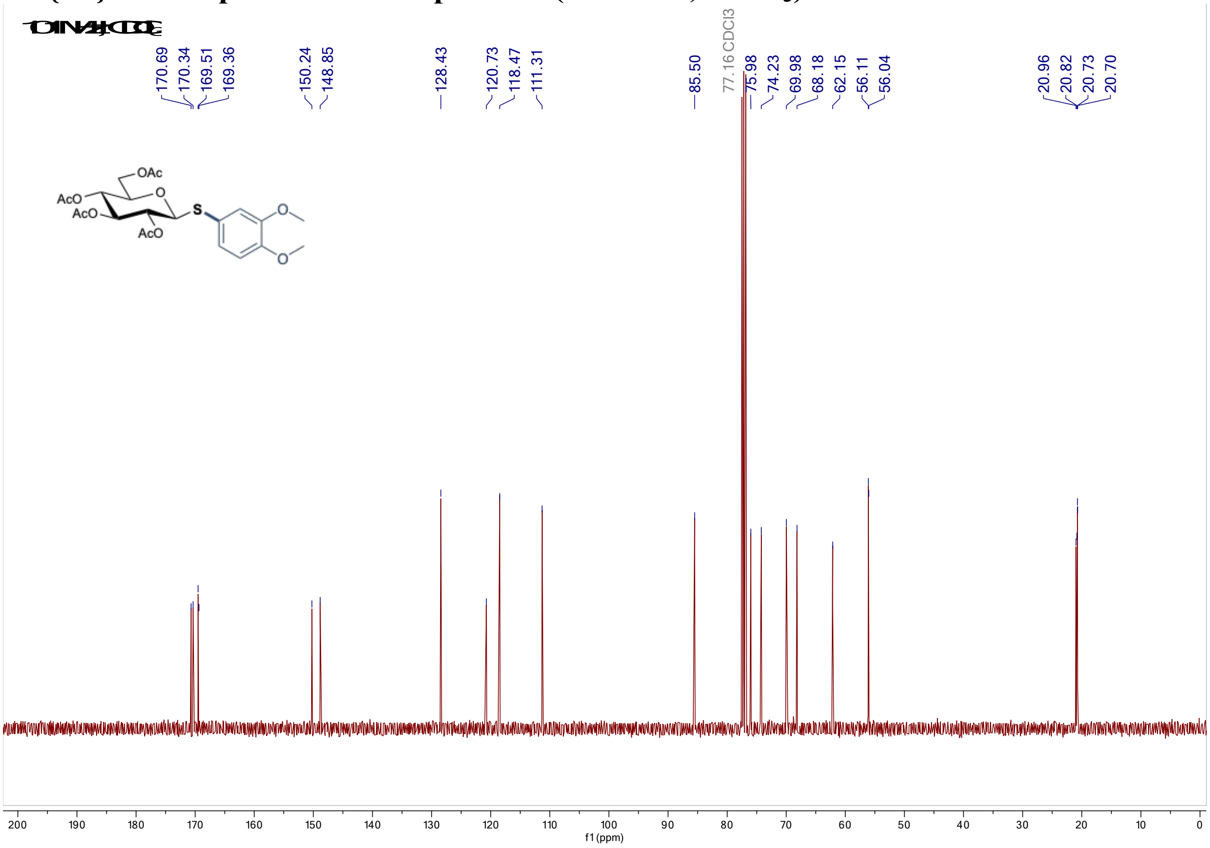

^{1}H NMR Spectrum of compound 10 (400 MHz, $CDCl_3$)

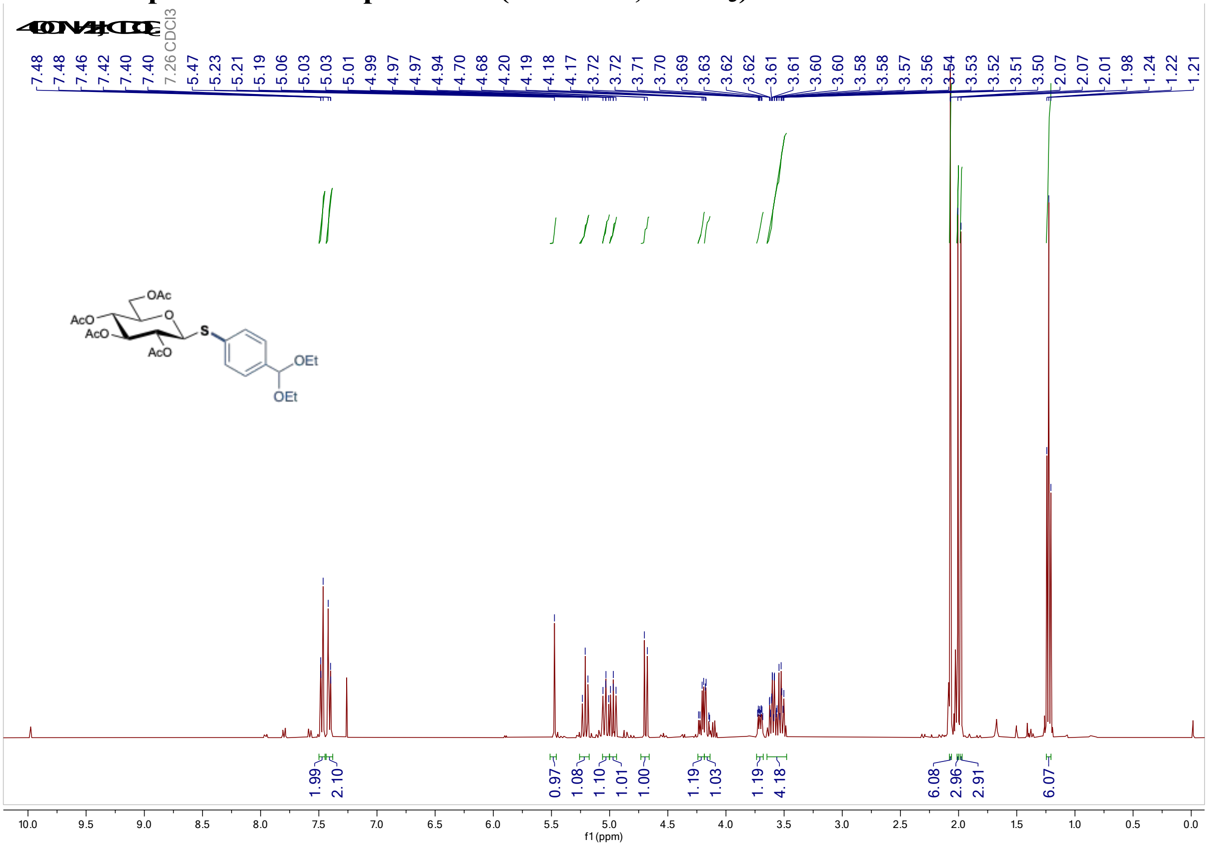

$^{13}C\{^{1}H\}$ NMR Spectrum of compound 10 (101 MHz, $CDCl_3$)

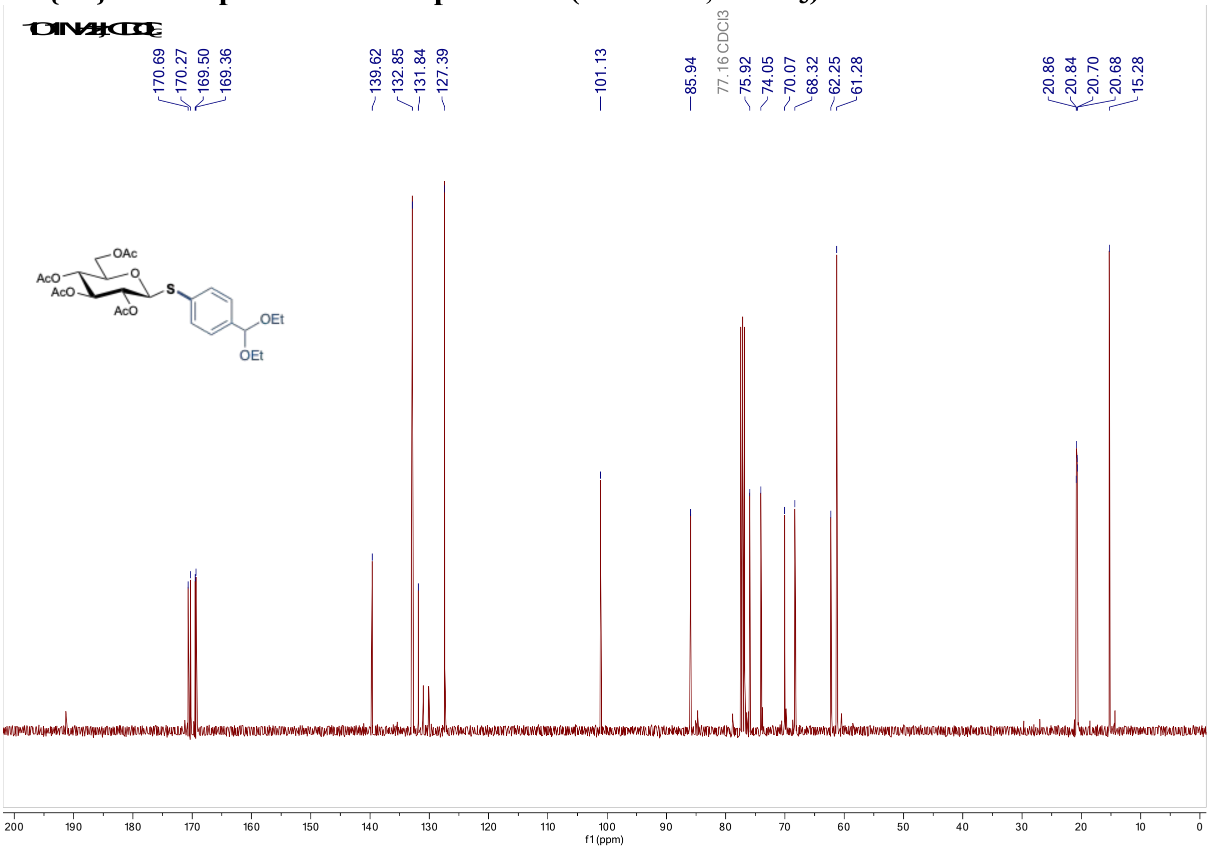

^{1}H NMR Spectrum of compound 11 (400 MHz, $CDCl_3$)

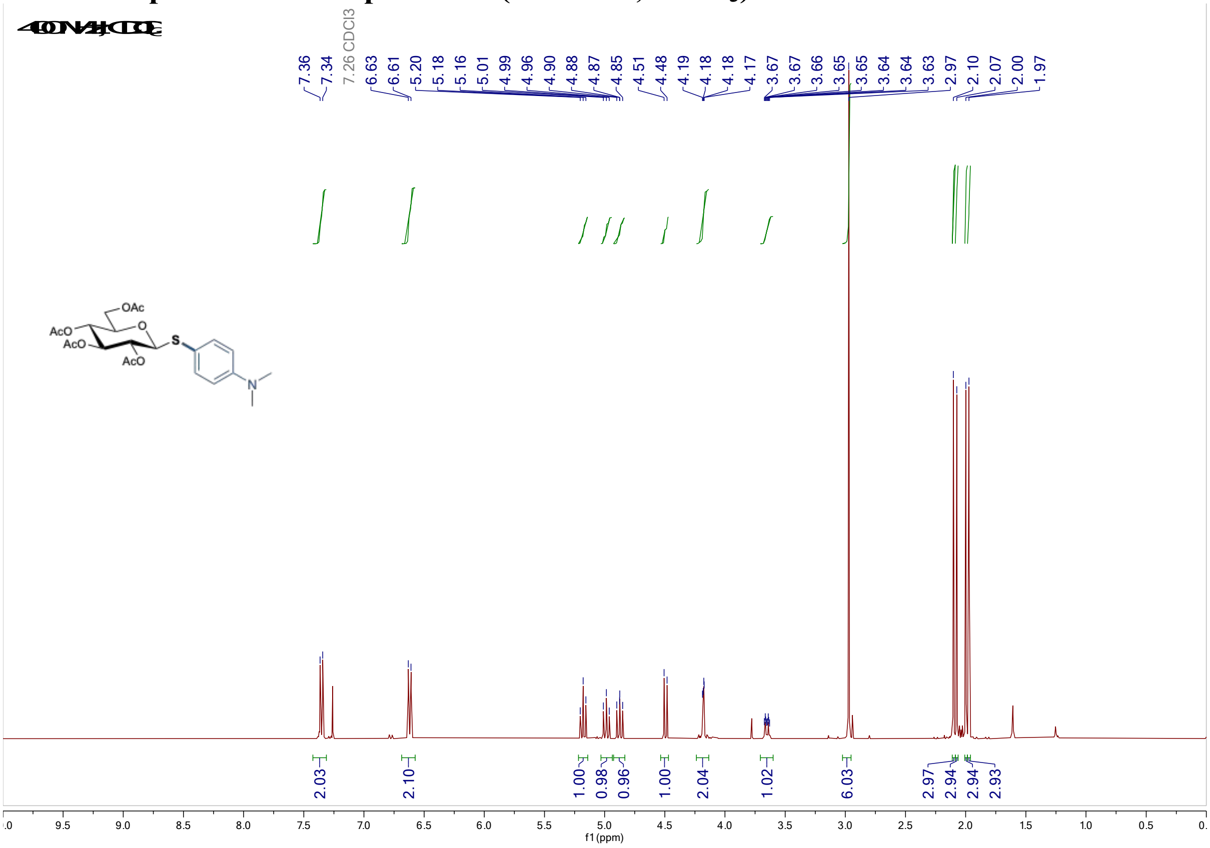

^{13}C{^{1}H} NMR Spectrum of compound 11 (101 MHz, $CDCl_3$)

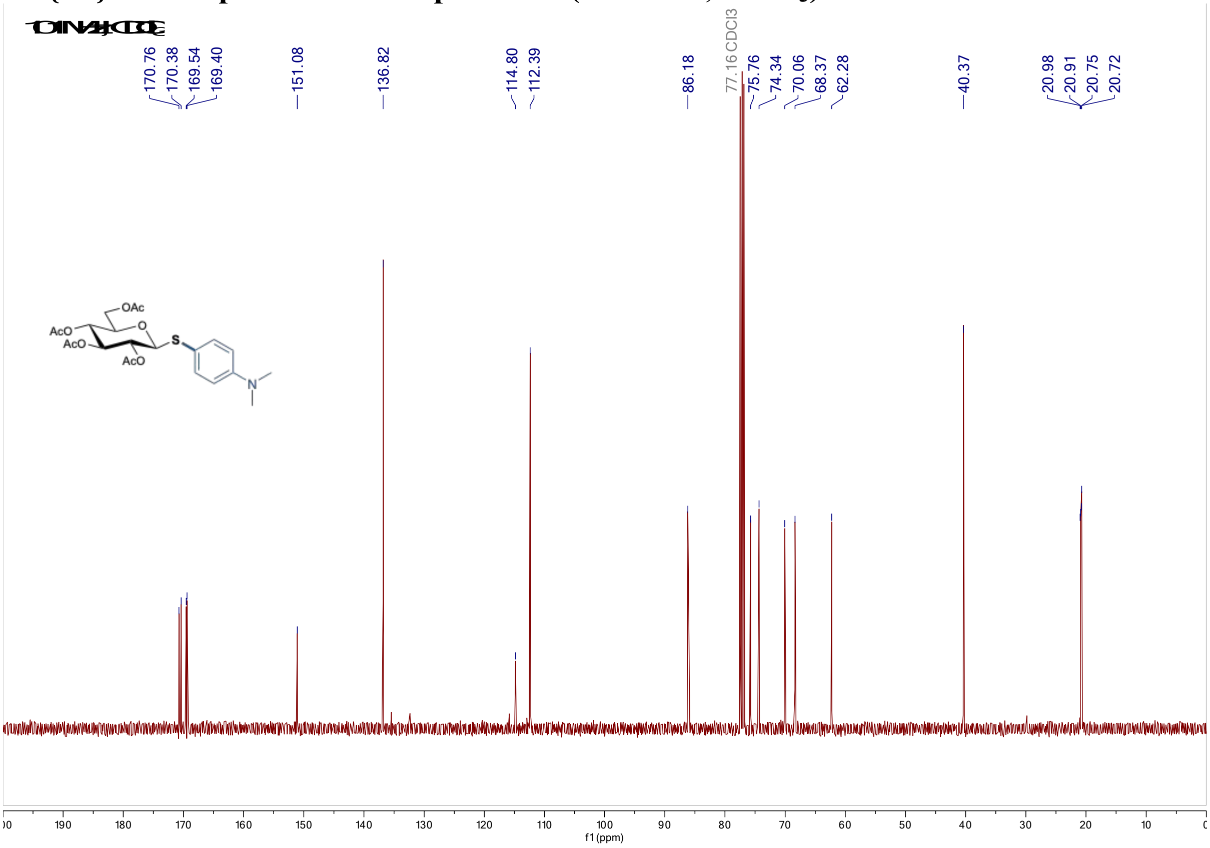

^{1}H NMR Spectrum of compound 12 (400 MHz, $CDCl_3$)

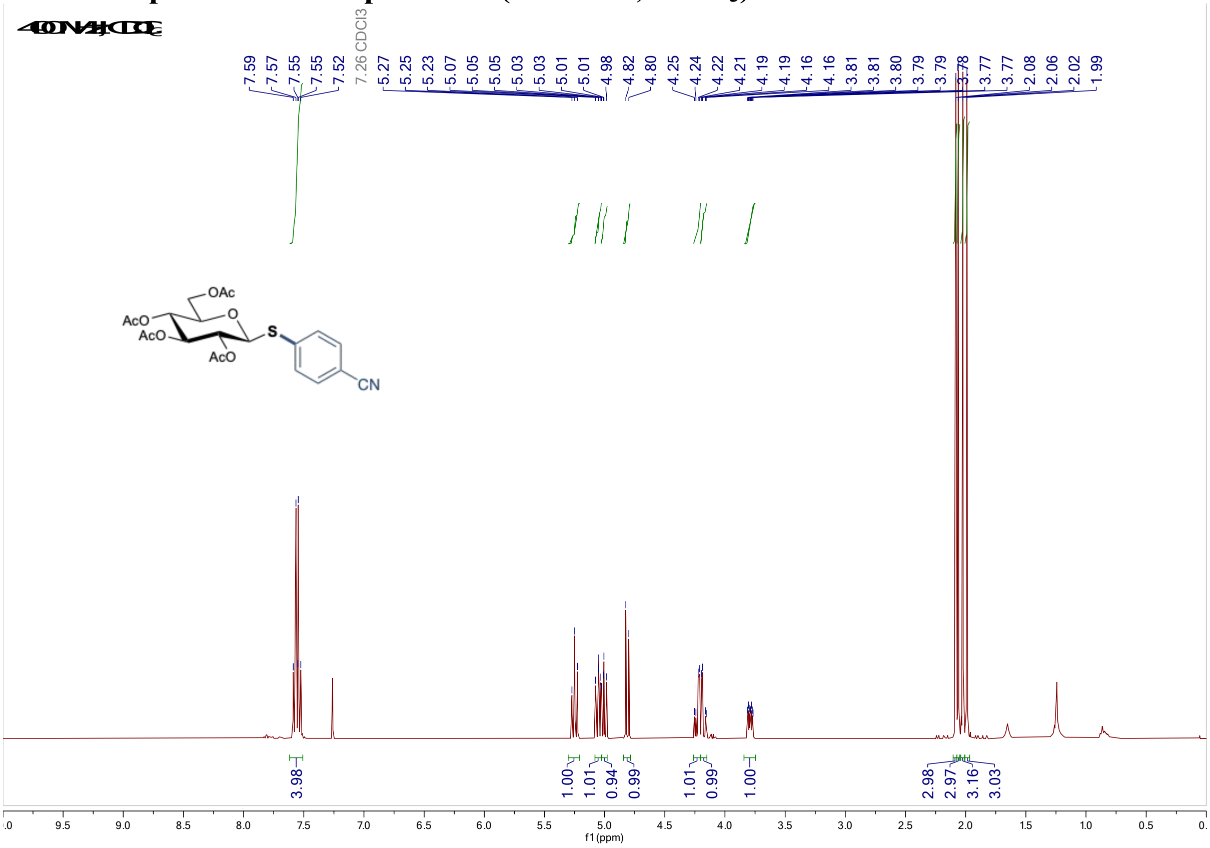

^{13}C{^{1}H} NMR Spectrum of compound 12 (101 MHz, $CDCl_3$)

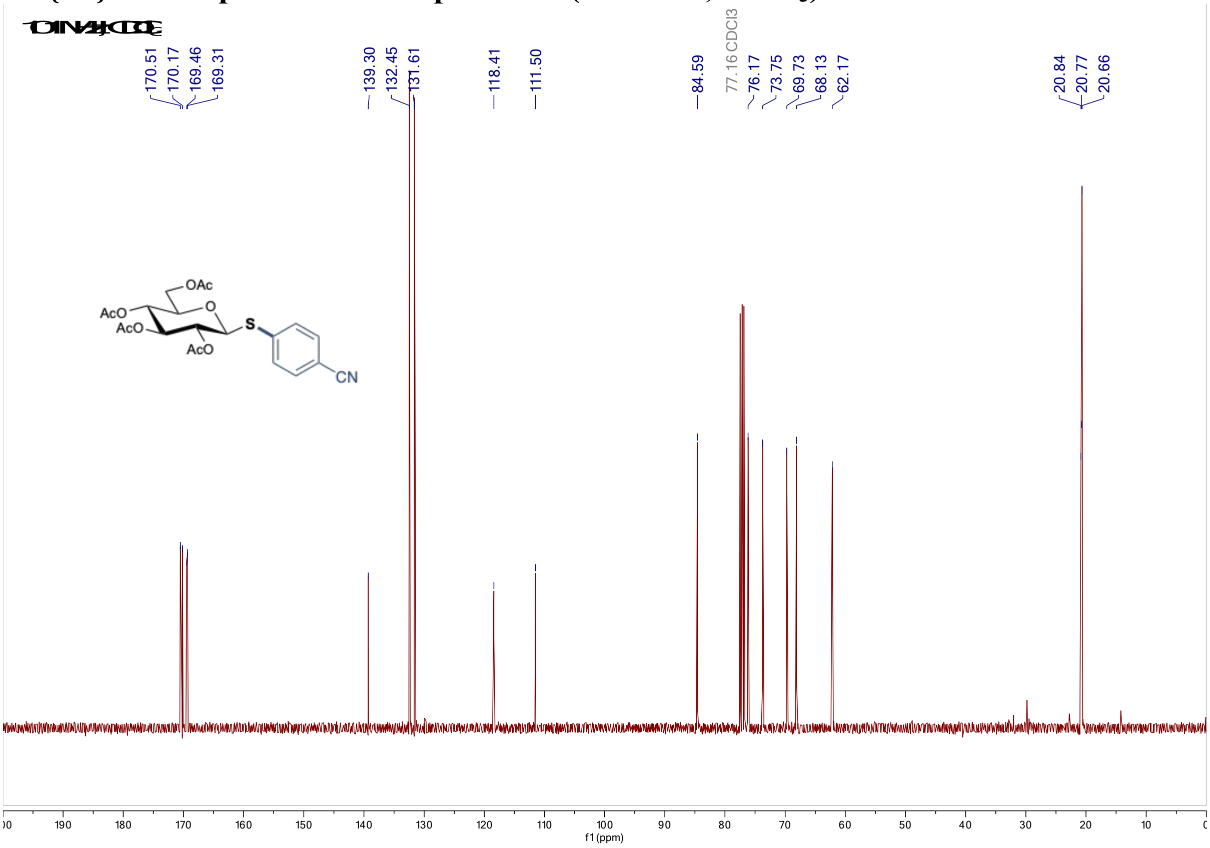

^{1}H NMR Spectrum of compound 13 (500 MHz, $CDCl_3$)

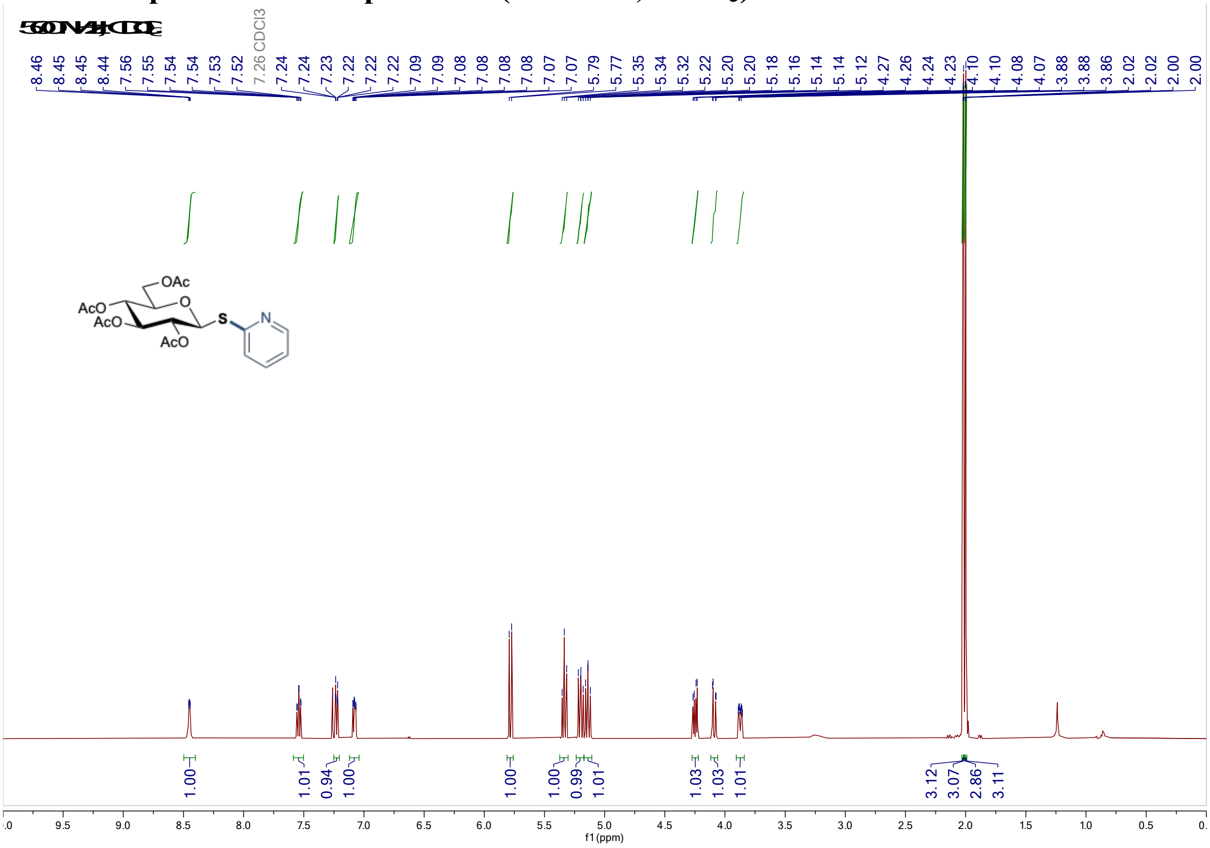

^{13}C{^{1}H} NMR Spectrum of compound 13 (126 MHz, $CDCl_3$)

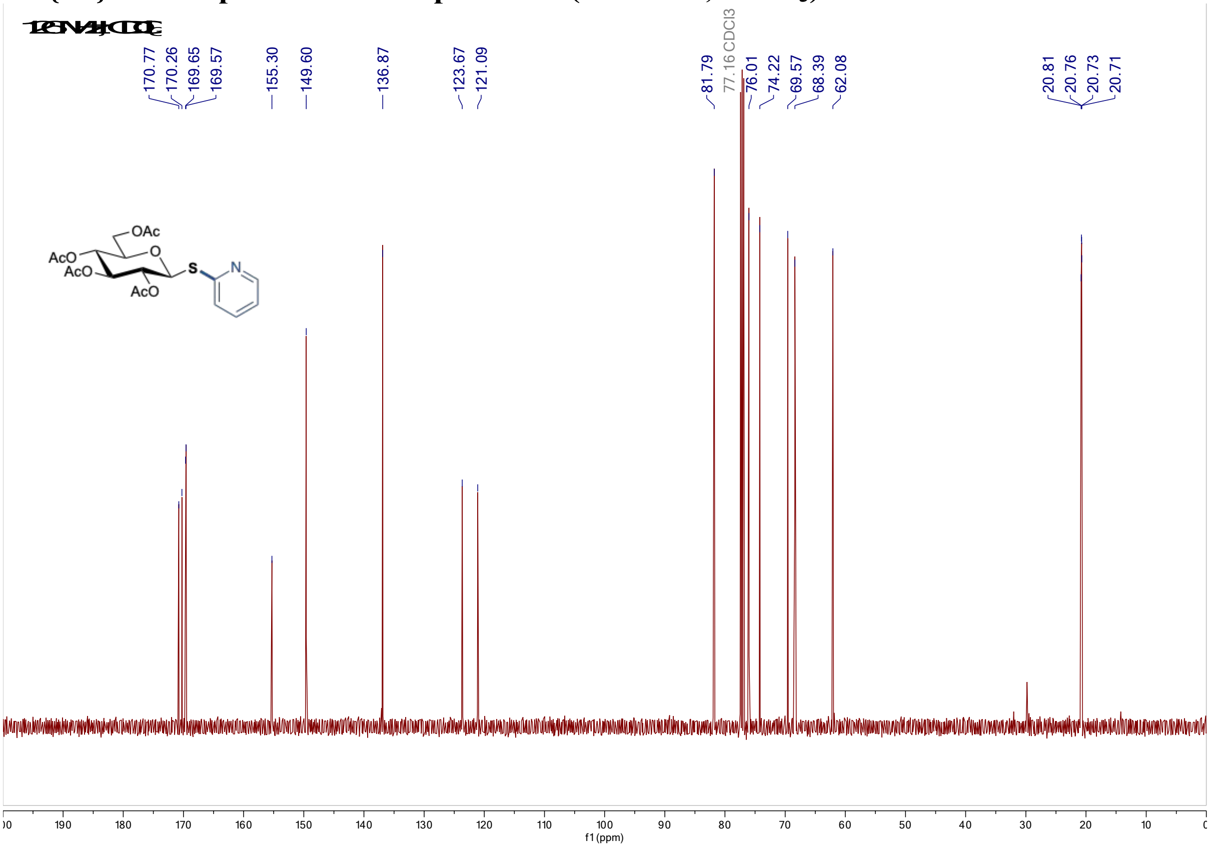

^{1}H NMR Spectrum of compound 14 (500 MHz, $CDCl_3$)

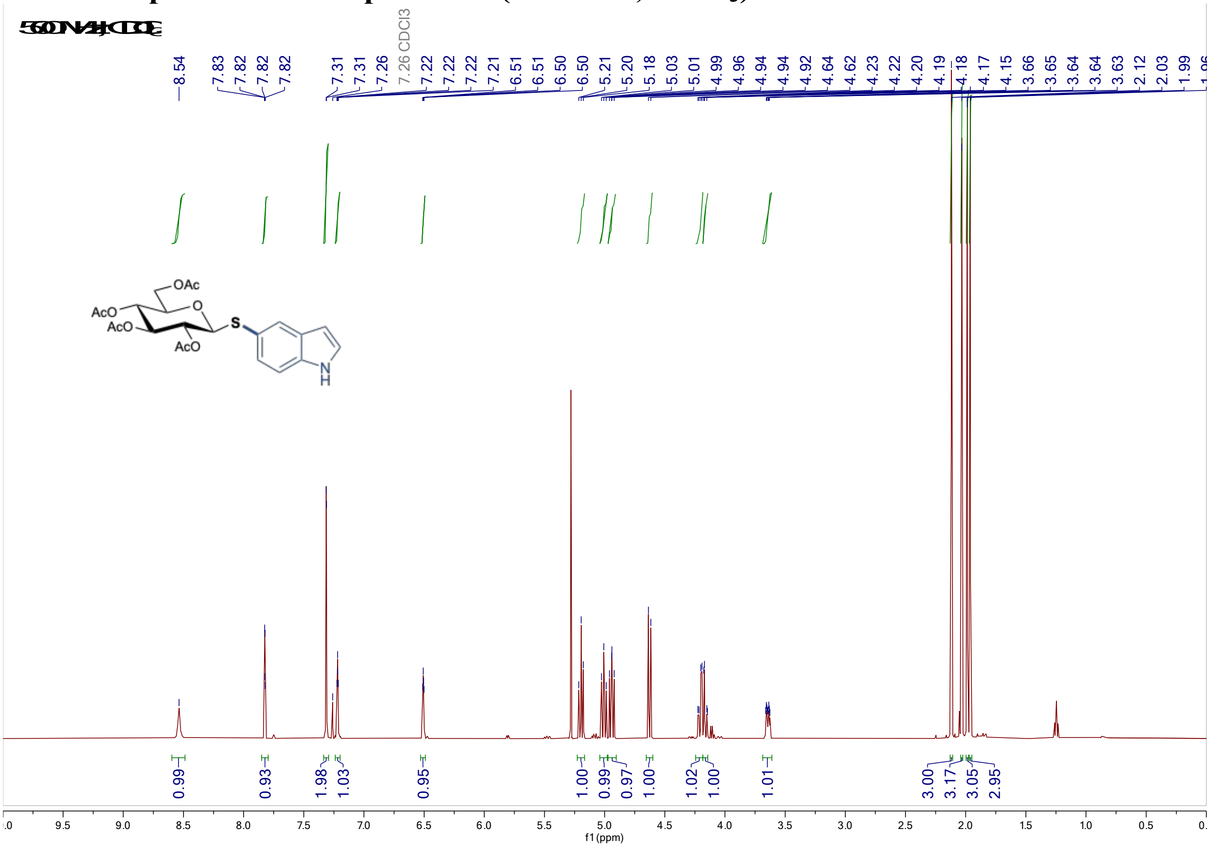

^{13}C{^{1}H} NMR Spectrum of compound 14 (126 MHz, $CDCl_3$)

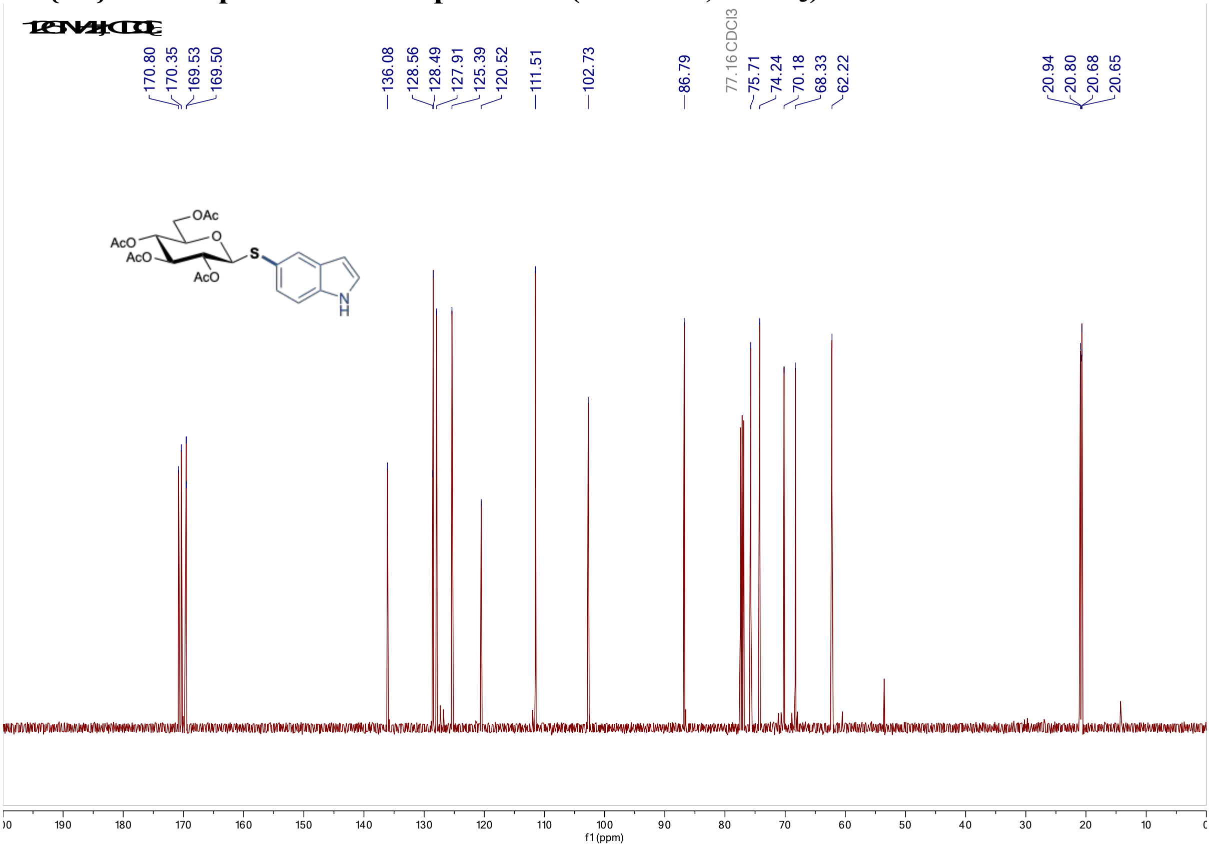

^{1}H NMR Spectrum of compound 15 (500 MHz, $CDCl_3$)

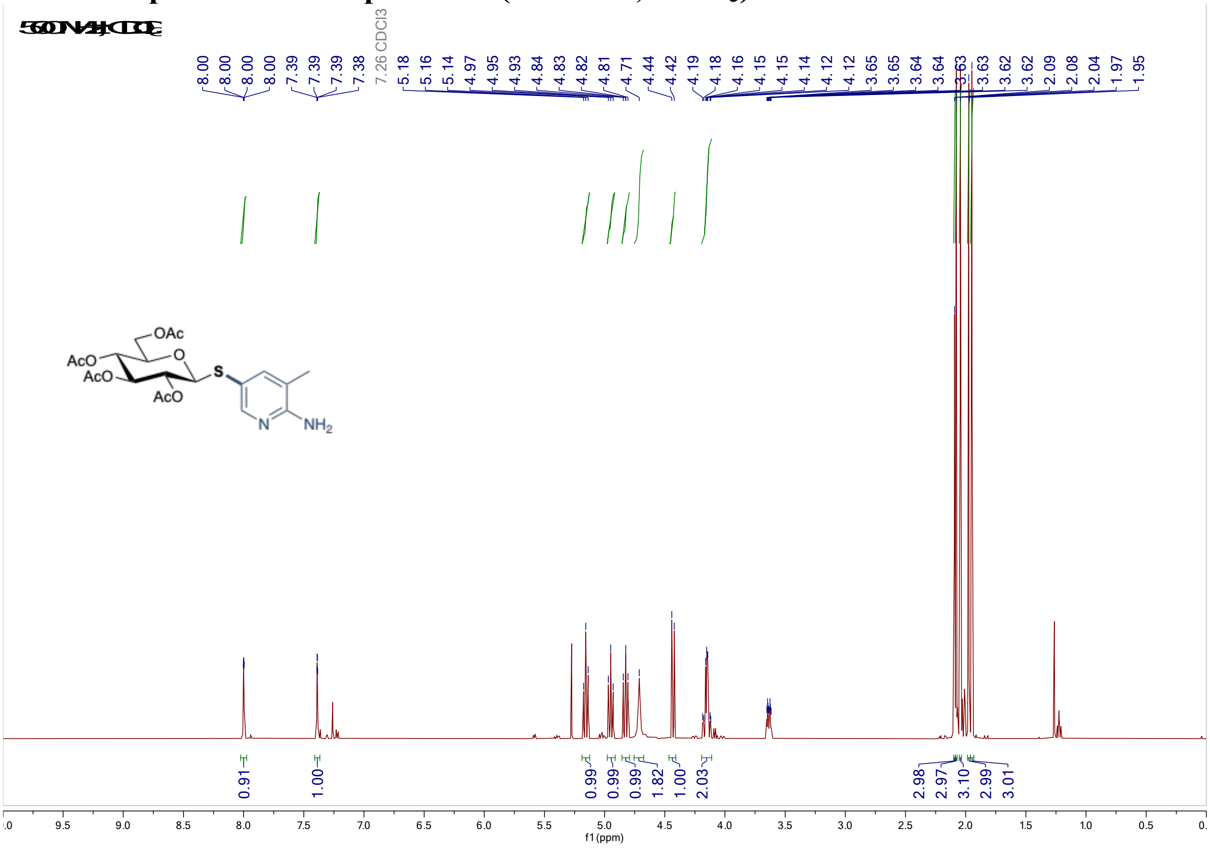

$^{13}C\{^1H\}$ NMR Spectrum of compound 15 (126 MHz, $CDCl_3$)

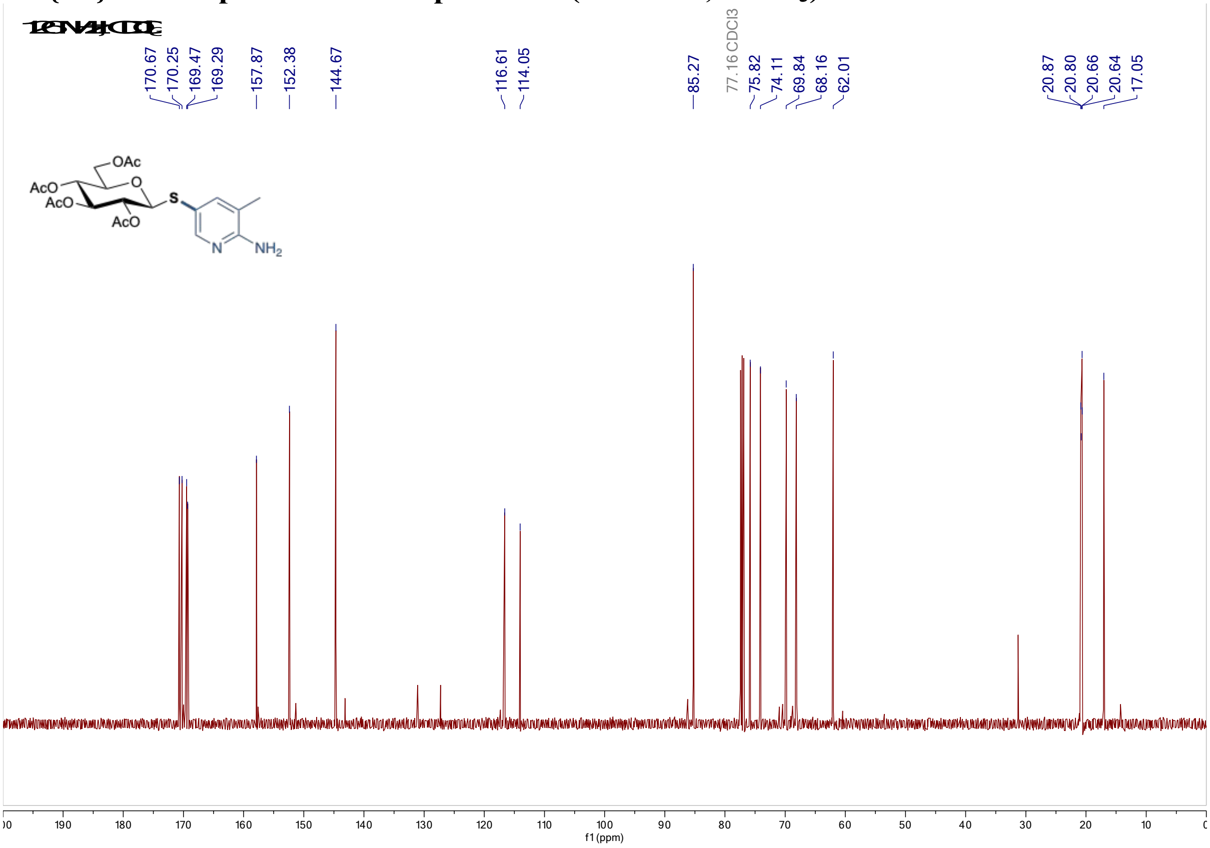

^{1}H NMR Spectrum of compound 16 (400 MHz, CDCl$_3$)

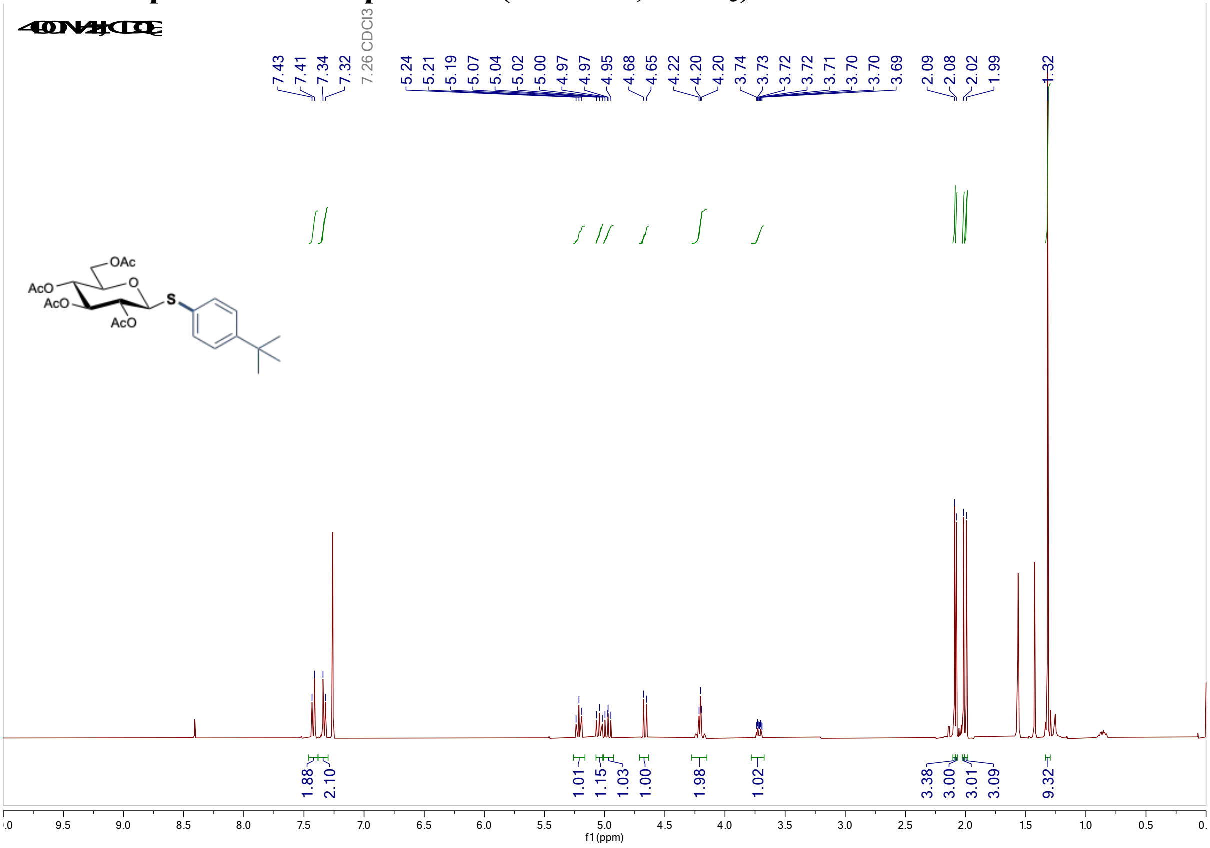

^{13}C{^{1}H} NMR Spectrum of compound 16 (101 MHz, CDCl$_3$)

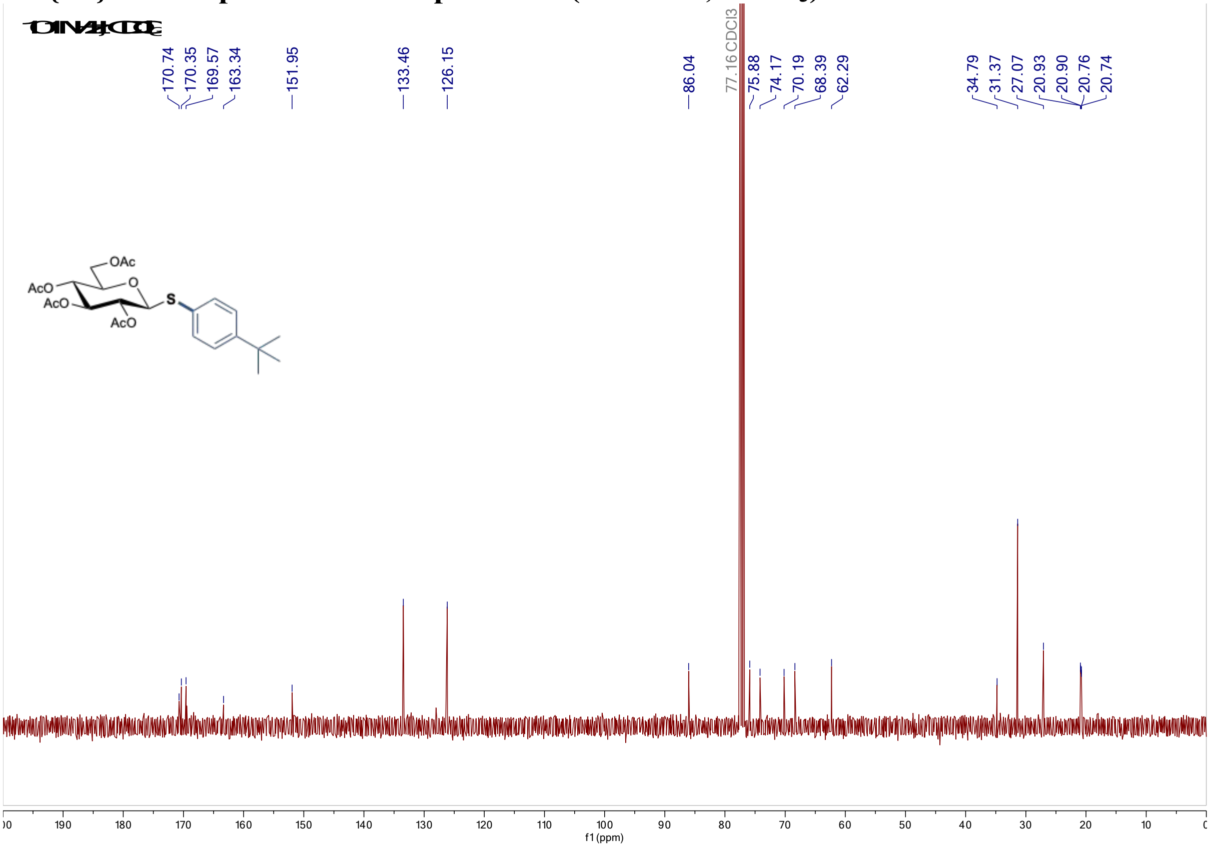

^{1}H NMR Spectrum of compound 17 (400 MHz, $CDCl_3$)

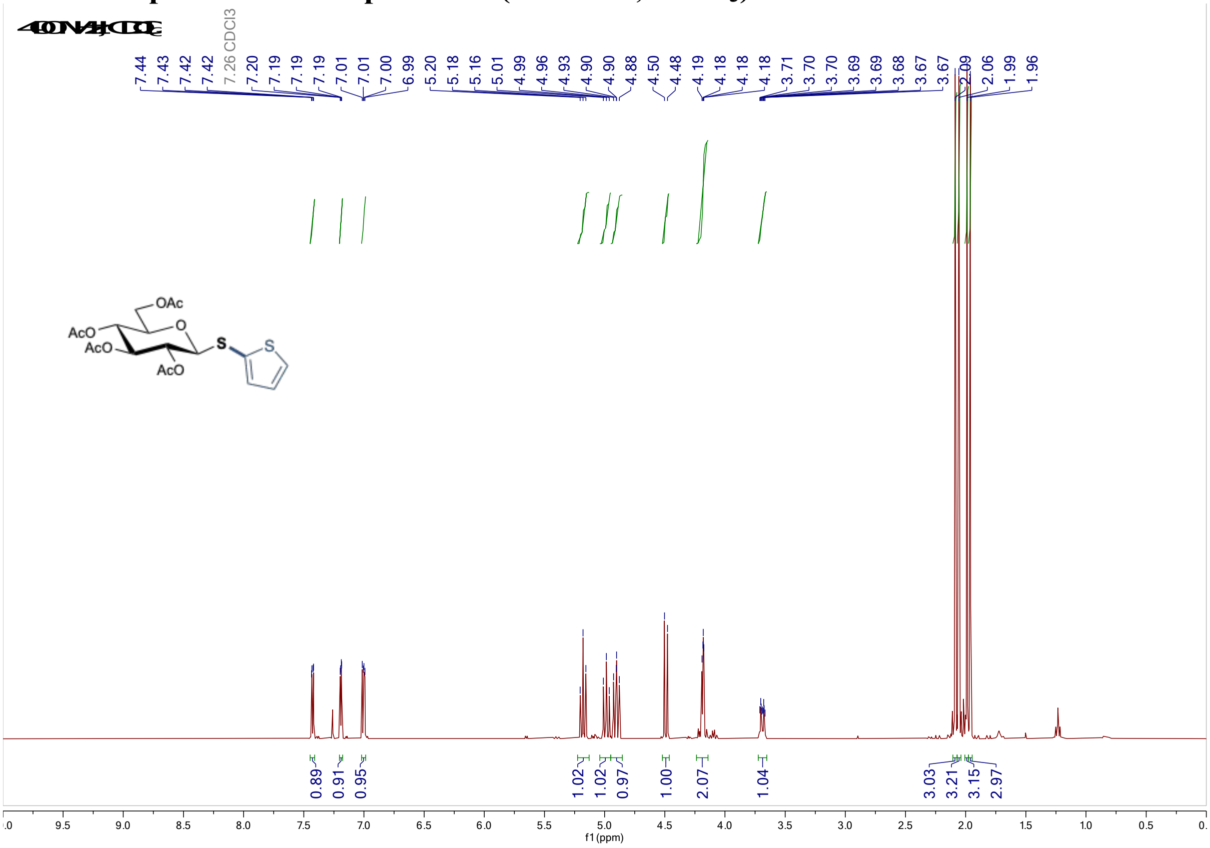

^{13}C{^{1}H} NMR Spectrum of compound 17 (101 MHz, $CDCl_3$)

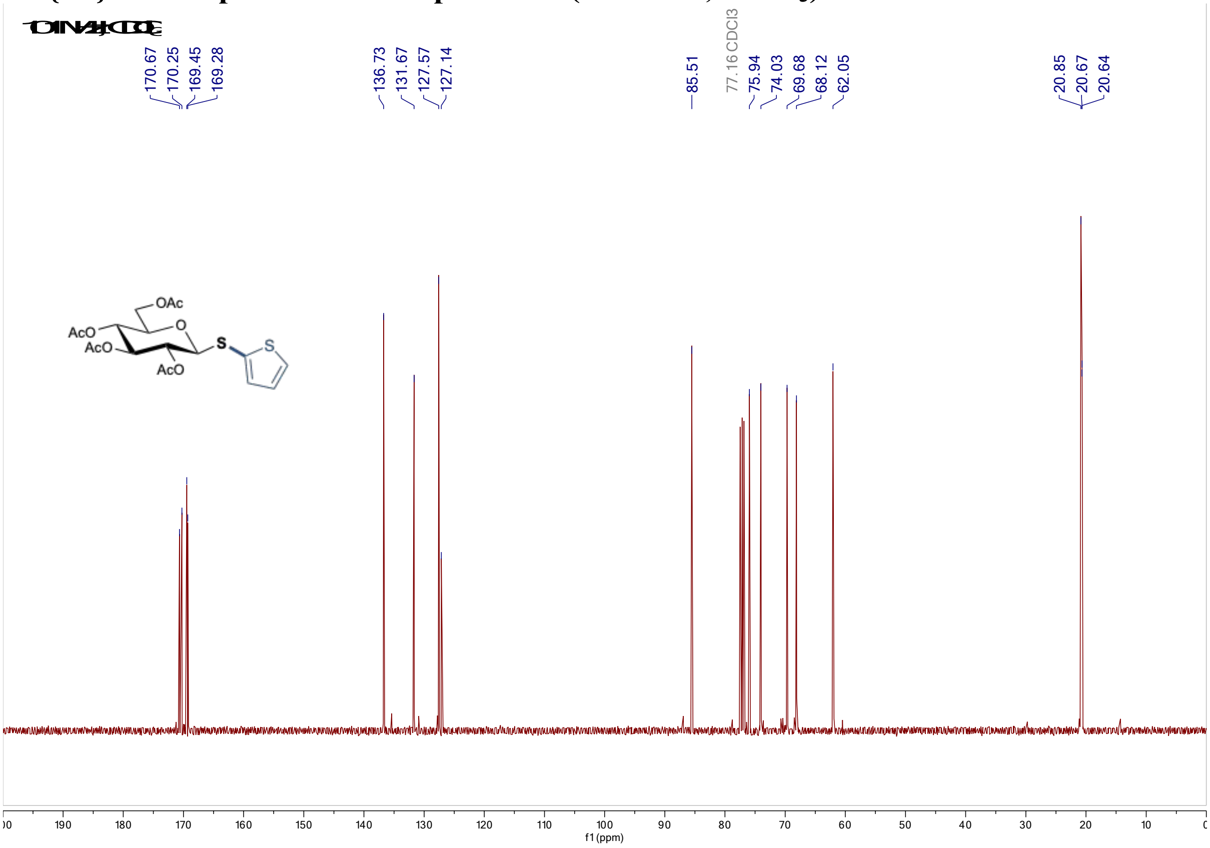

^{1}H NMR Spectrum of compound 18 (500 MHz, $CDCl_3$)

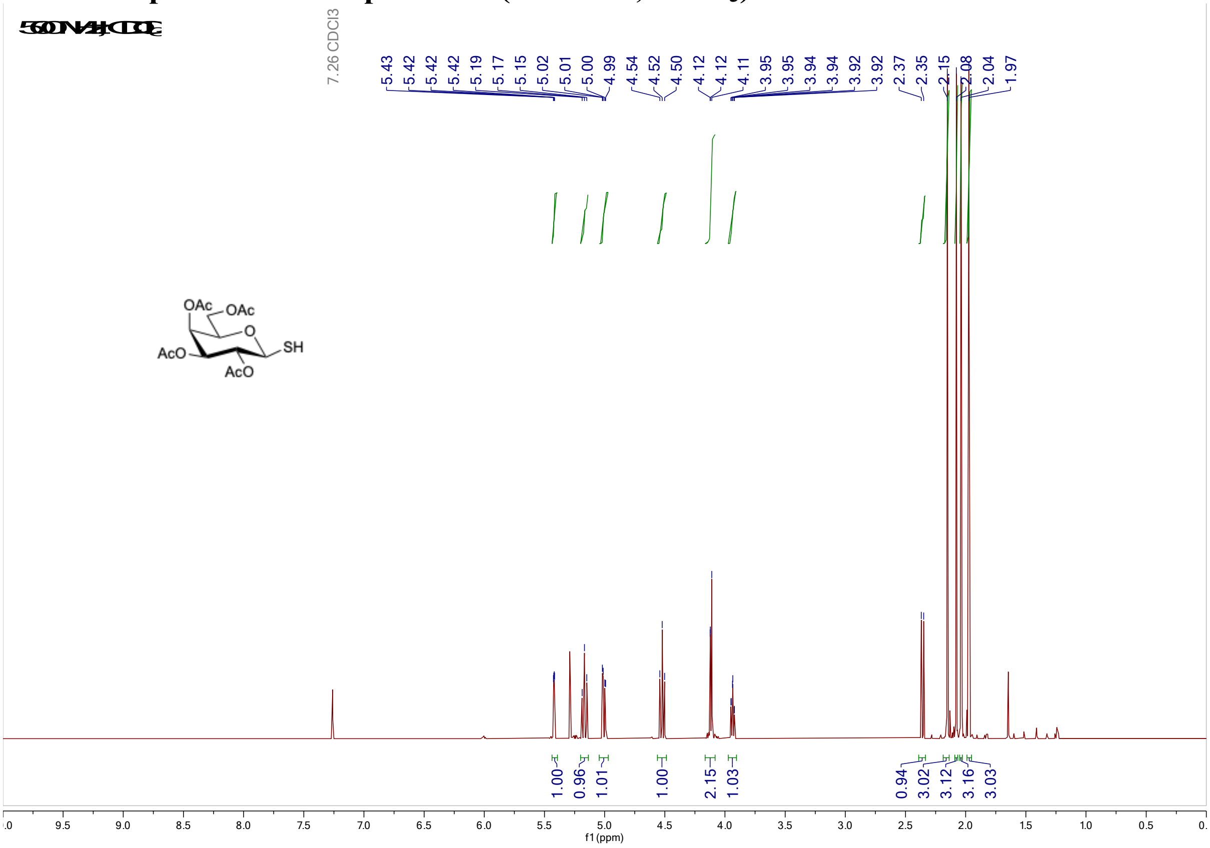

^{13}C{^{1}H} NMR Spectrum of compound 18 (101 MHz, $CDCl_3$)

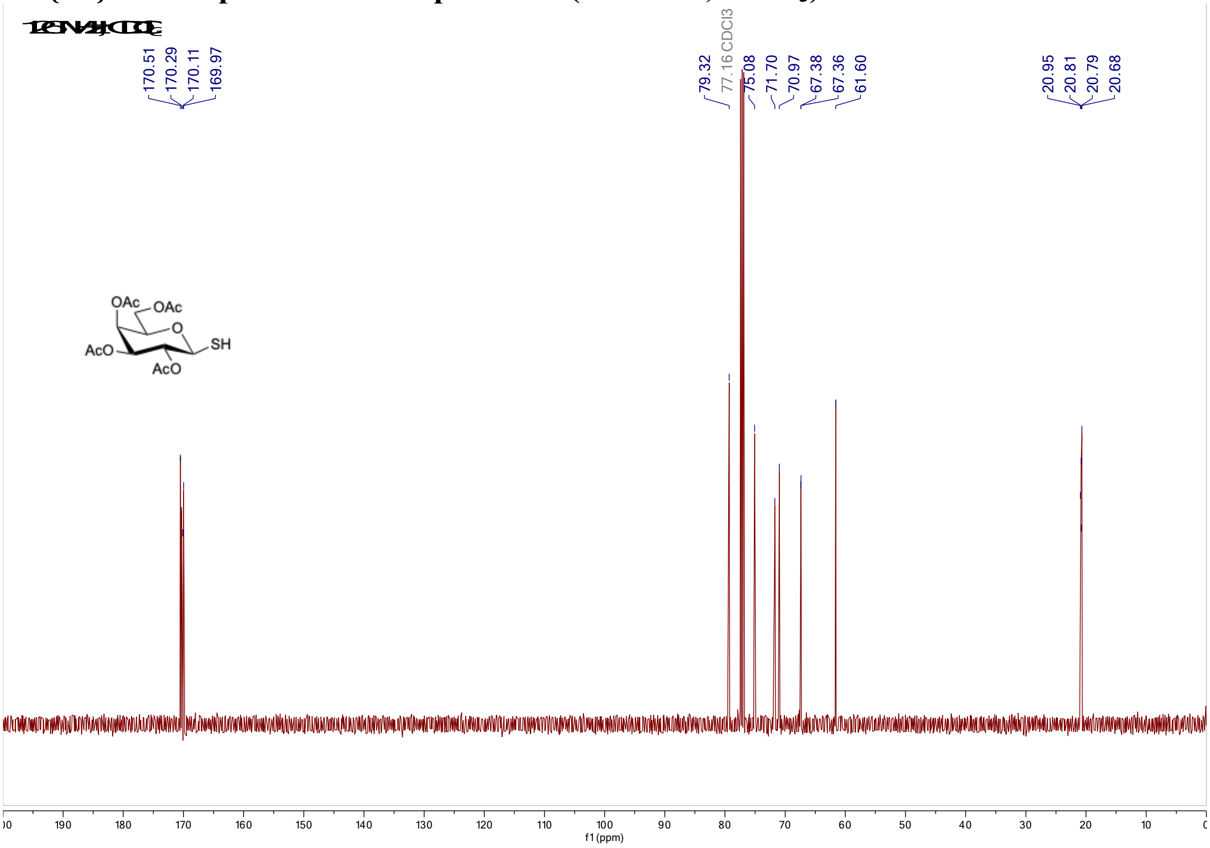

^{1}H NMR Spectrum of compound 19 (400 MHz, $CDCl_3$)

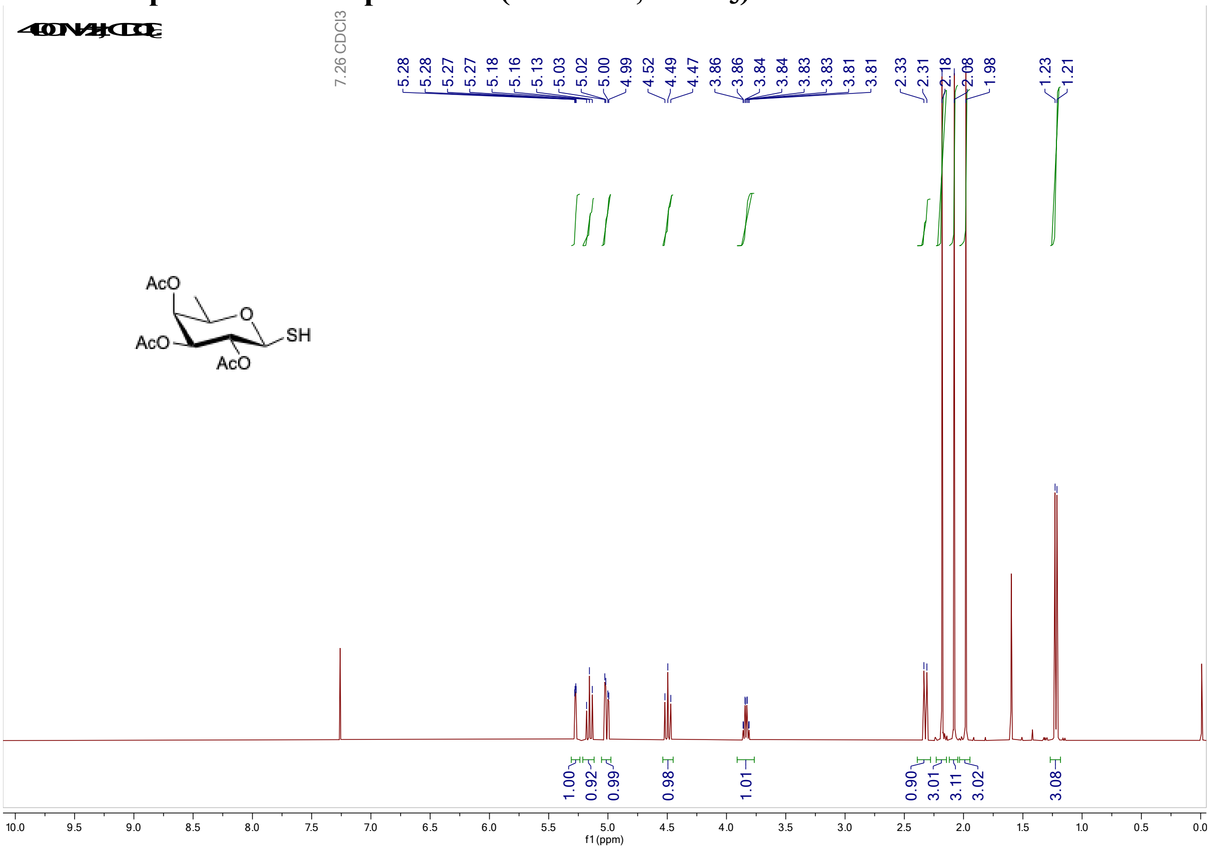

^{13}C{^{1}H} NMR Spectrum of compound 19 (101 MHz, $CDCl_3$)

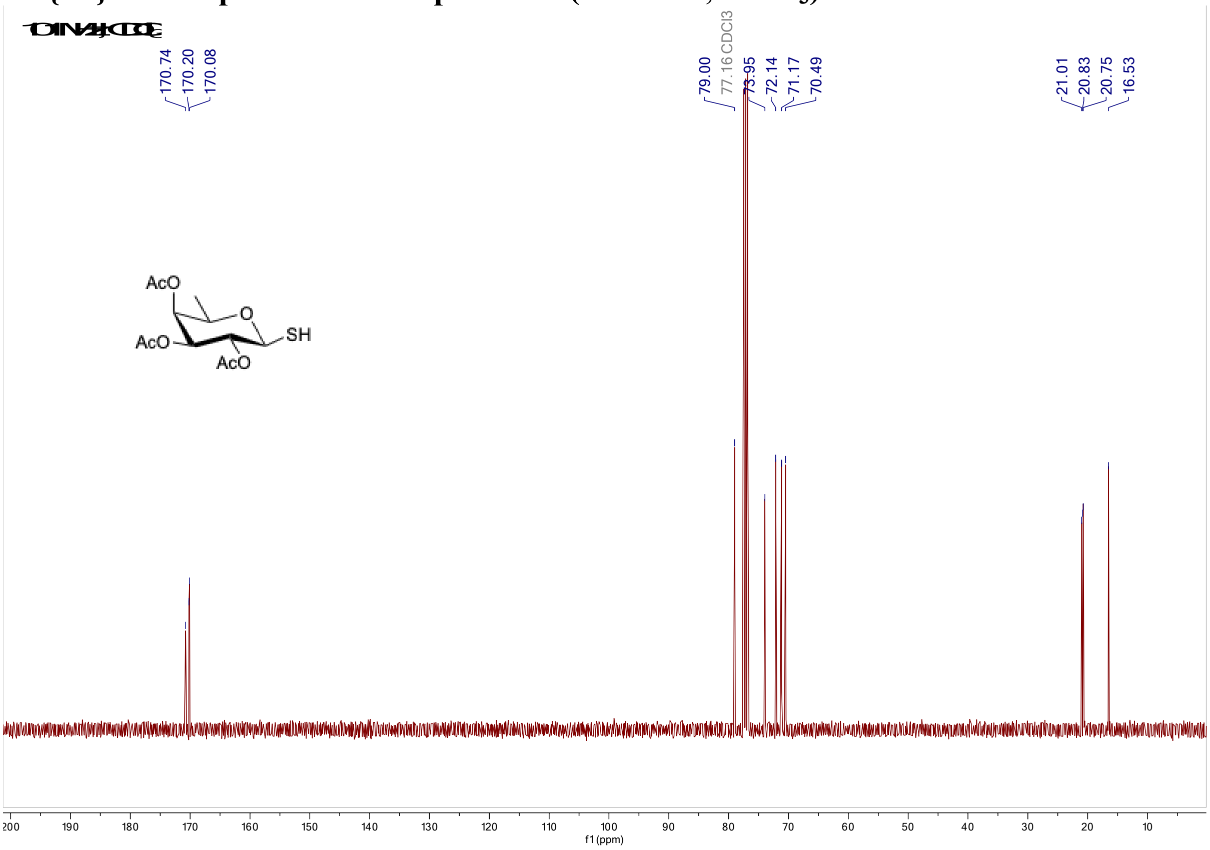

^{1}H NMR Spectrum of compound 20 (400 MHz, $CDCl_3$)

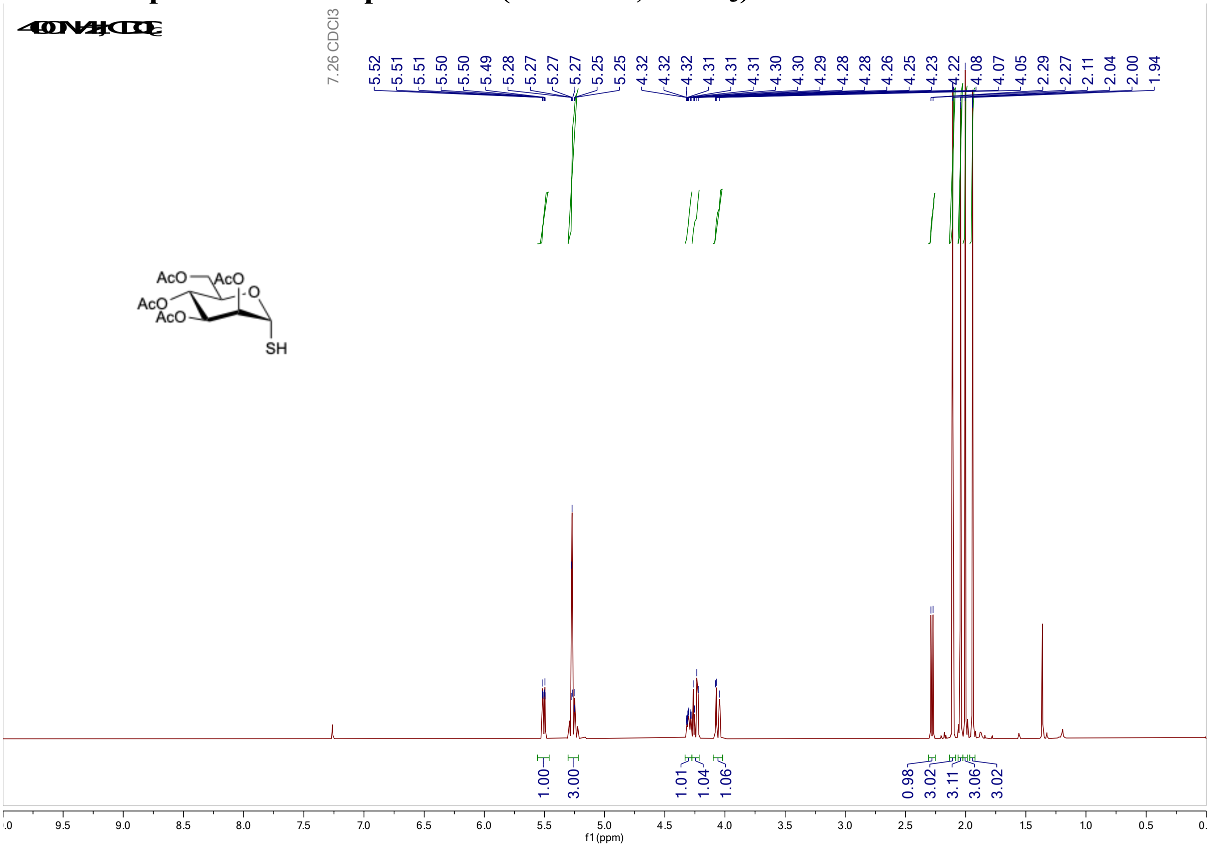

^{1}H NMR Spectrum of compound 21 (400 MHz, $CDCl_3$)

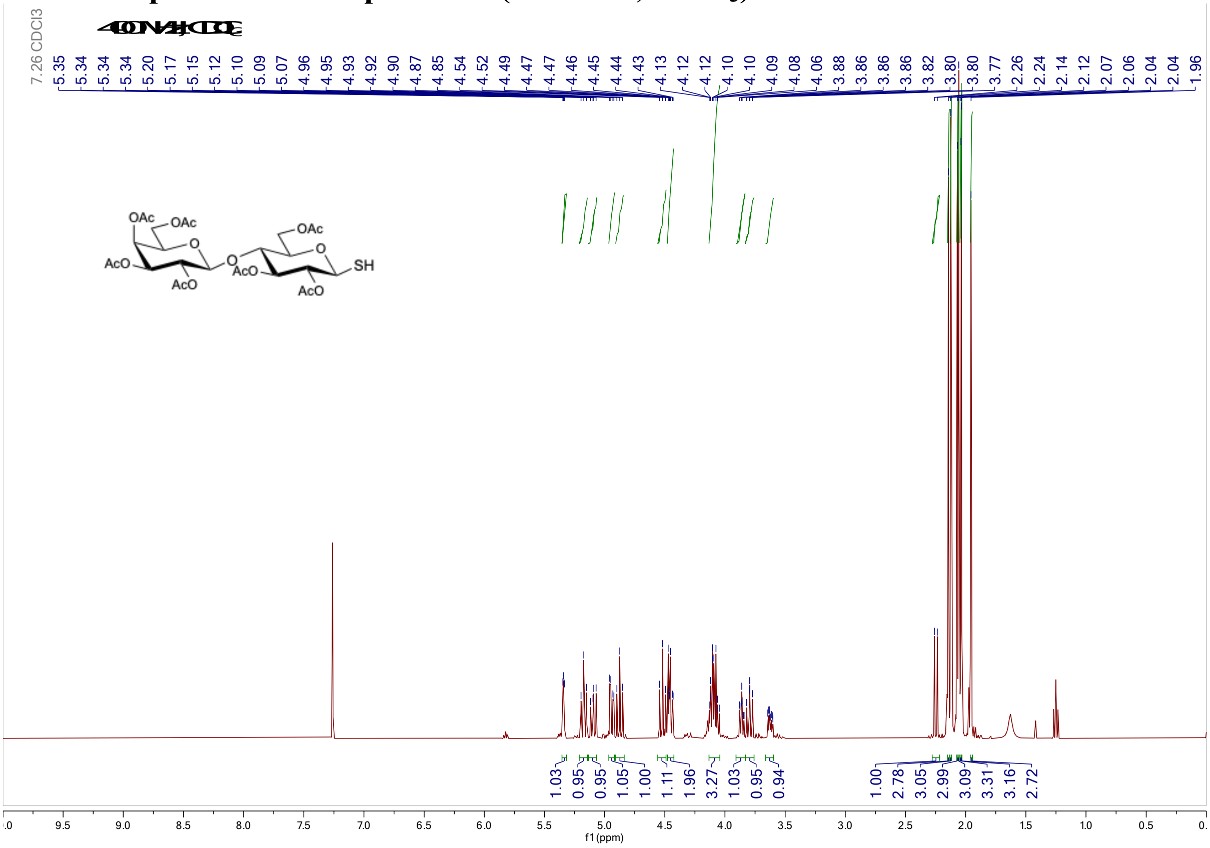

^{13}C{^{1}H} NMR Spectrum of compound 21 (101 MHz, $CDCl_3$)

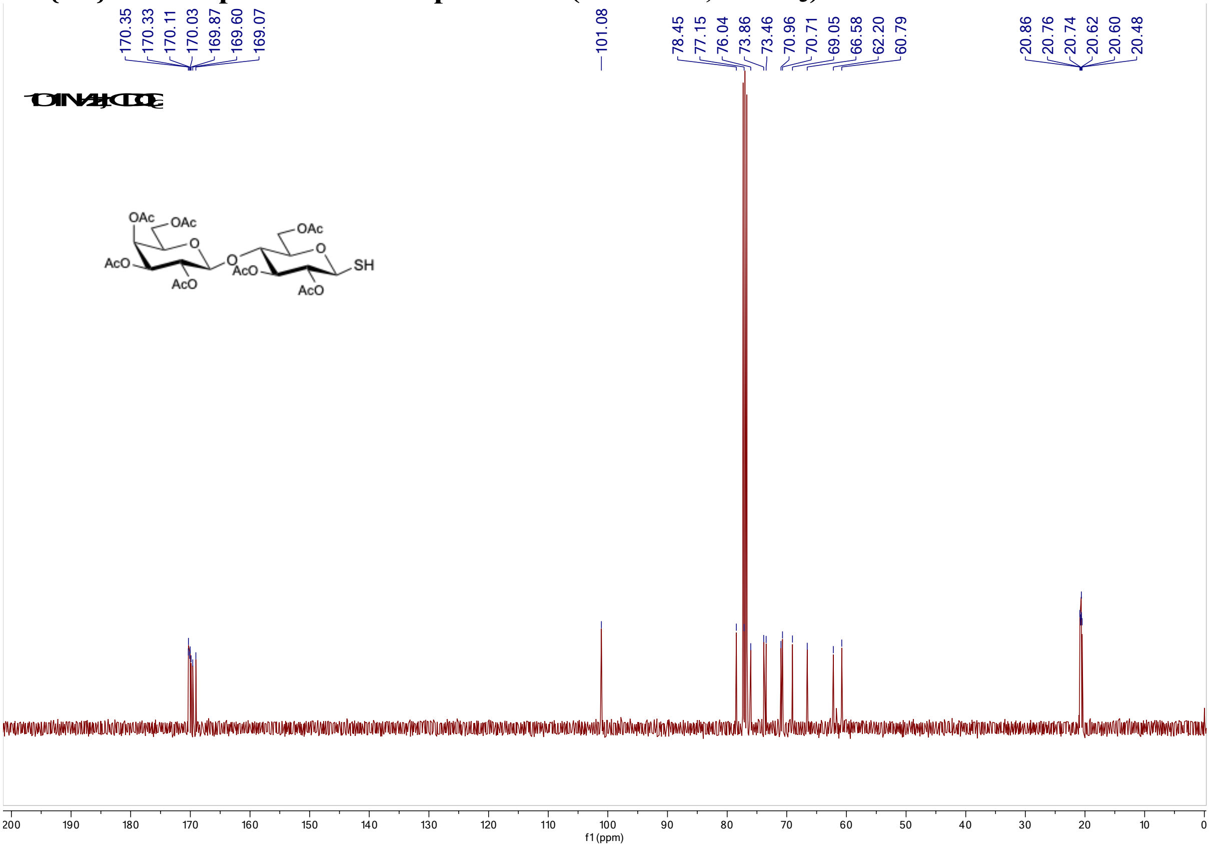

^{1}H NMR Spectrum of compound 22 (400 MHz, $CDCl_3$)

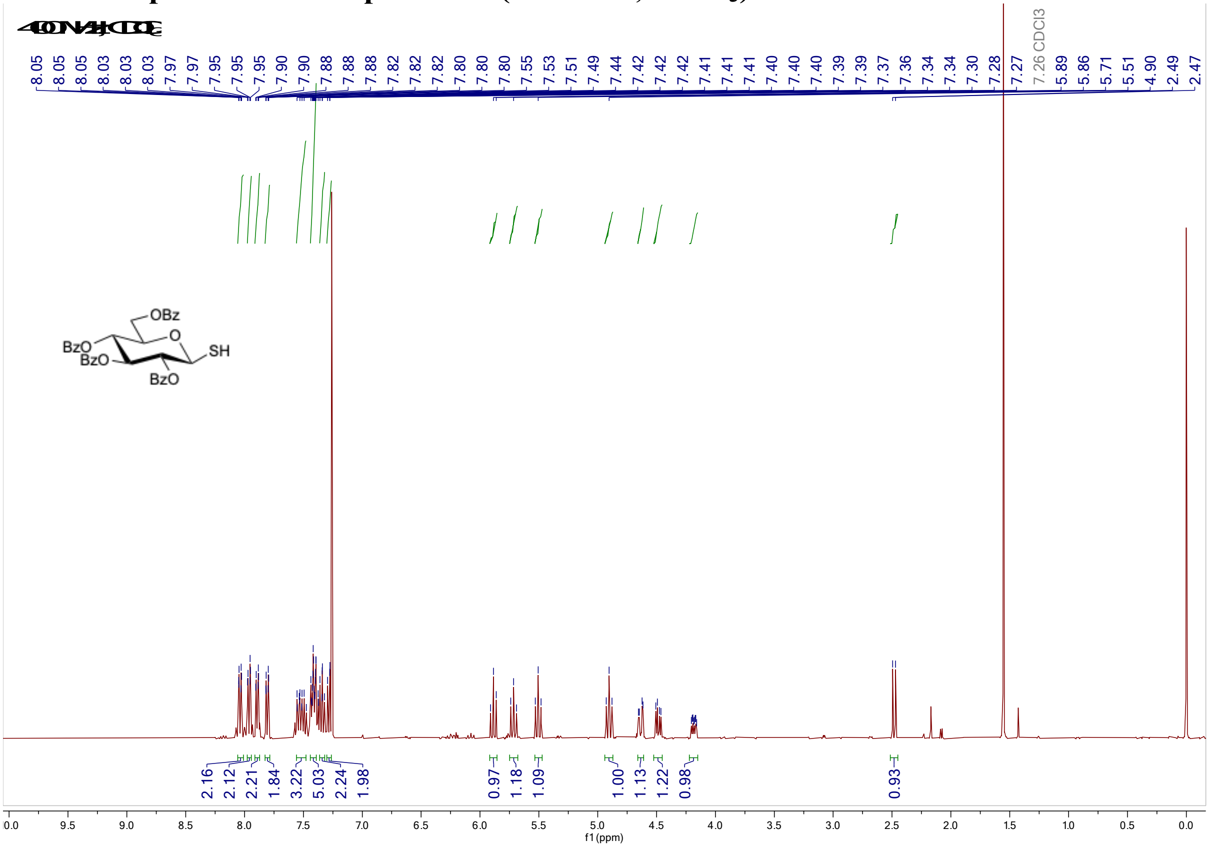

$^{13}C\{^{1}H\}$ NMR Spectrum of compound 22 (101 MHz, $CDCl_3$)

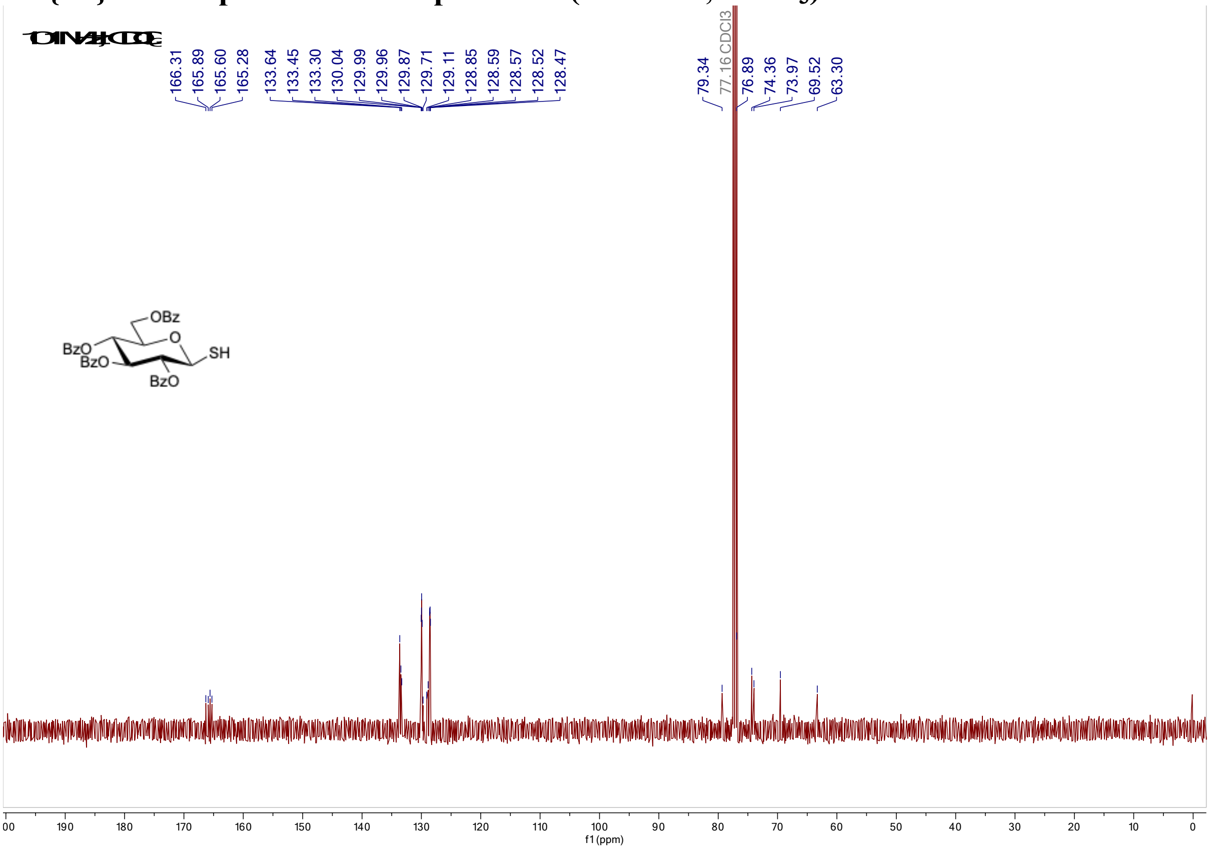

^{1}H NMR Spectrum of compound 23 (400 MHz, $CDCl_3$)

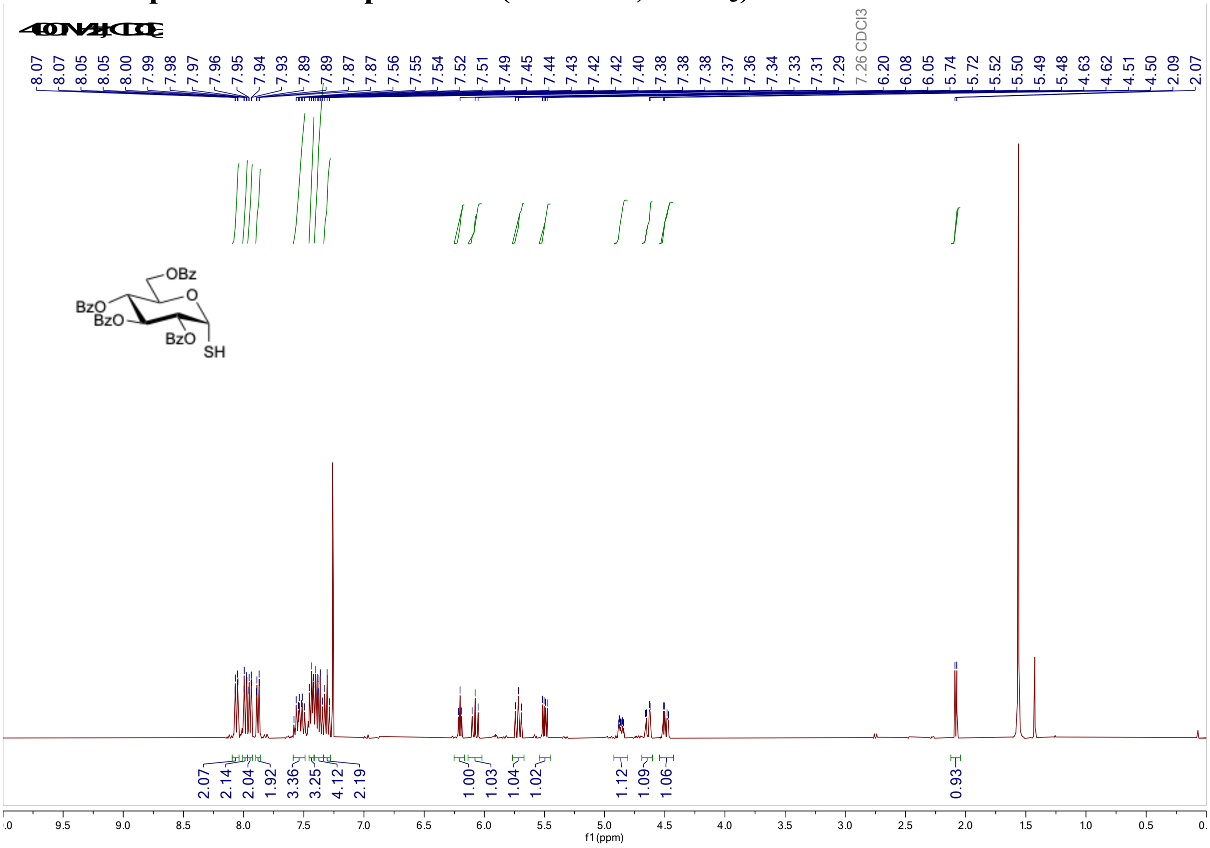

^{1}H NMR Spectrum of compound 24 (400 MHz, $CDCl_3$)

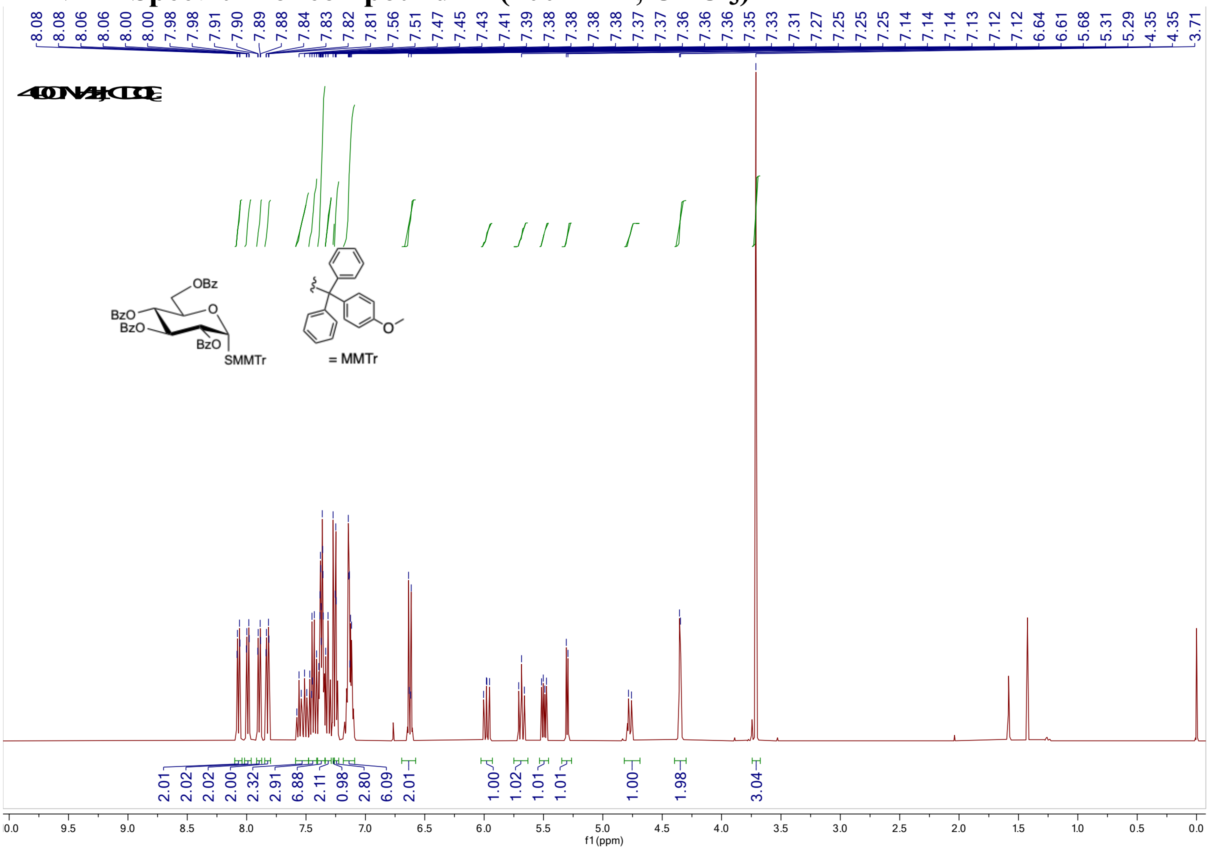

$^{13}C\{^{1}H\}$ NMR Spectrum of compound 24 (101 MHz, $CDCl_3$)

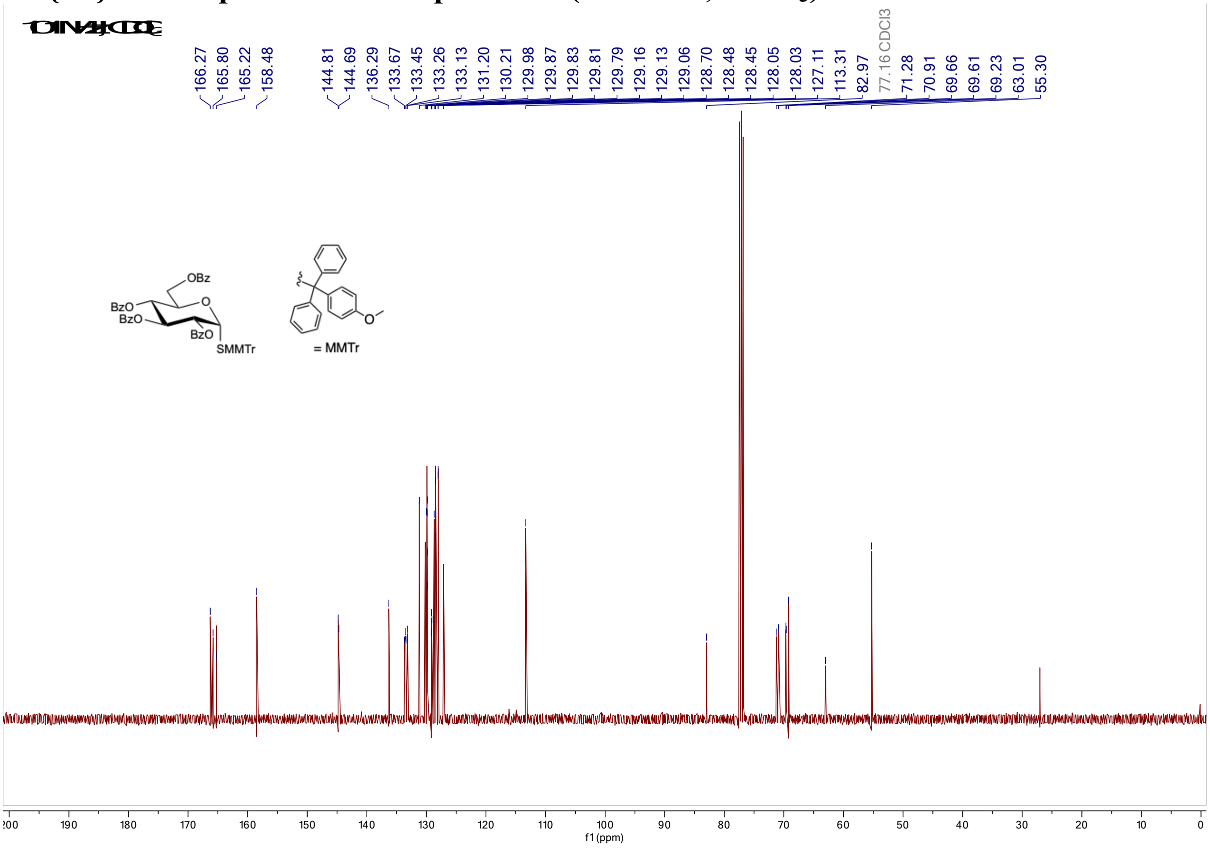

^{1}H NMR Spectrum of compound 25 (400 MHz, $CDCl_3$)

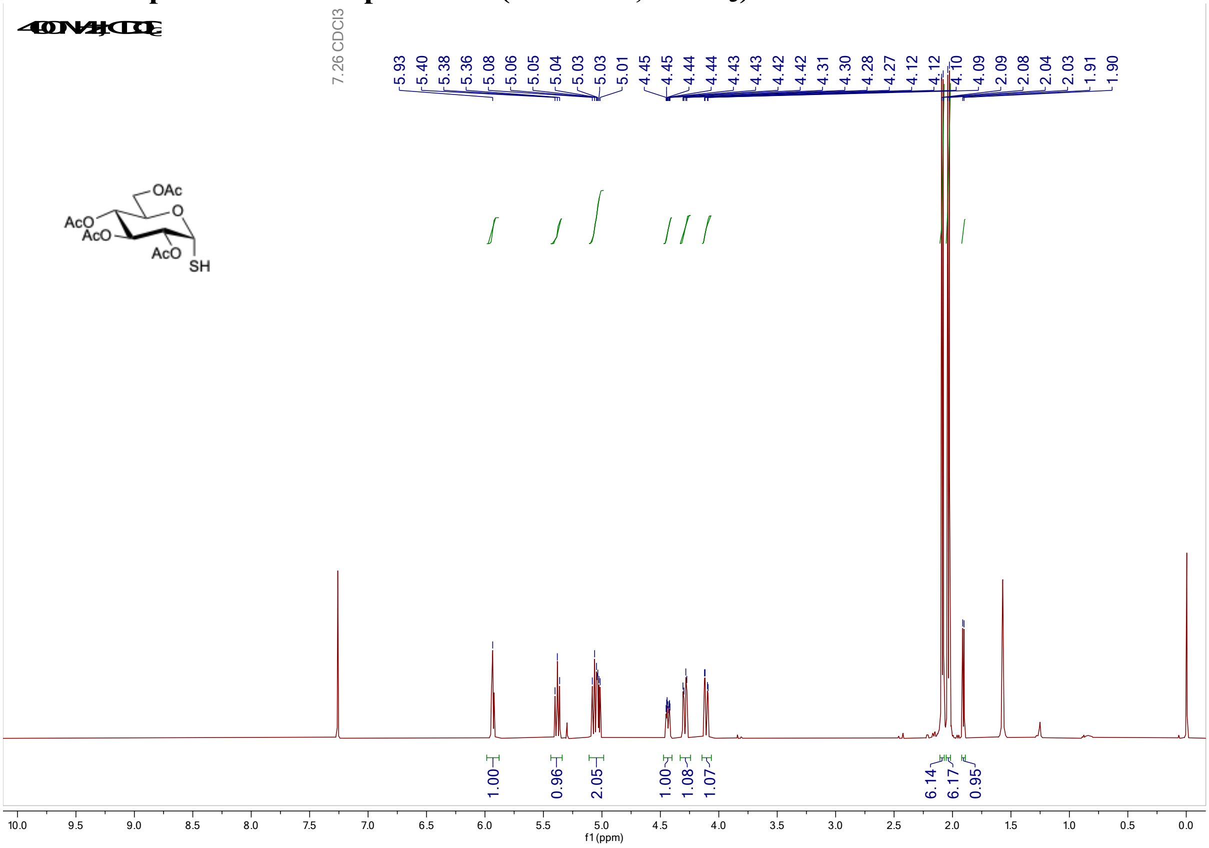

^{13}C{^{1}H} NMR Spectrum of compound 25 (101 MHz, $CDCl_3$)

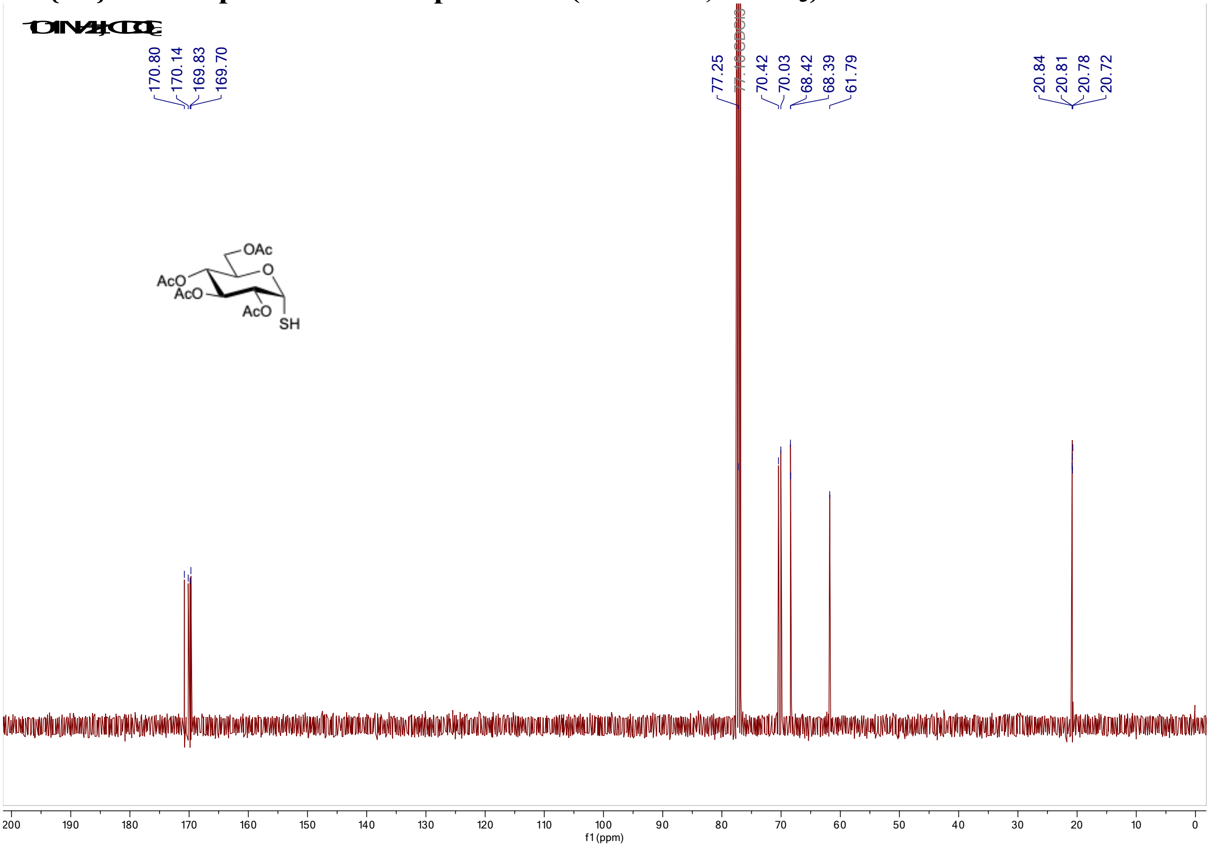

^{1}H NMR Spectrum of compound 26 (500 MHz, $CDCl_3$)

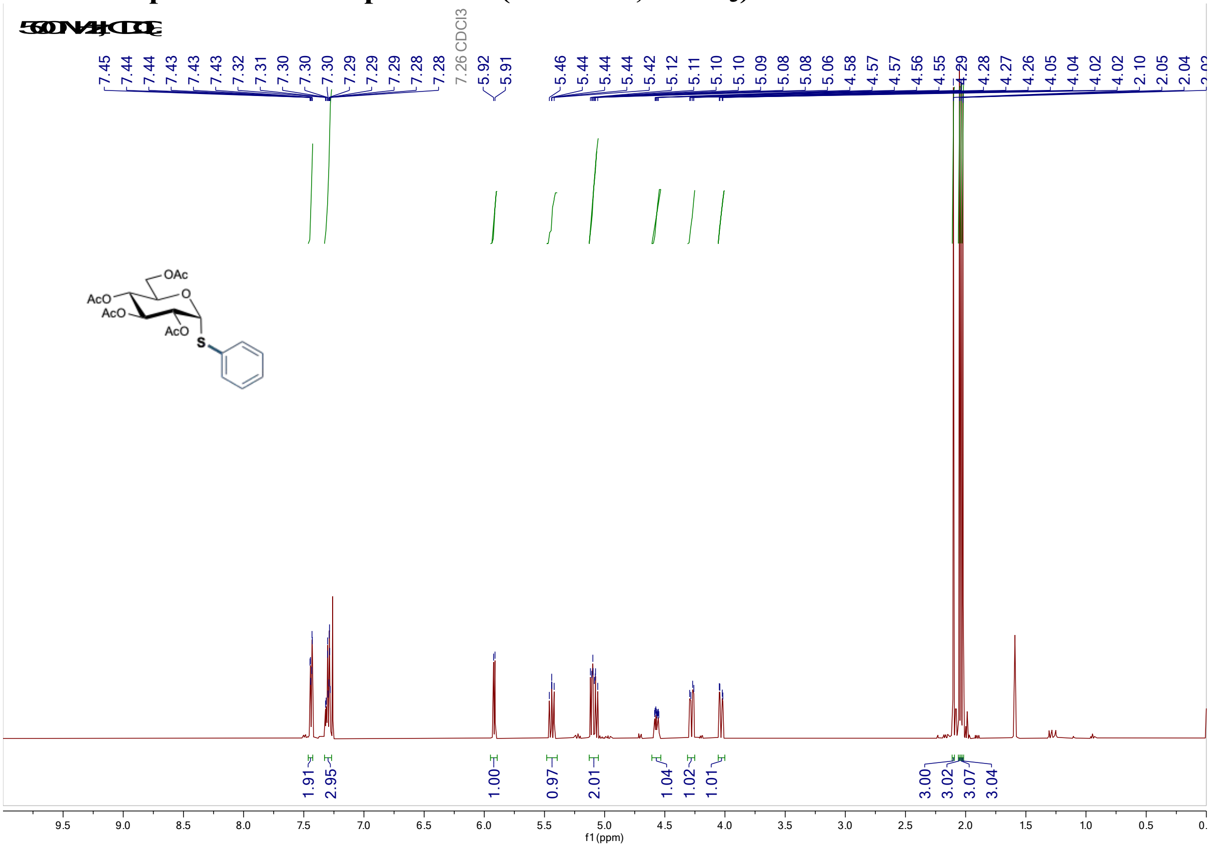

^{13}C{^{1}H} NMR Spectrum of compound 3 (126 MHz, $CDCl_3$)

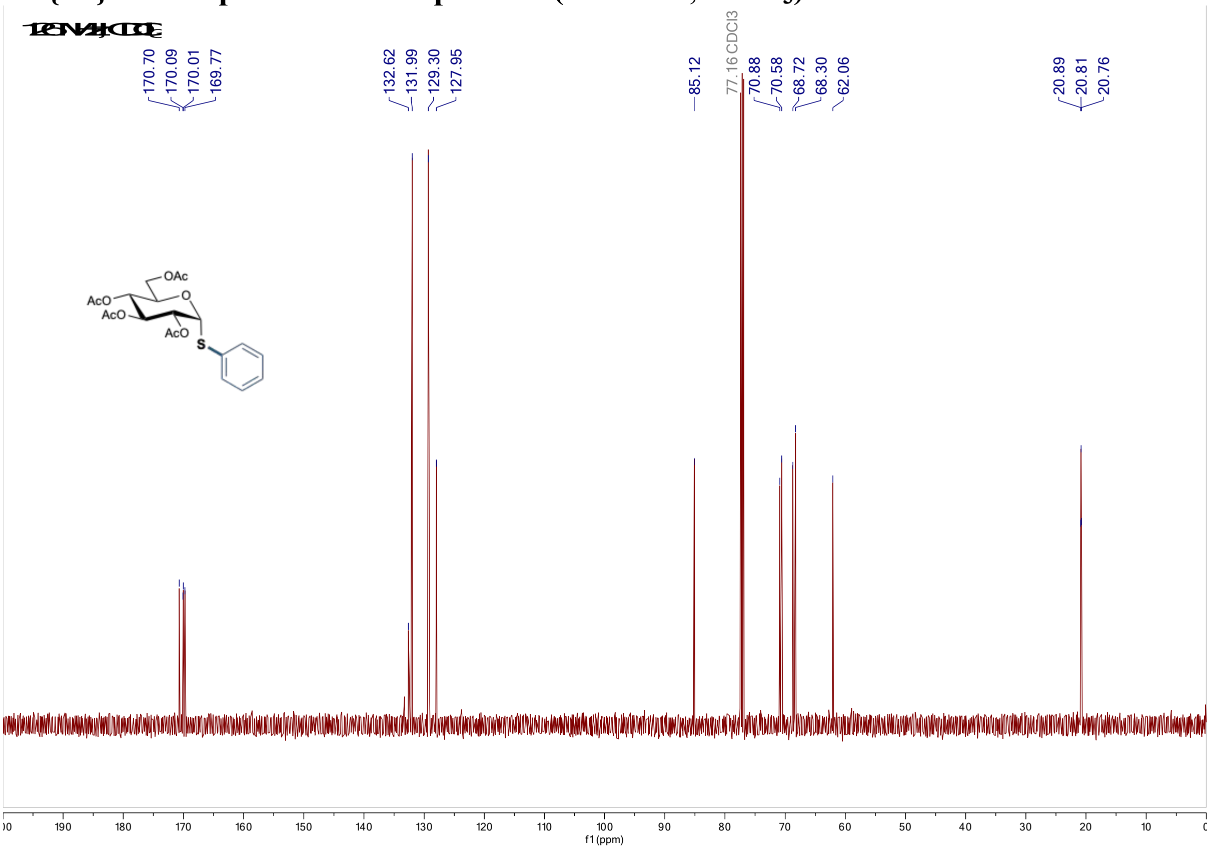

^{1}H NMR Spectrum of compound 27 (400 MHz, $CDCl_3$)

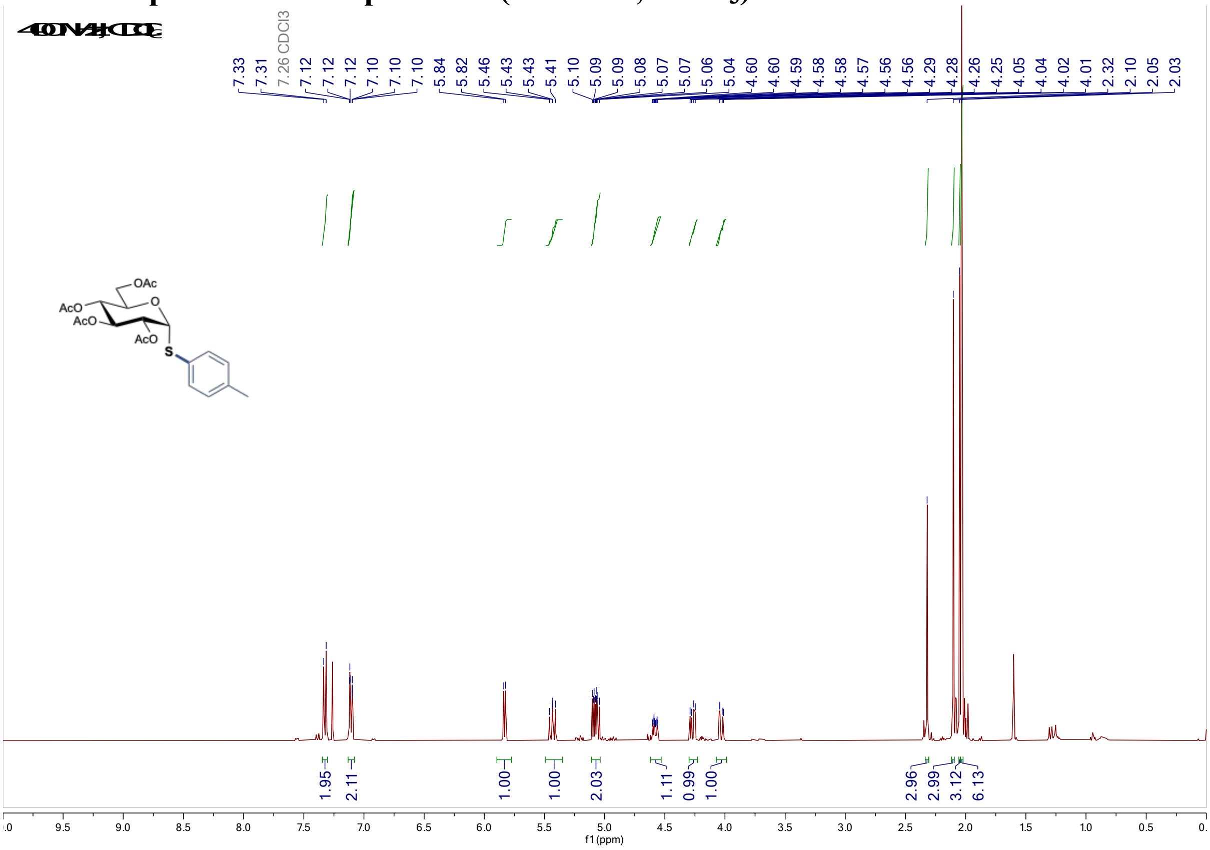

^{13}C{^{1}H} NMR Spectrum of compound 27 (101 MHz, $CDCl_3$)

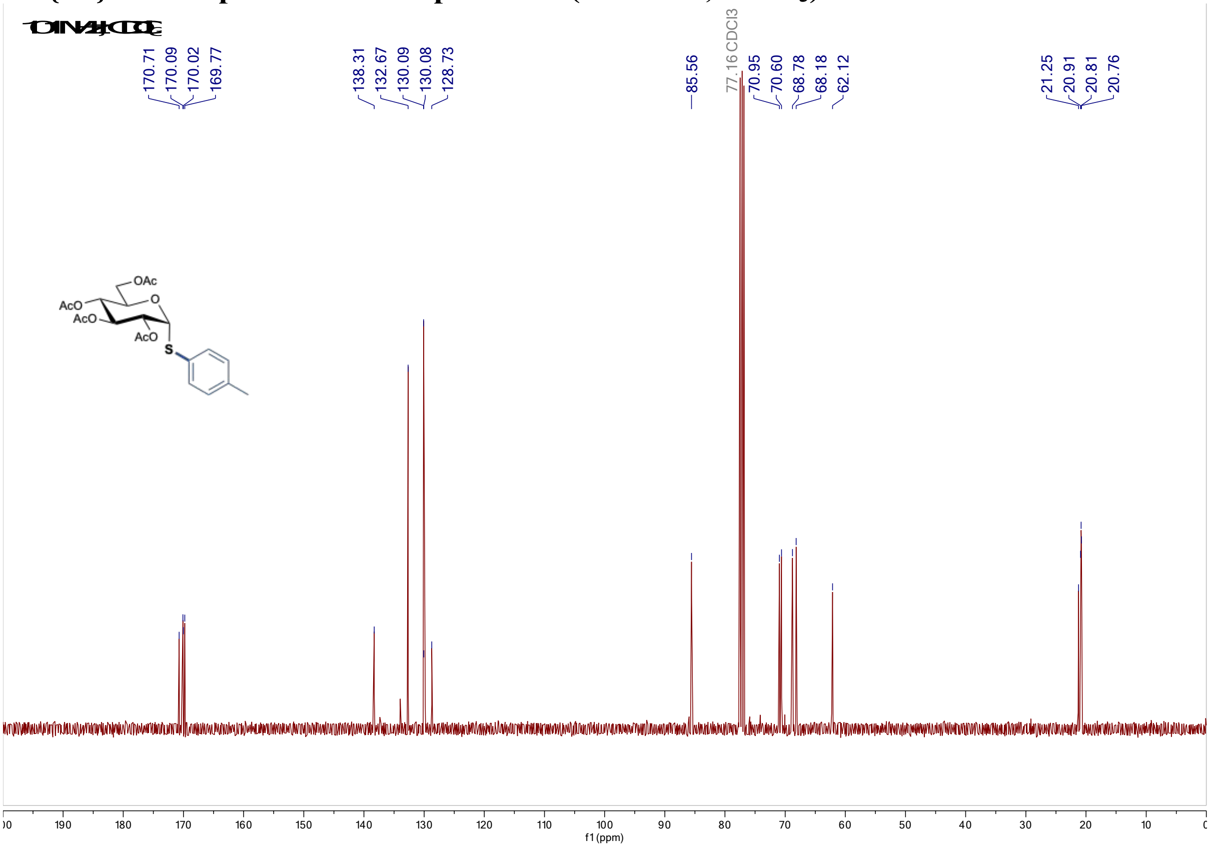

^{1}H NMR Spectrum of compound 28 (500 MHz, $CDCl_3$)

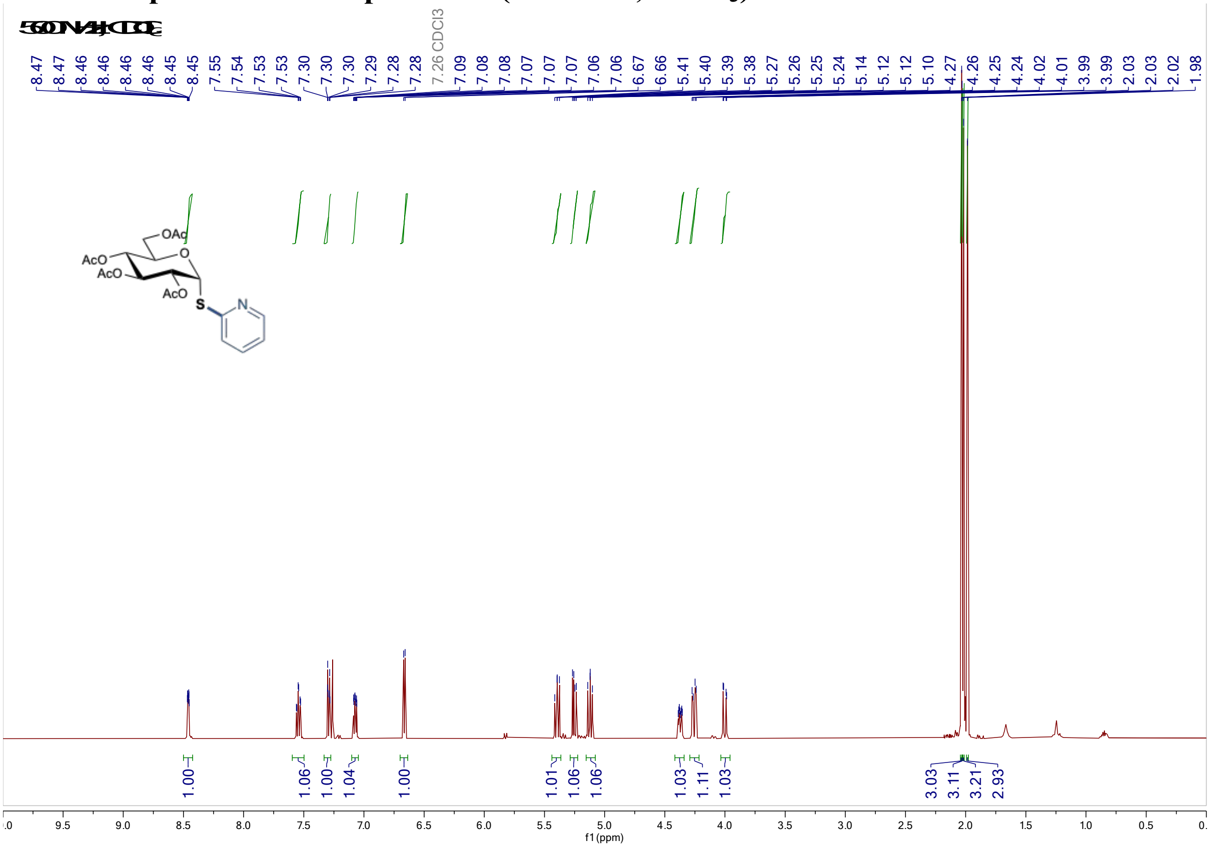

^{13}C{^{1}H} NMR Spectrum of compound 28 (126 MHz, $CDCl_3$)

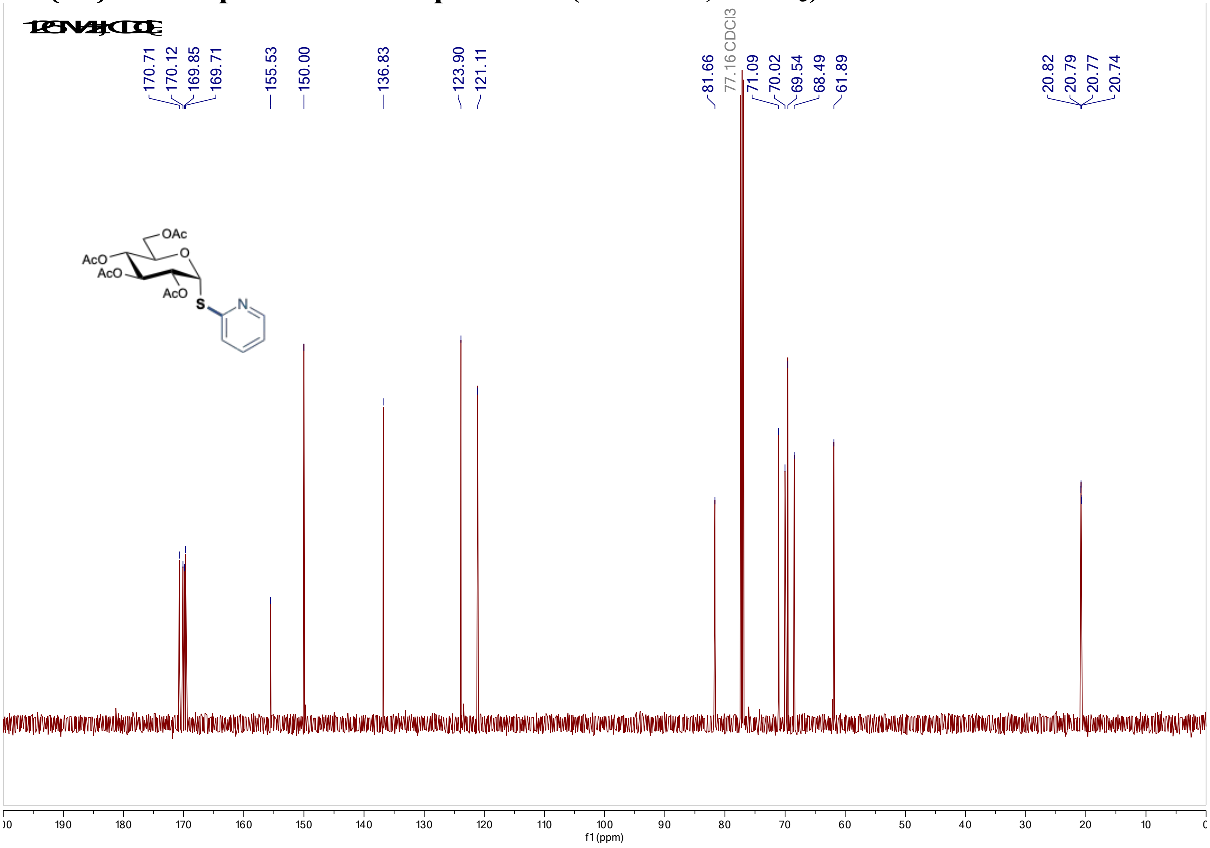

^{1}H NMR Spectrum of compound 29 (400 MHz, $CDCl_3$)

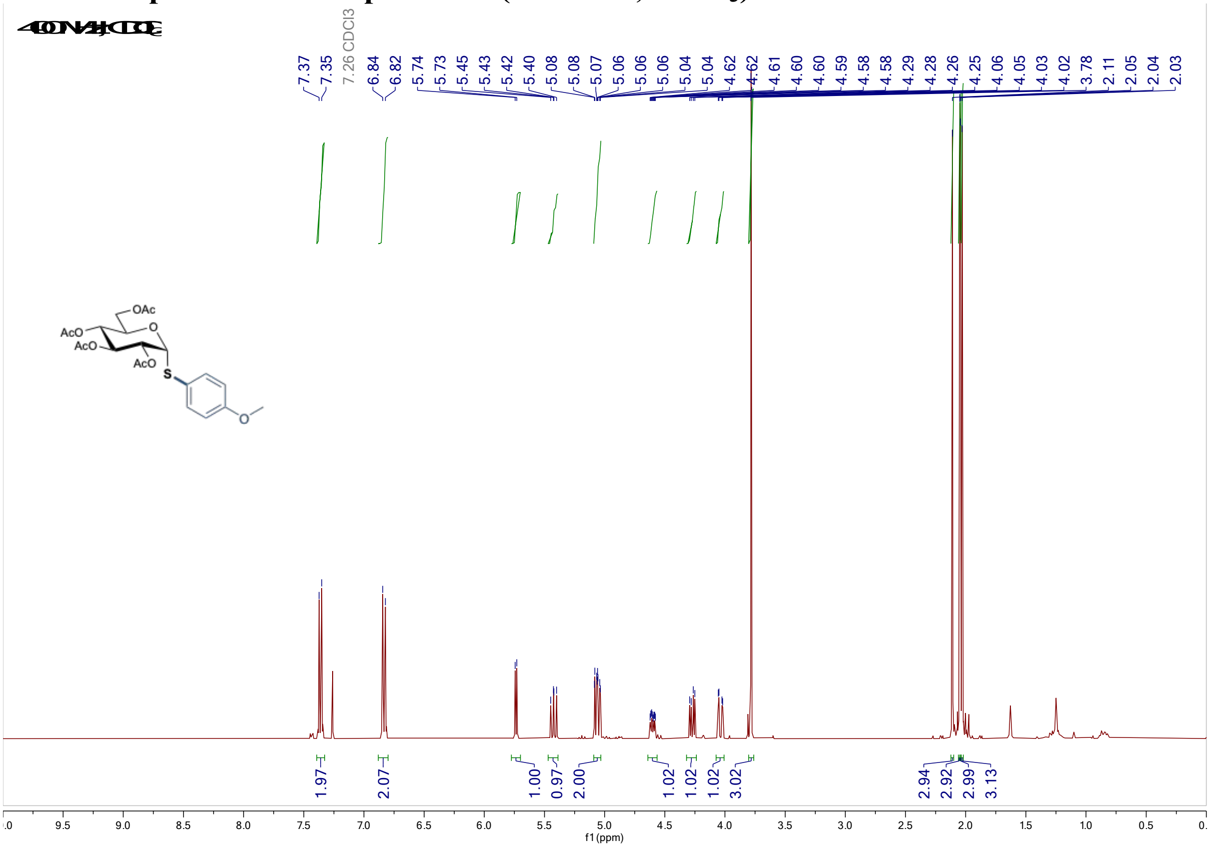

^{13}C{^{1}H} NMR Spectrum of compound 29 (101 MHz, $CDCl_3$)

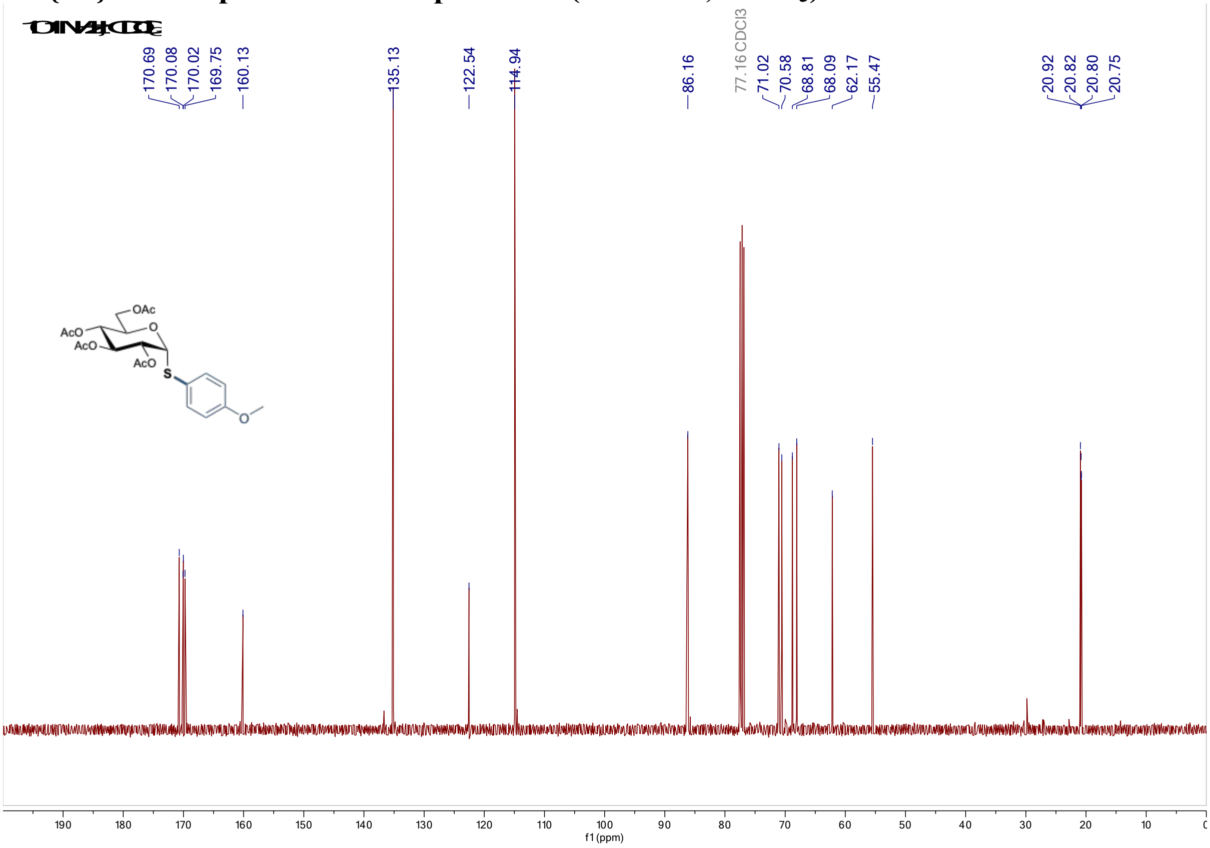

^{1}H NMR Spectrum of compound 30 (500 MHz, $CDCl_3$)

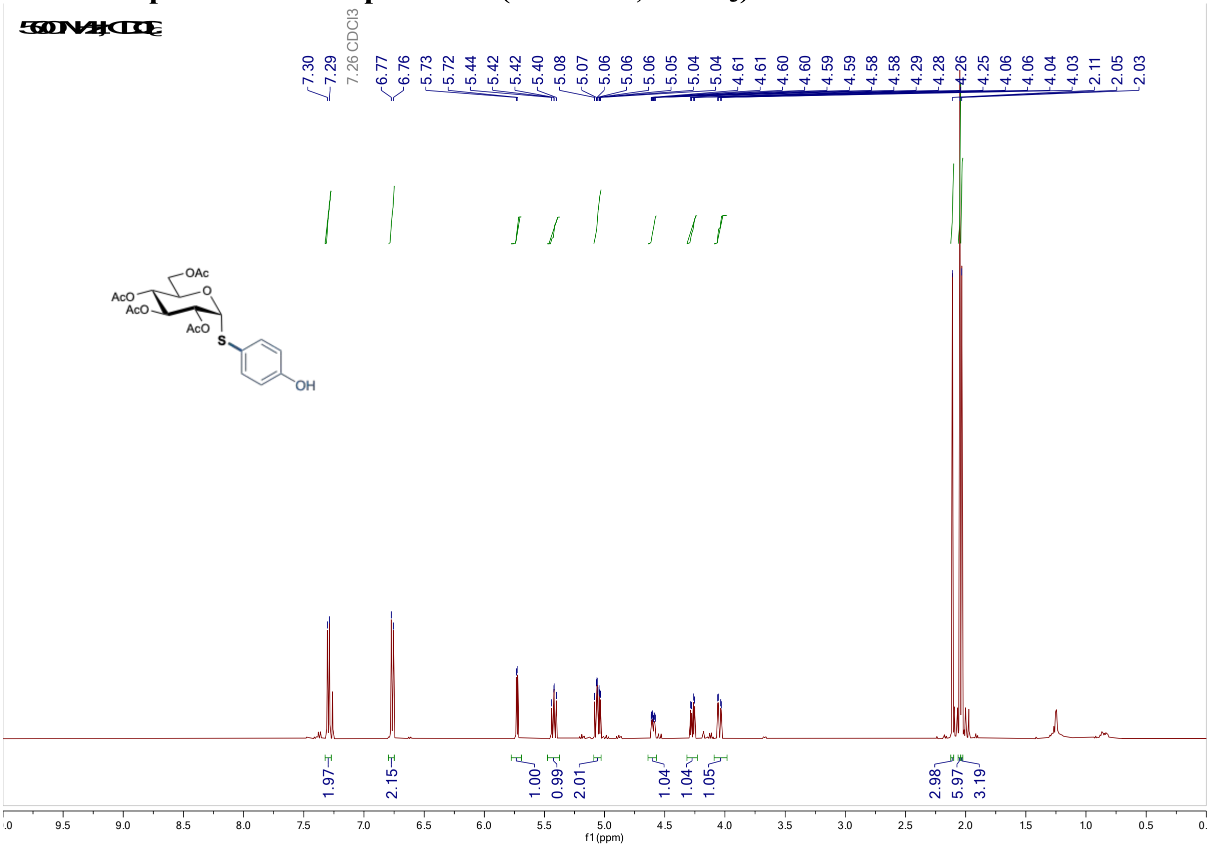

$^{13}C\{^{1}H\}$ NMR Spectrum of compound 30 (126 MHz, $CDCl_3$)

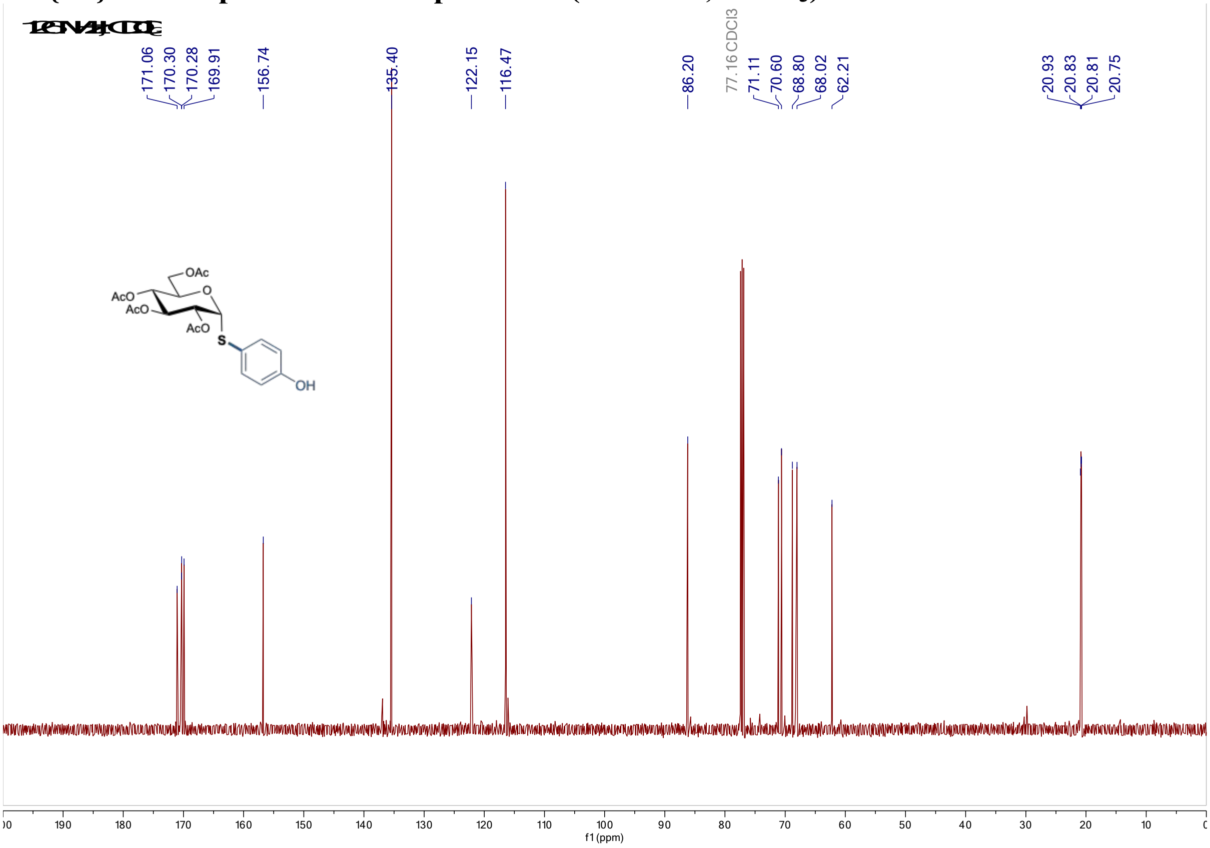

^{1}H NMR Spectrum of compound 31 (400 MHz, $CDCl_3$)

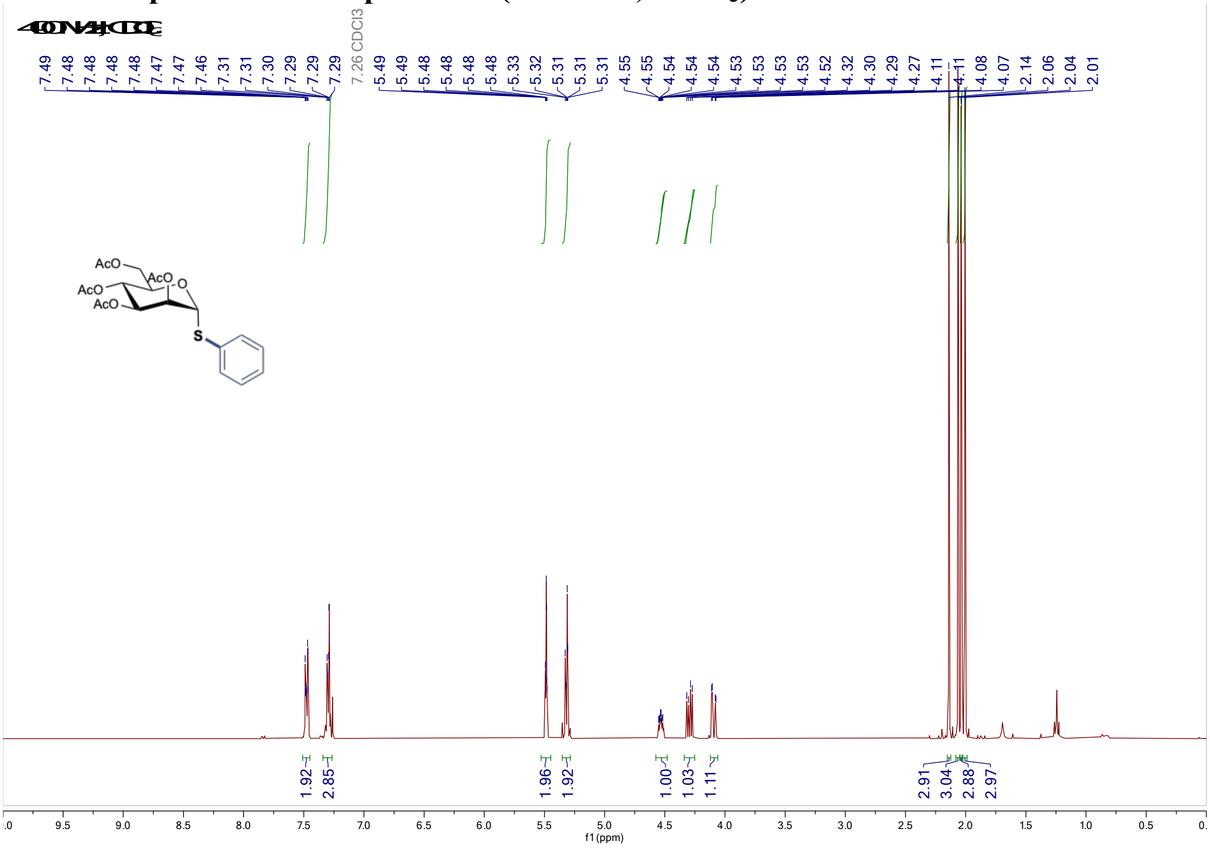

$^{13}C\{^1H\}$ NMR Spectrum of compound 31 (101 MHz, $CDCl_3$)

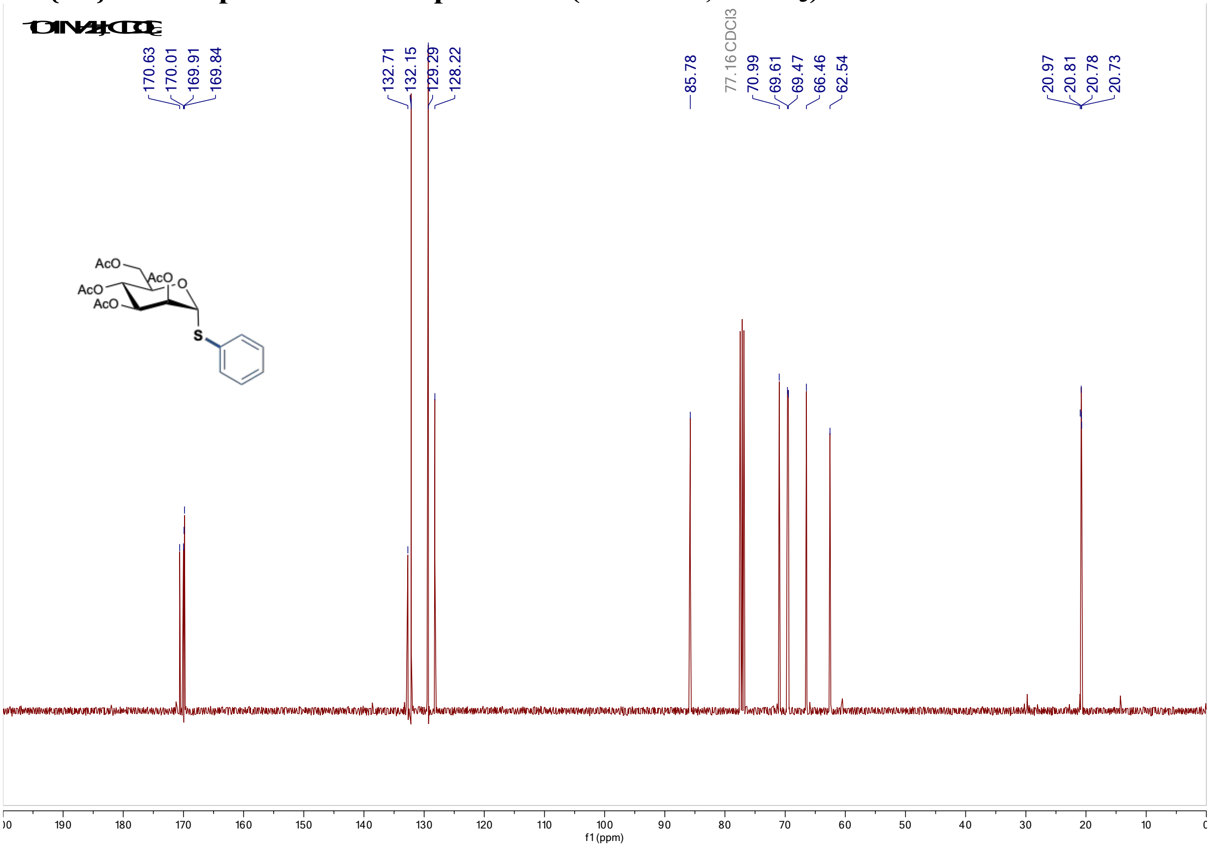

^{1}H NMR Spectrum of compound 32 (400 MHz, $CDCl_3$)

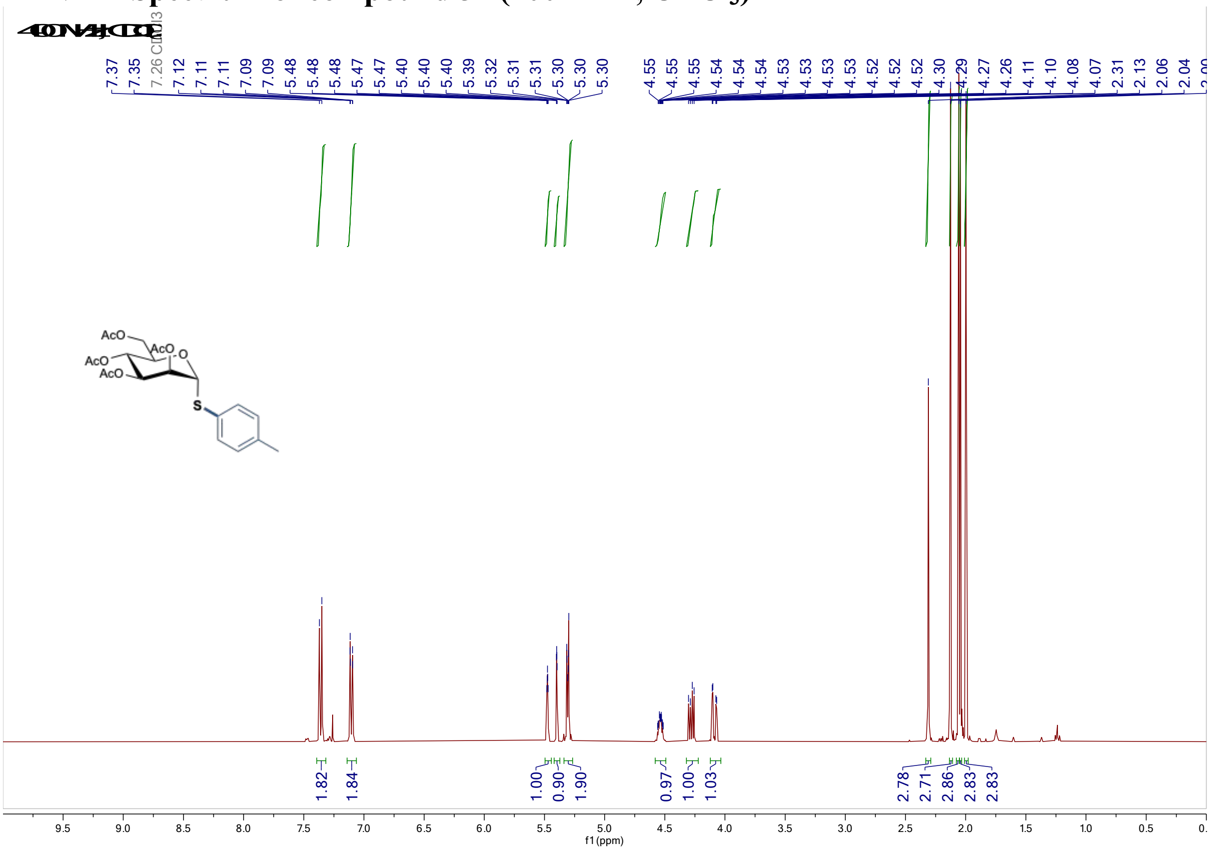

^{13}C{^{1}H} NMR Spectrum of compound 32 (101 MHz, $CDCl_3$)

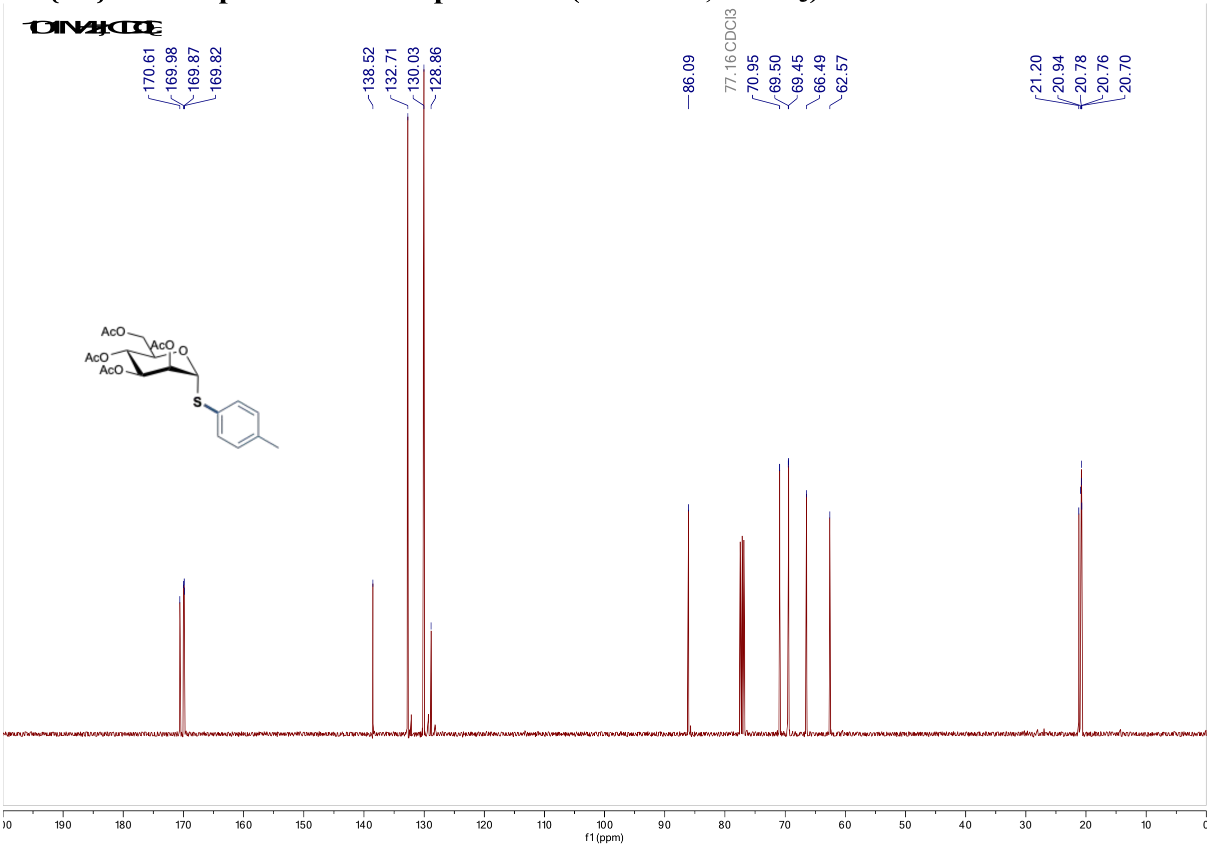

^{1}H NMR Spectrum of compound 33 (400 MHz, $CDCl_3$)

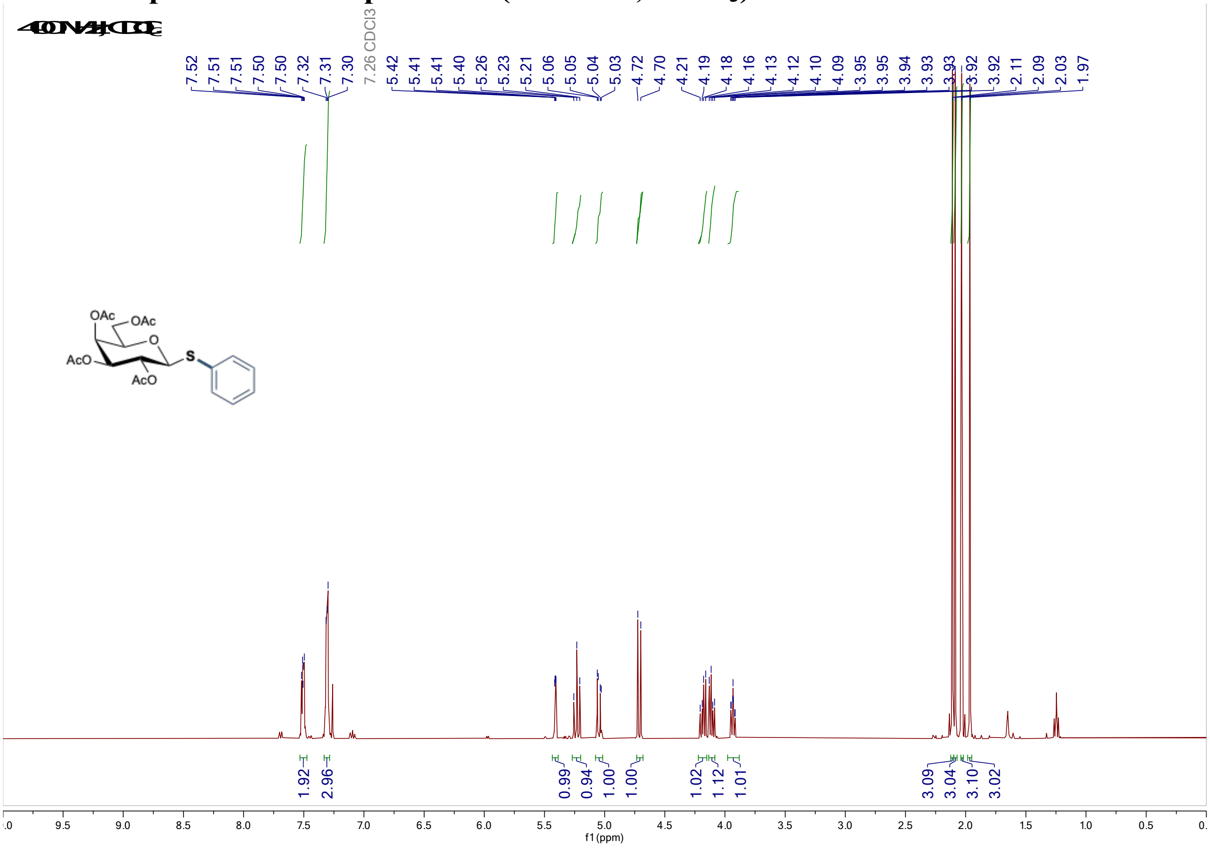

^{13}C{^{1}H} NMR Spectrum of compound 33 (101 MHz, $CDCl_3$)

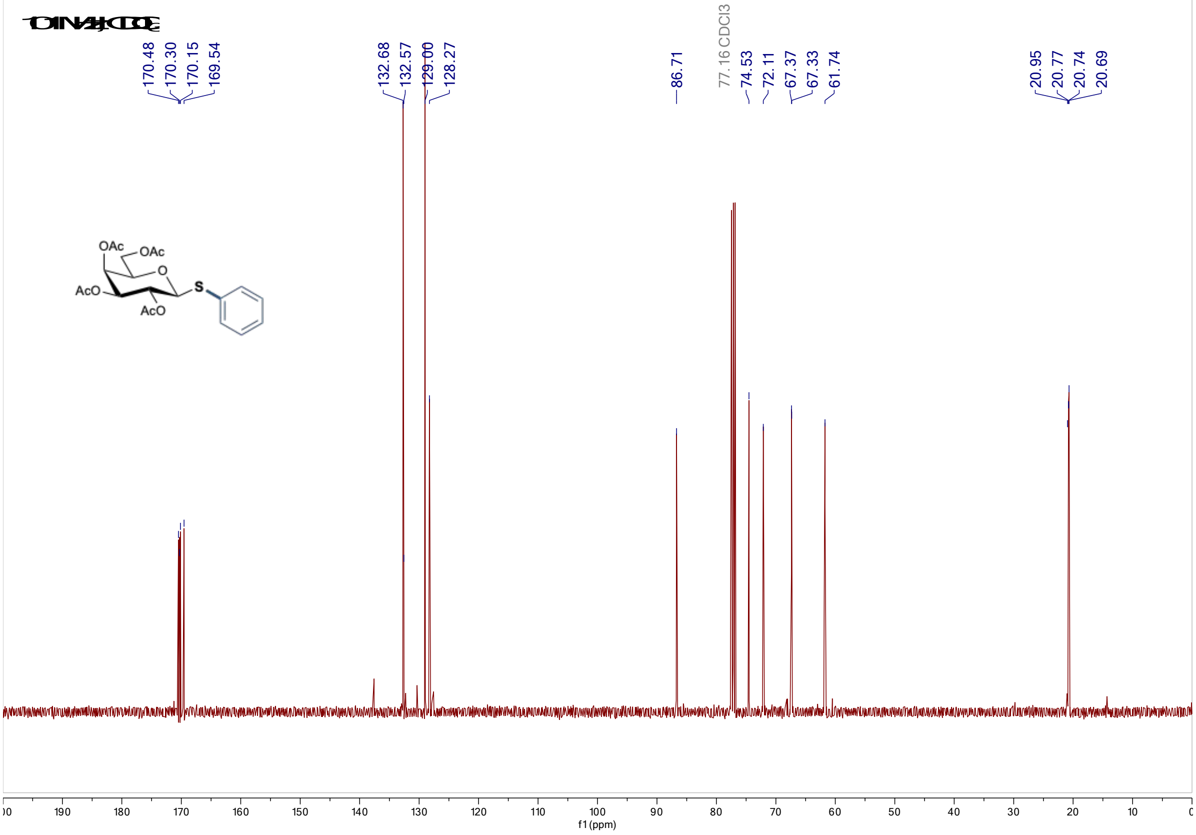

^{1}H NMR Spectrum of compound 34 (400 MHz, $CDCl_3$)

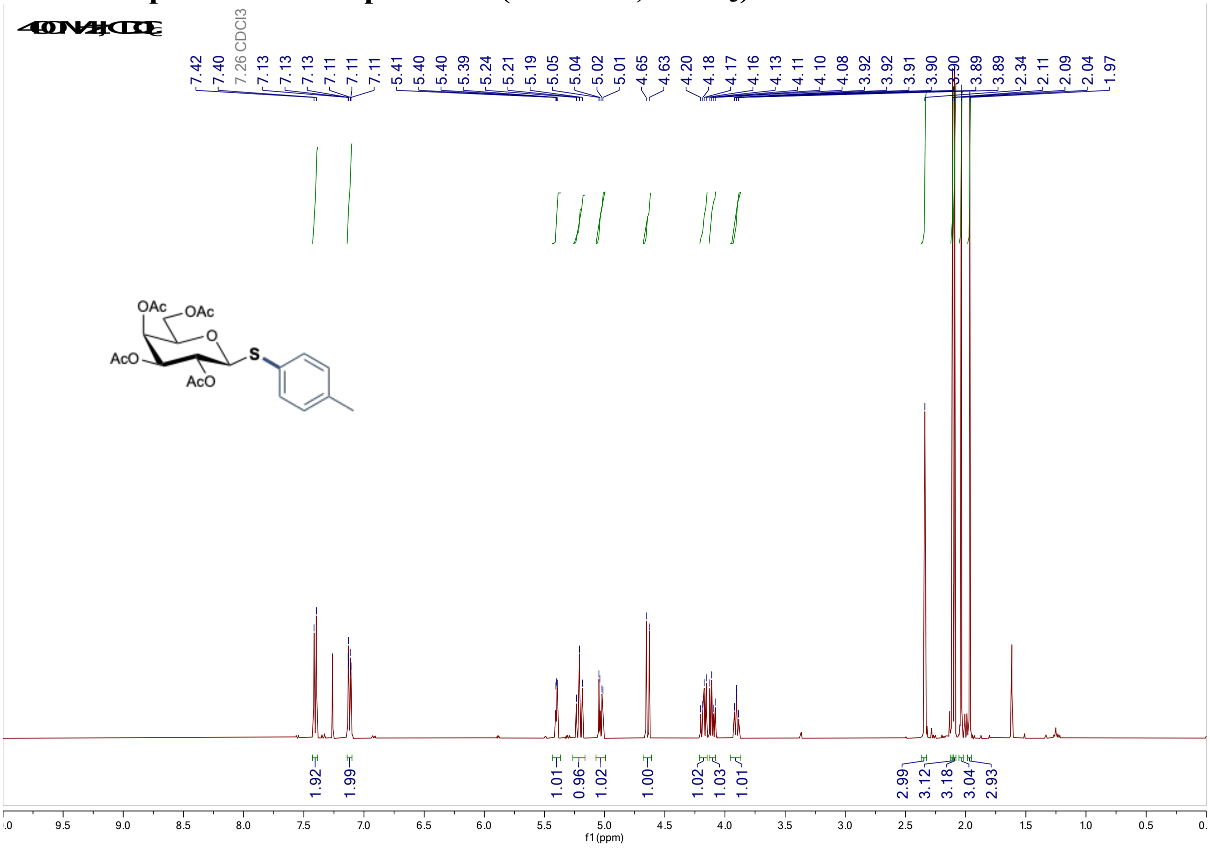

^{13}C{^{1}H} NMR Spectrum of compound 34 (101 MHz, $CDCl_3$)

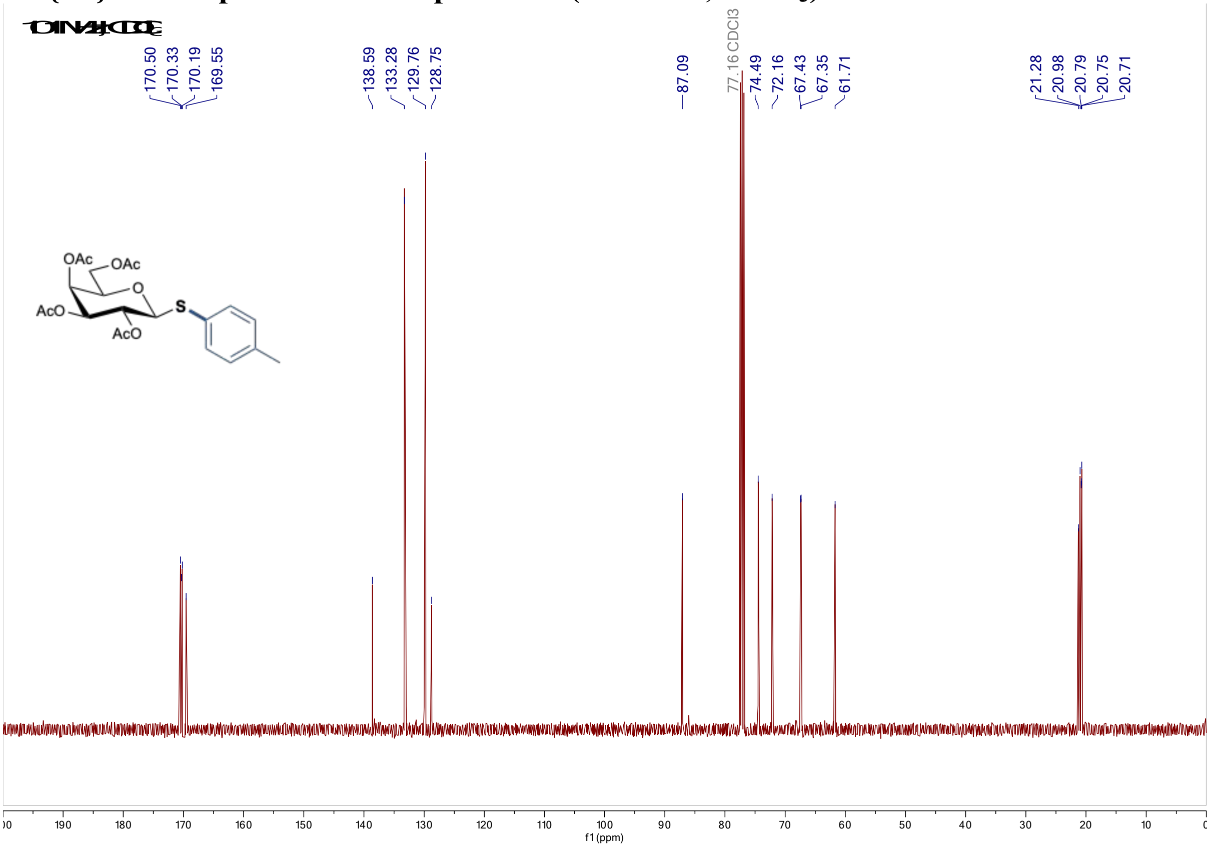

^{1}H NMR Spectrum of compound 35 (400 MHz, $CDCl_3$)

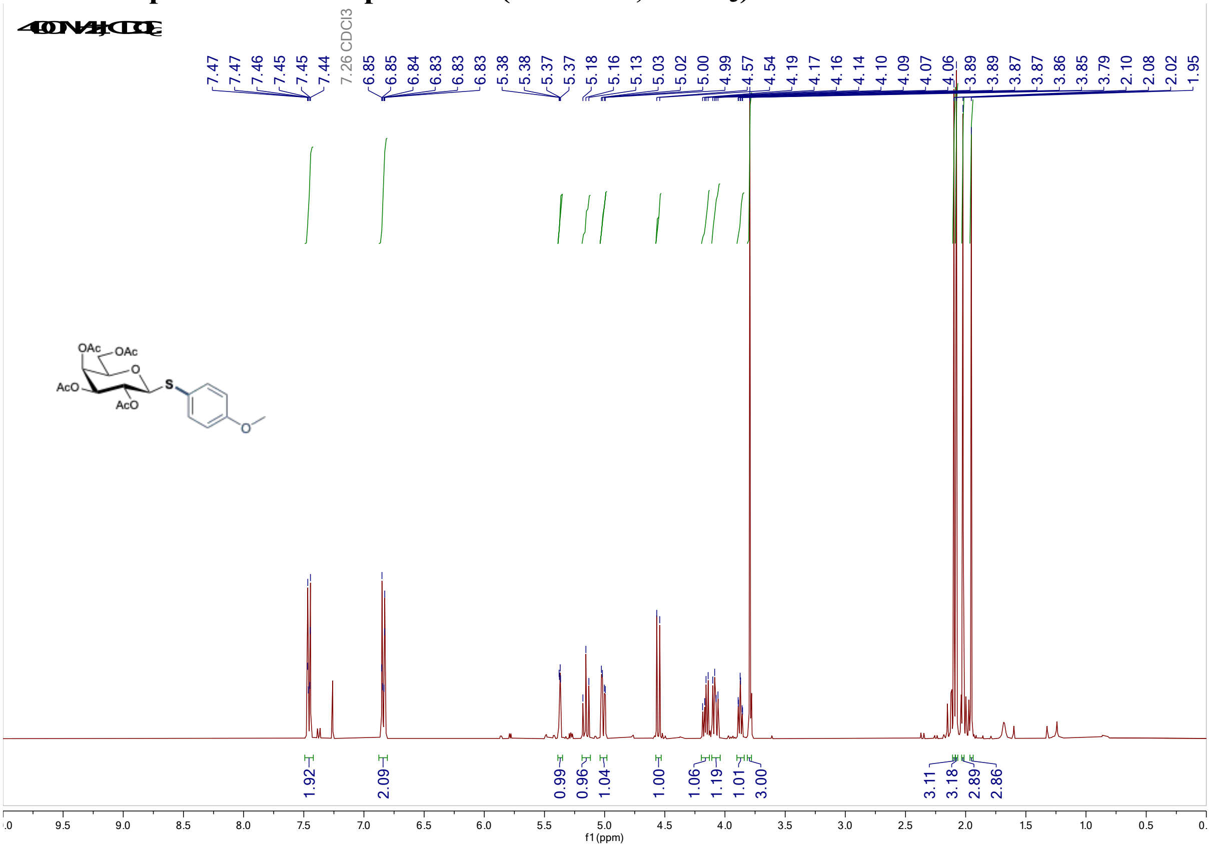

$^{13}C\{^{1}H\}$ NMR Spectrum of compound 35 (101 MHz, $CDCl_3$)

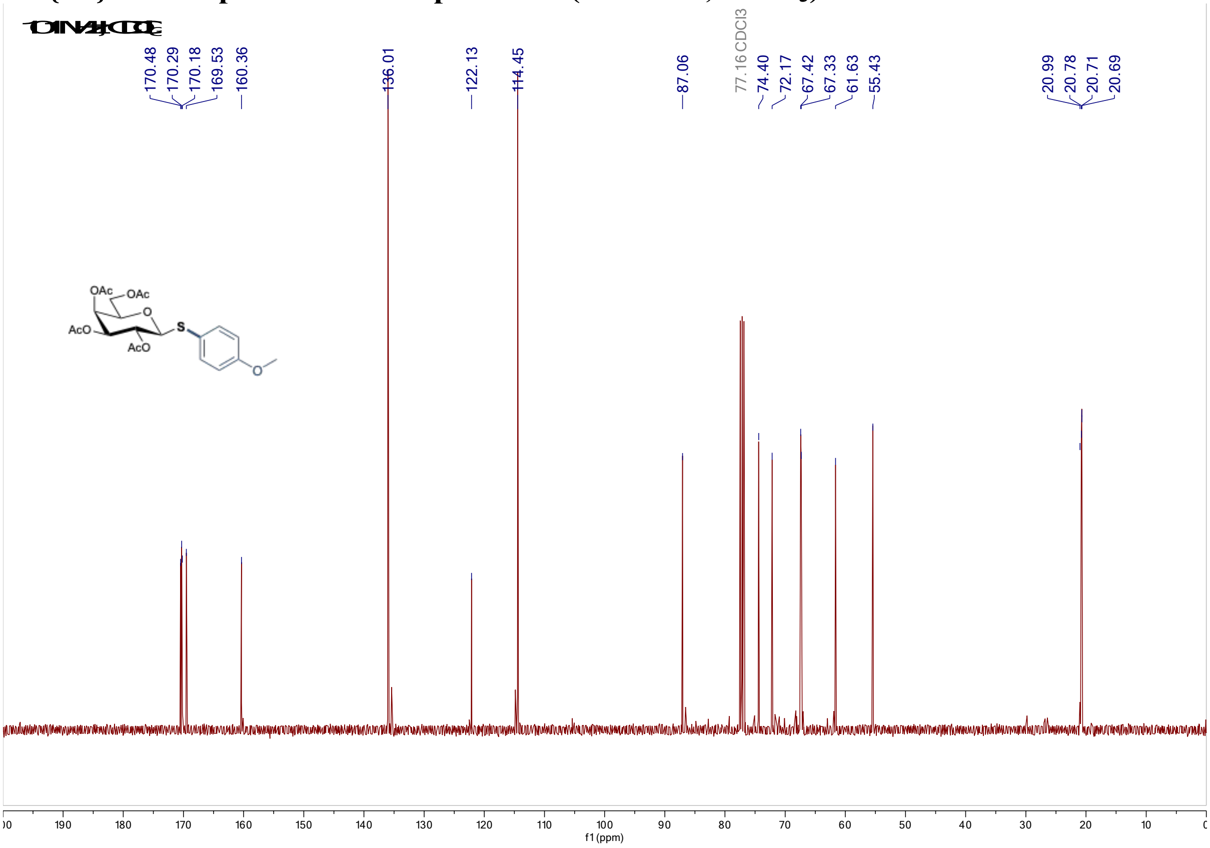

^{1}H NMR Spectrum of compound 36 (500 MHz, $CDCl_3$)

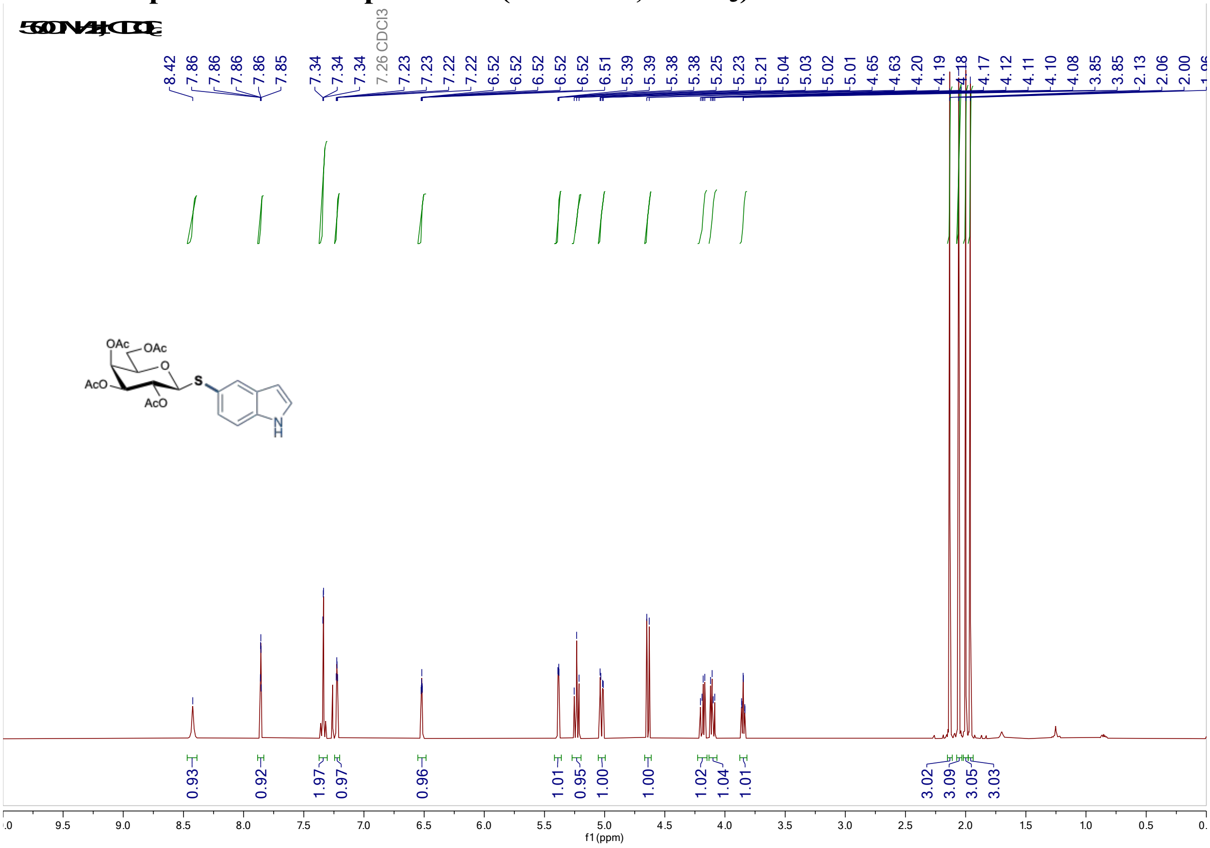

$^{13}C\{^1H\}$ NMR Spectrum of compound 36 (126 MHz, $CDCl_3$)

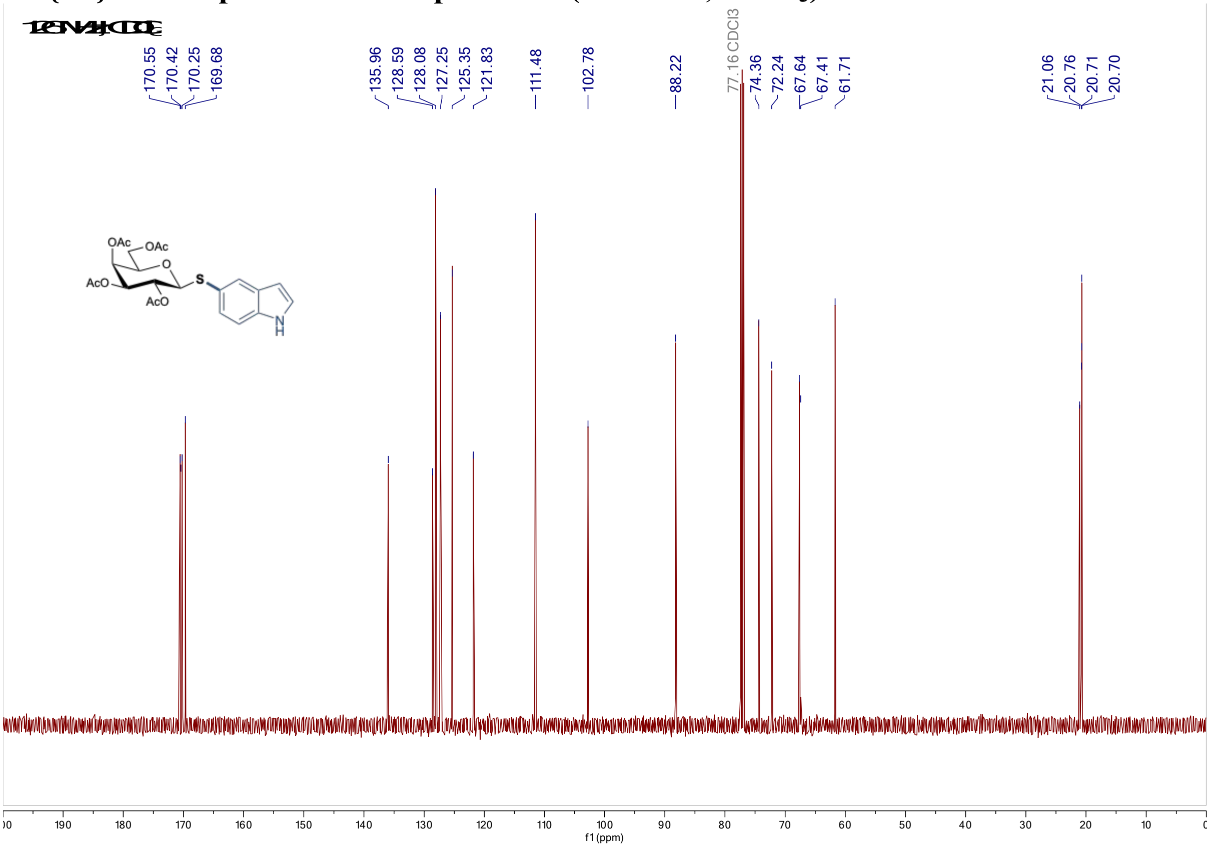

^{1}H NMR Spectrum of compound 37 (400 MHz, $CDCl_3$)

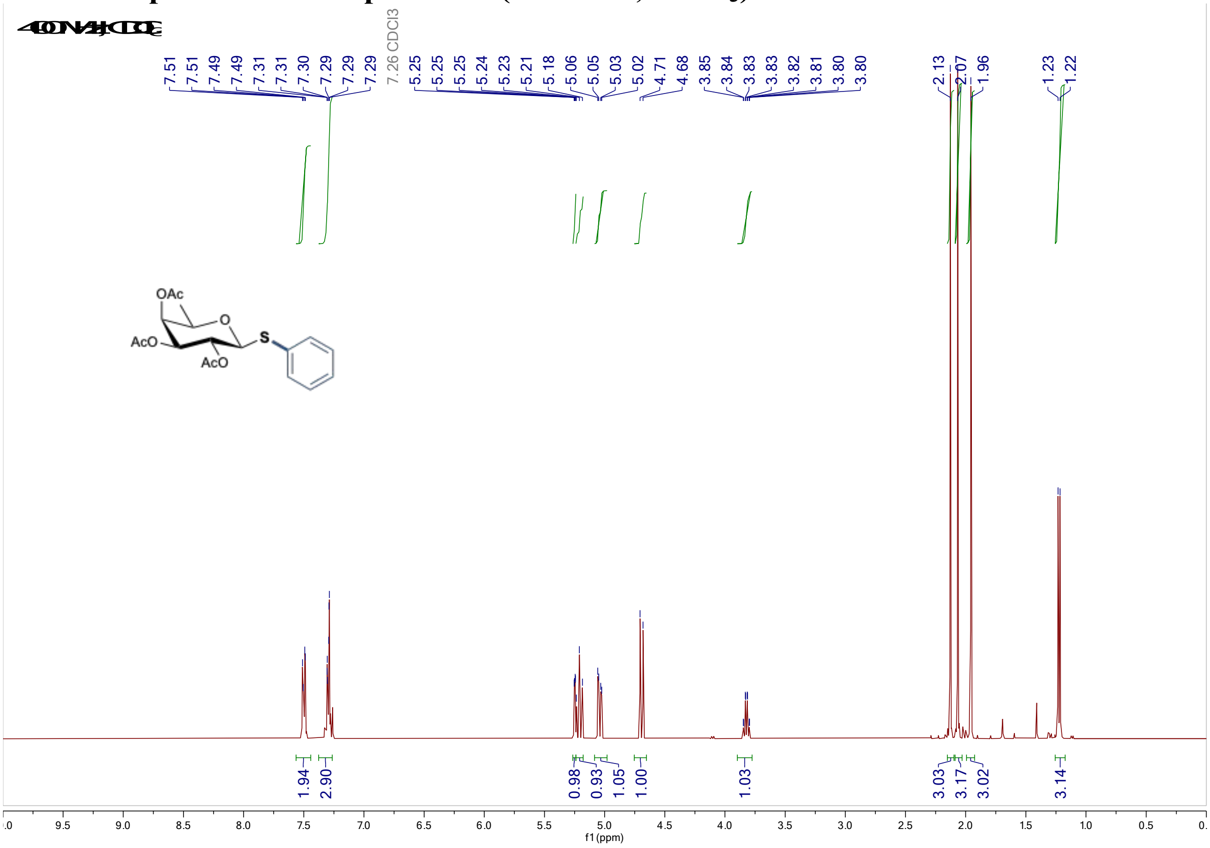

^{13}C{^{1}H} NMR Spectrum of compound 37 (101 MHz, $CDCl_3$)

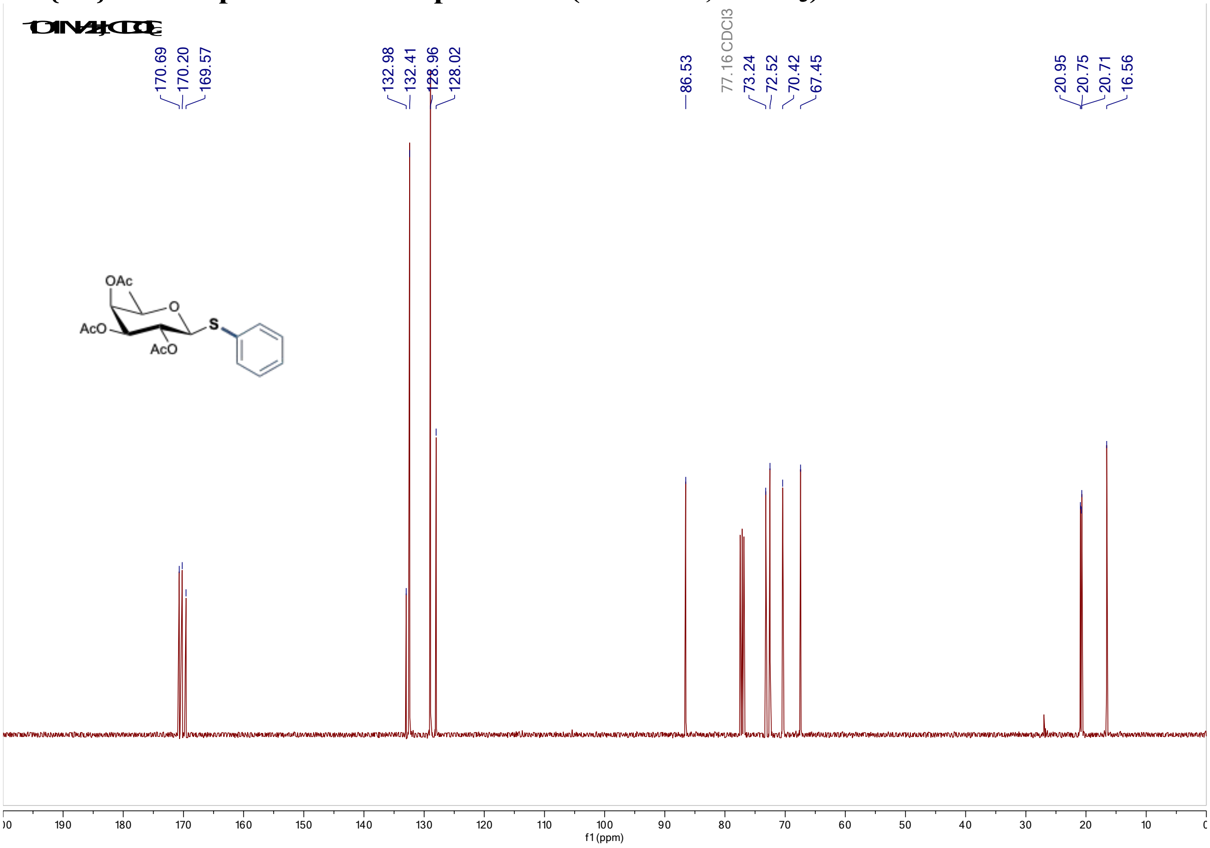

^{1}H NMR Spectrum of compound 38 (400 MHz, $CDCl_3$)

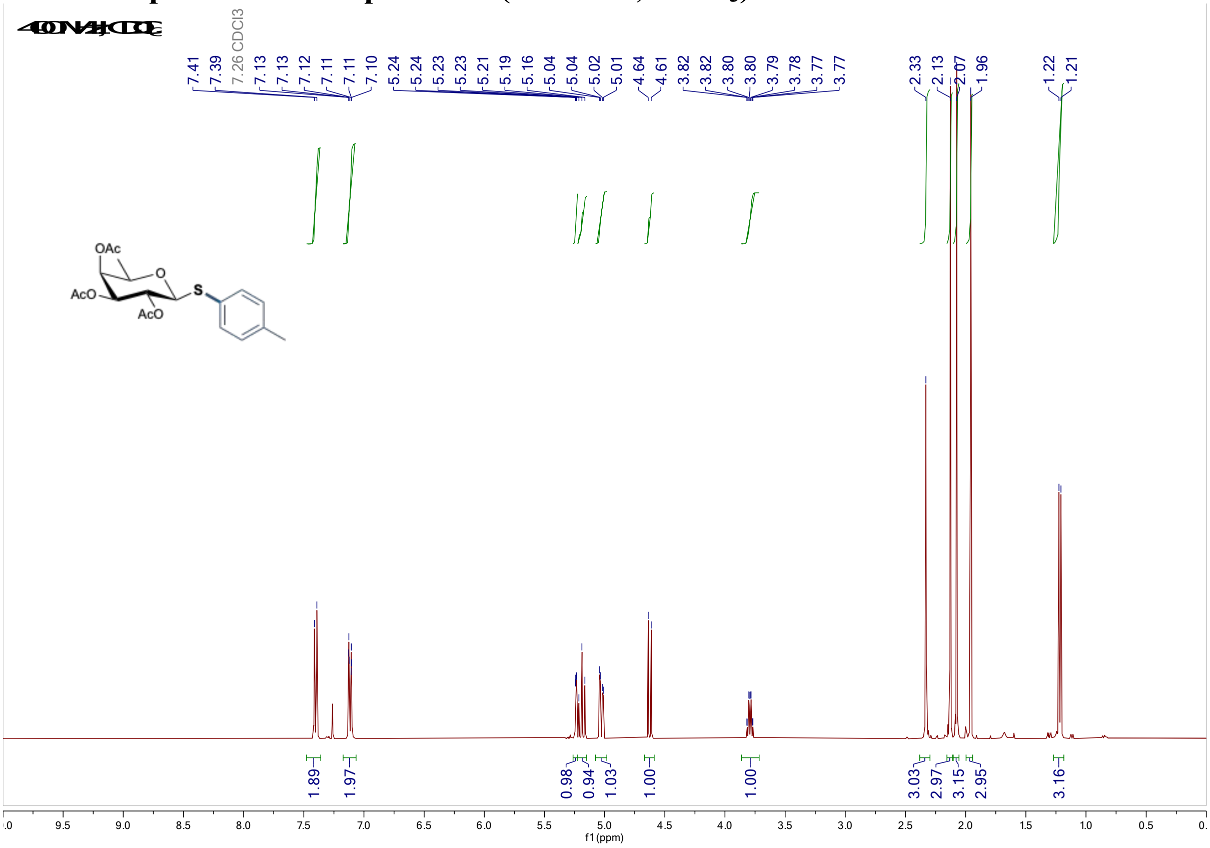

^{13}C{^{1}H} NMR Spectrum of compound 38 (101 MHz, $CDCl_3$)

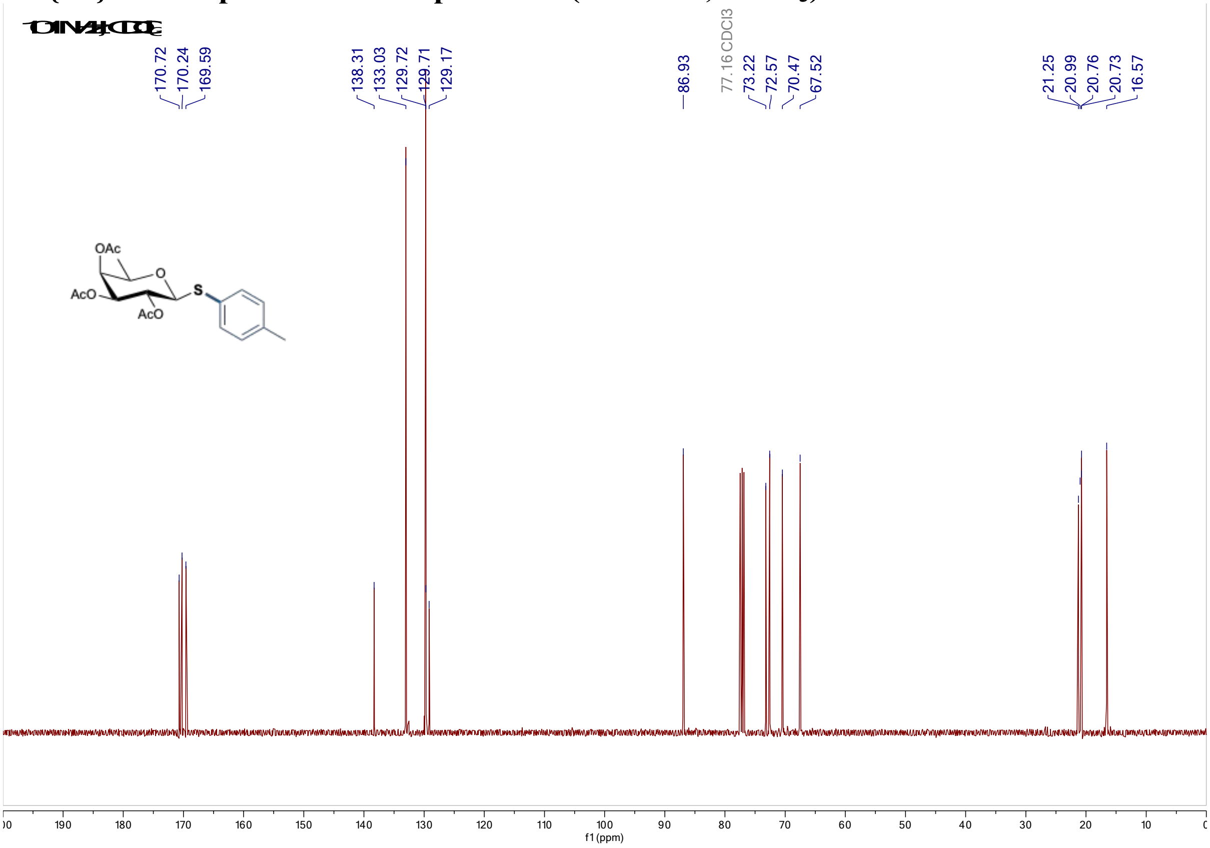

^{1}H NMR Spectrum of compound 39 (400 MHz, $CDCl_3$)

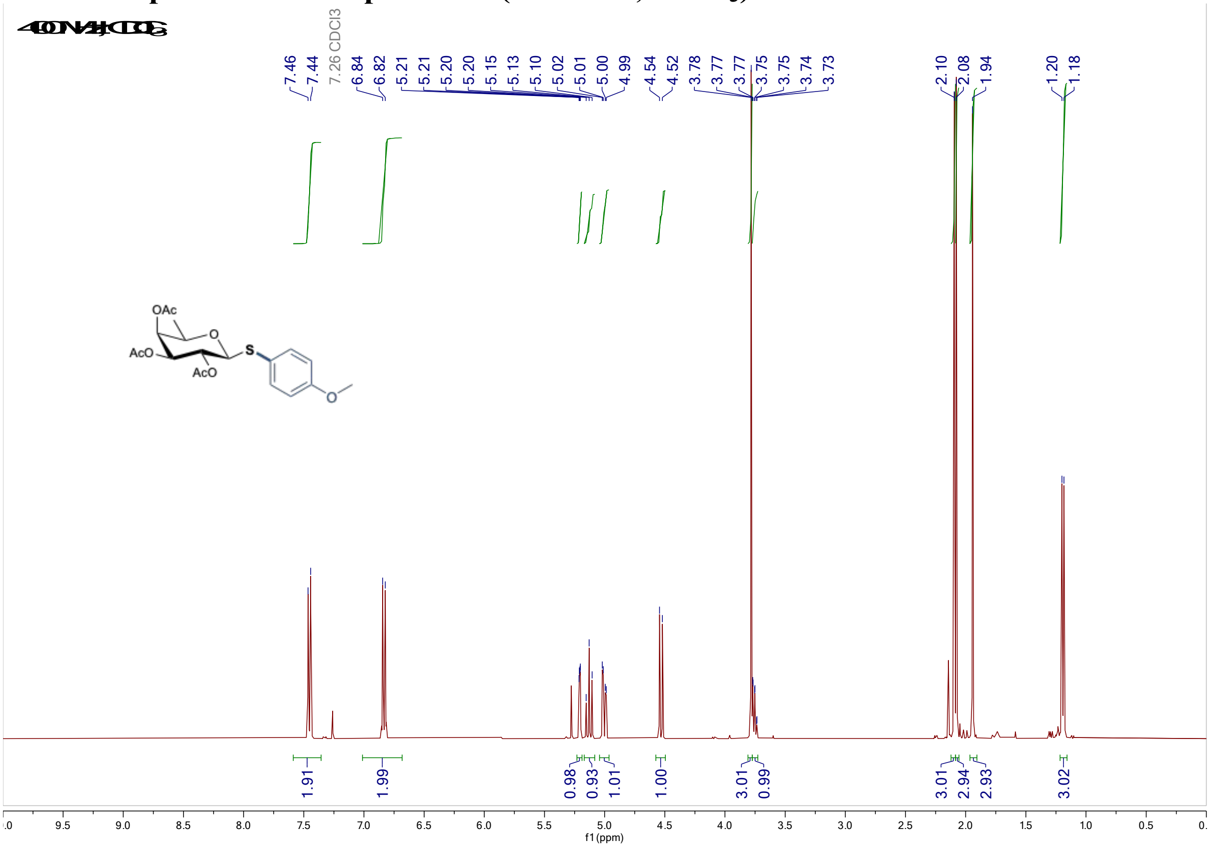

$^{13}C\{^{1}H\}$ NMR Spectrum of compound 39 (101 MHz, $CDCl_3$)

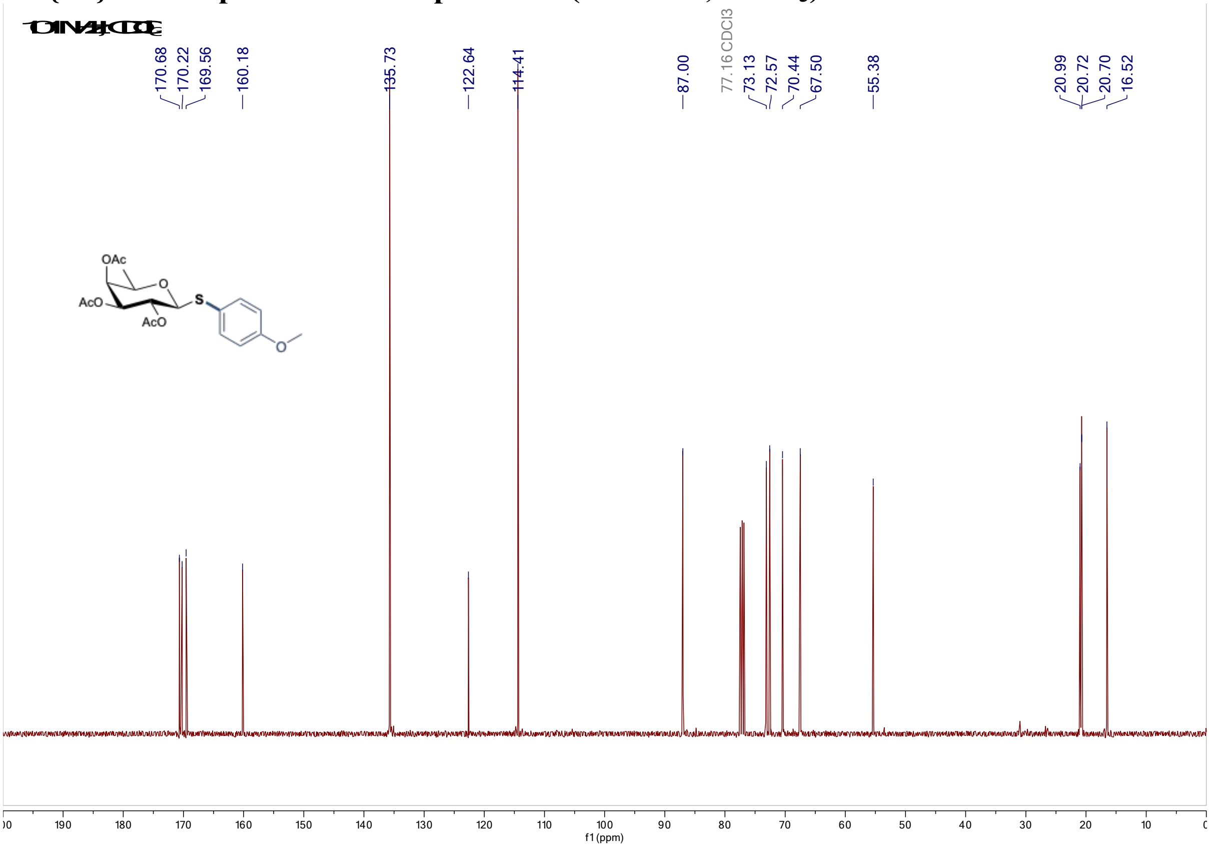

^{1}H NMR Spectrum of compound 40 (400 MHz, $CDCl_3$)

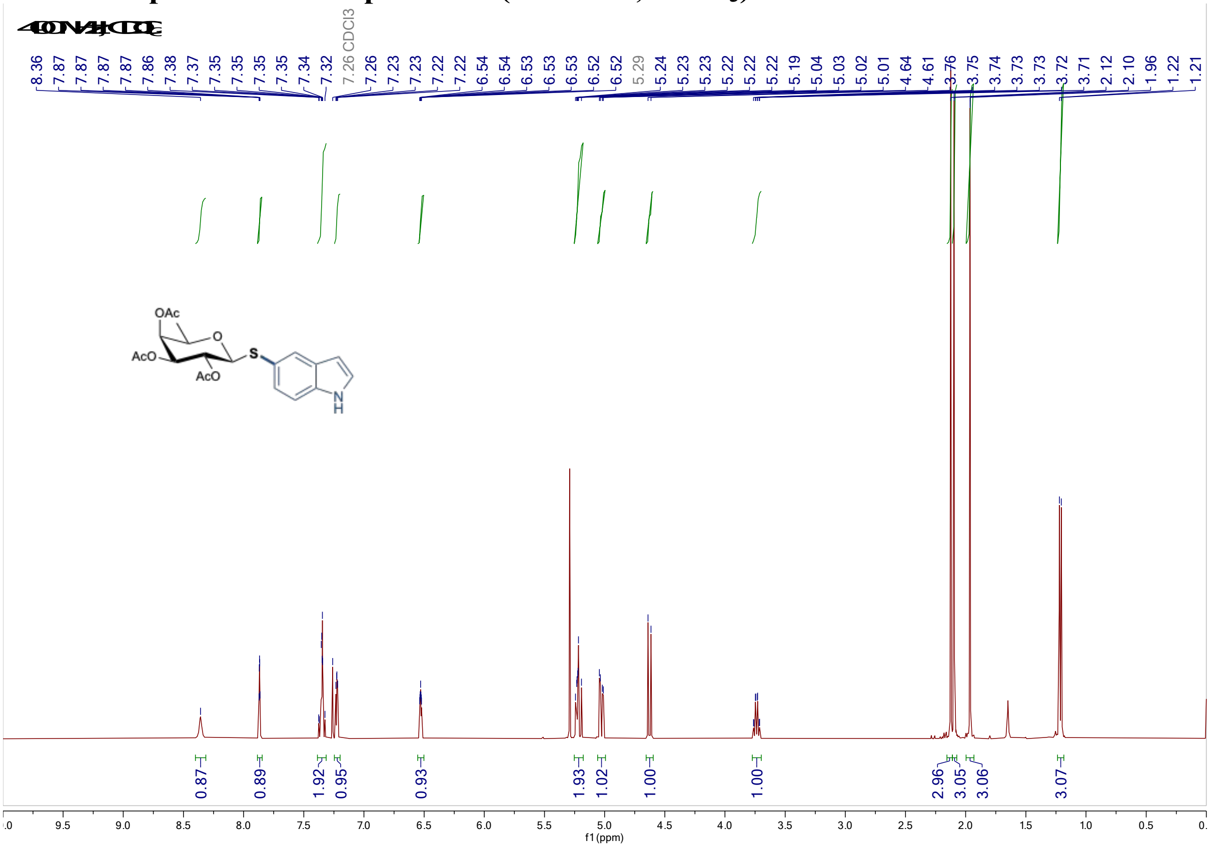

^{13}C{^{1}H} NMR Spectrum of compound 40 (101 MHz, $CDCl_3$)

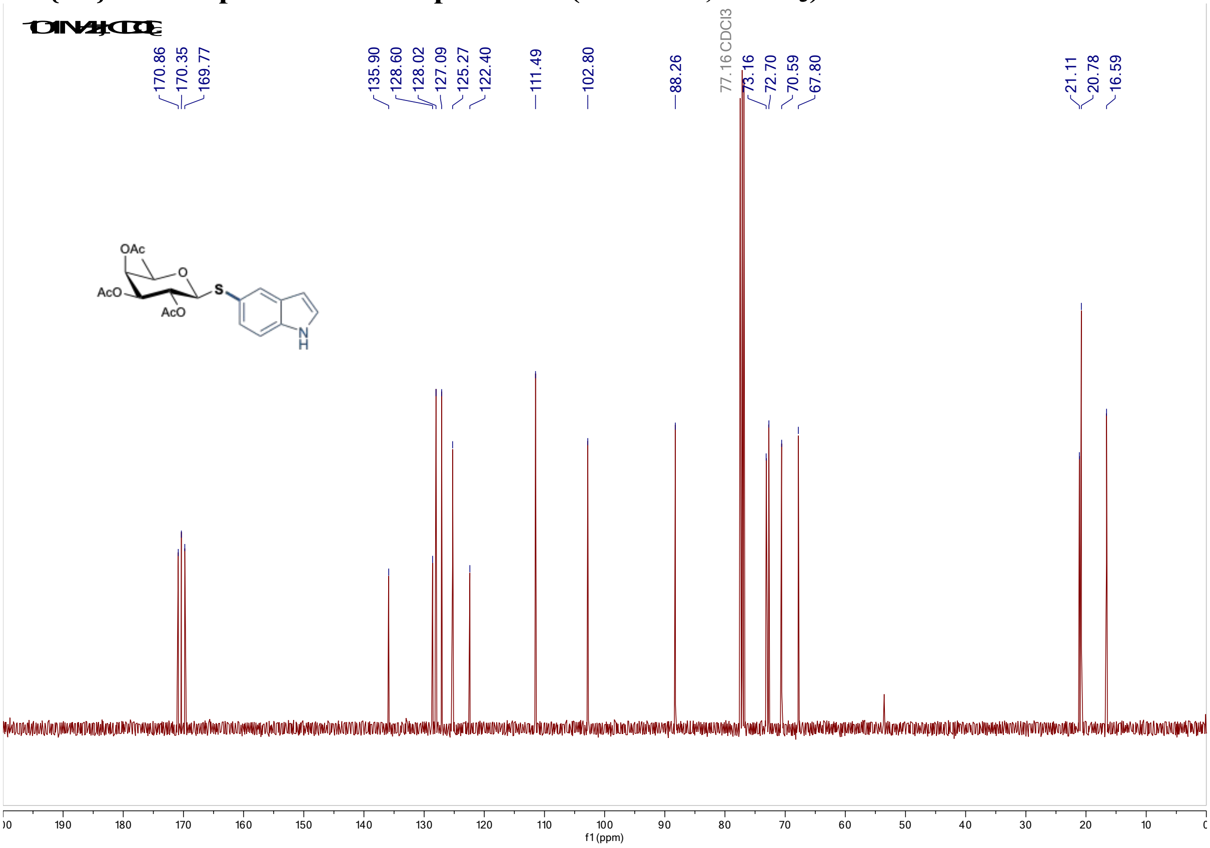

^{1}H NMR Spectrum of compound 41 (400 MHz, $CDCl_3$)

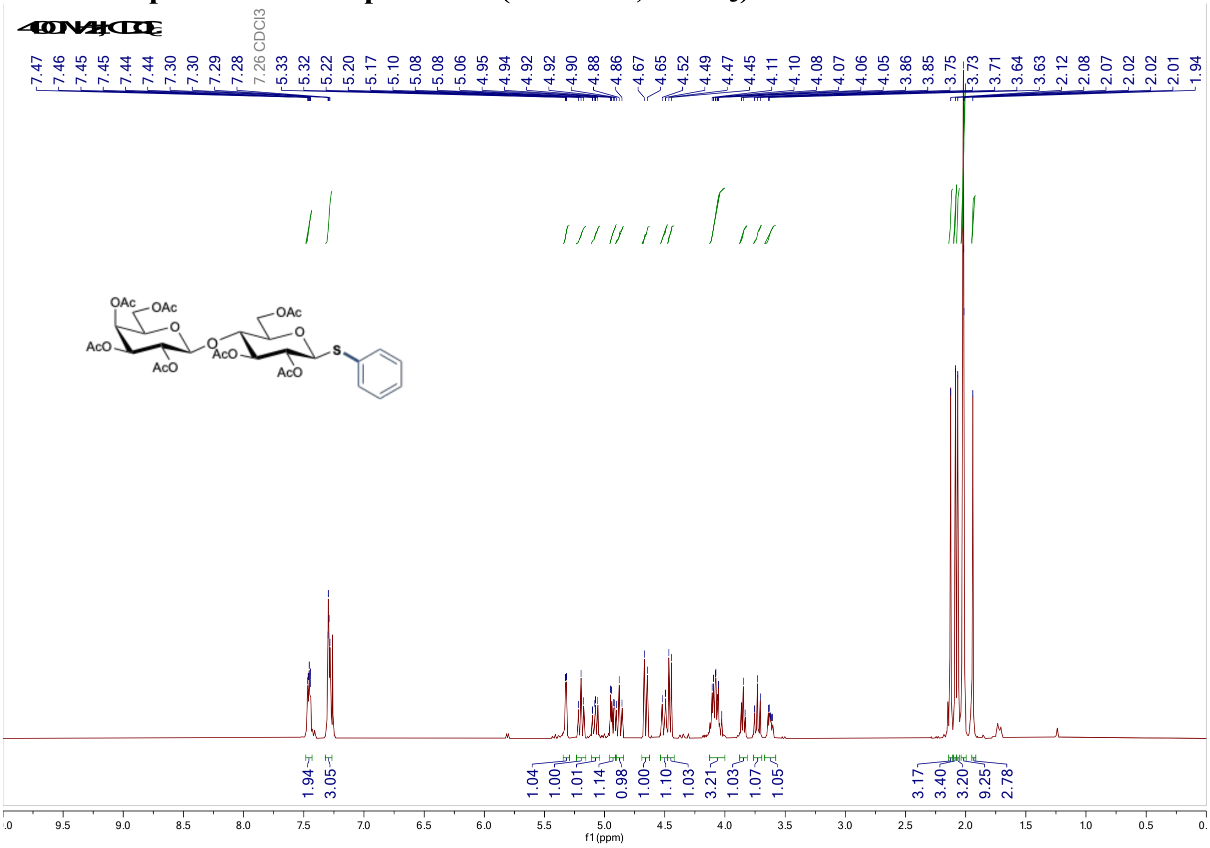

^{13}C{^{1}H} NMR Spectrum of compound 41 (101 MHz, $CDCl_3$)

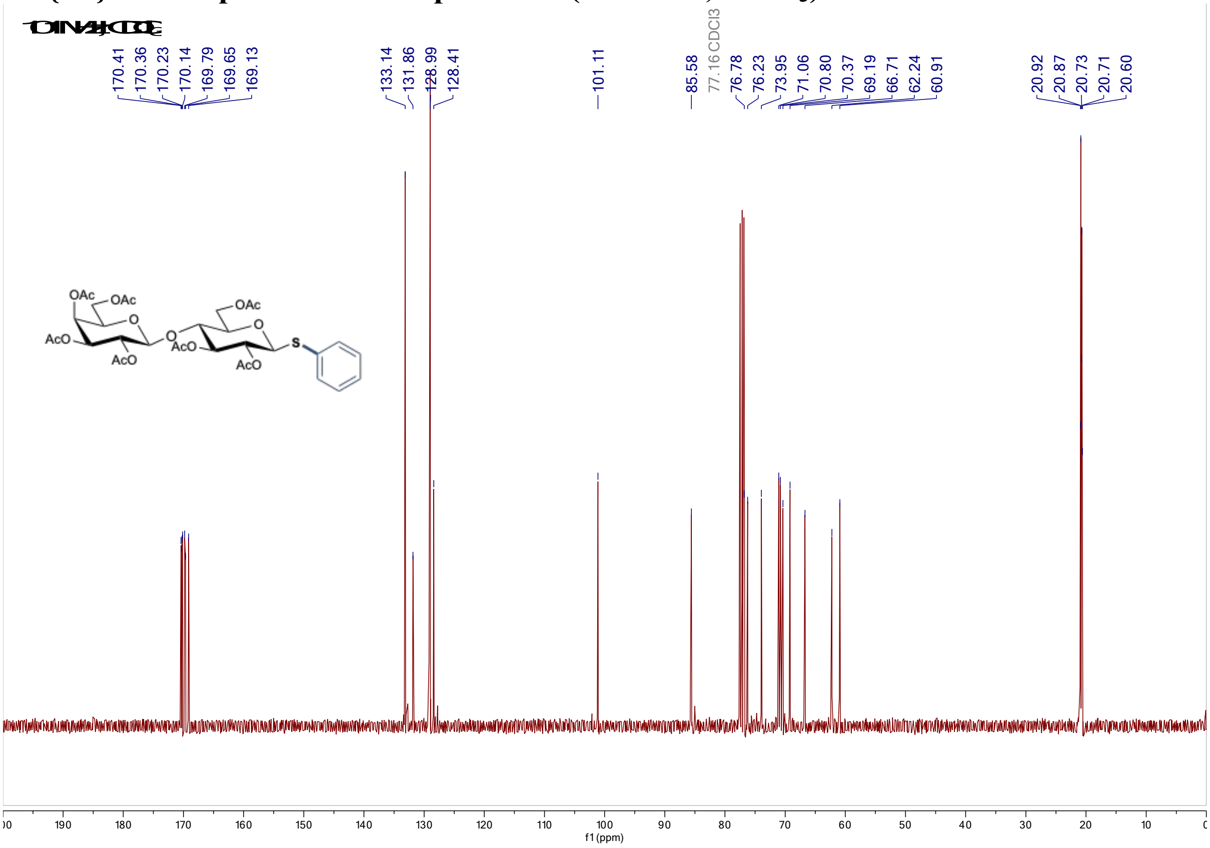

^{1}H NMR Spectrum of compound 42 (400 MHz, $CDCl_3$)

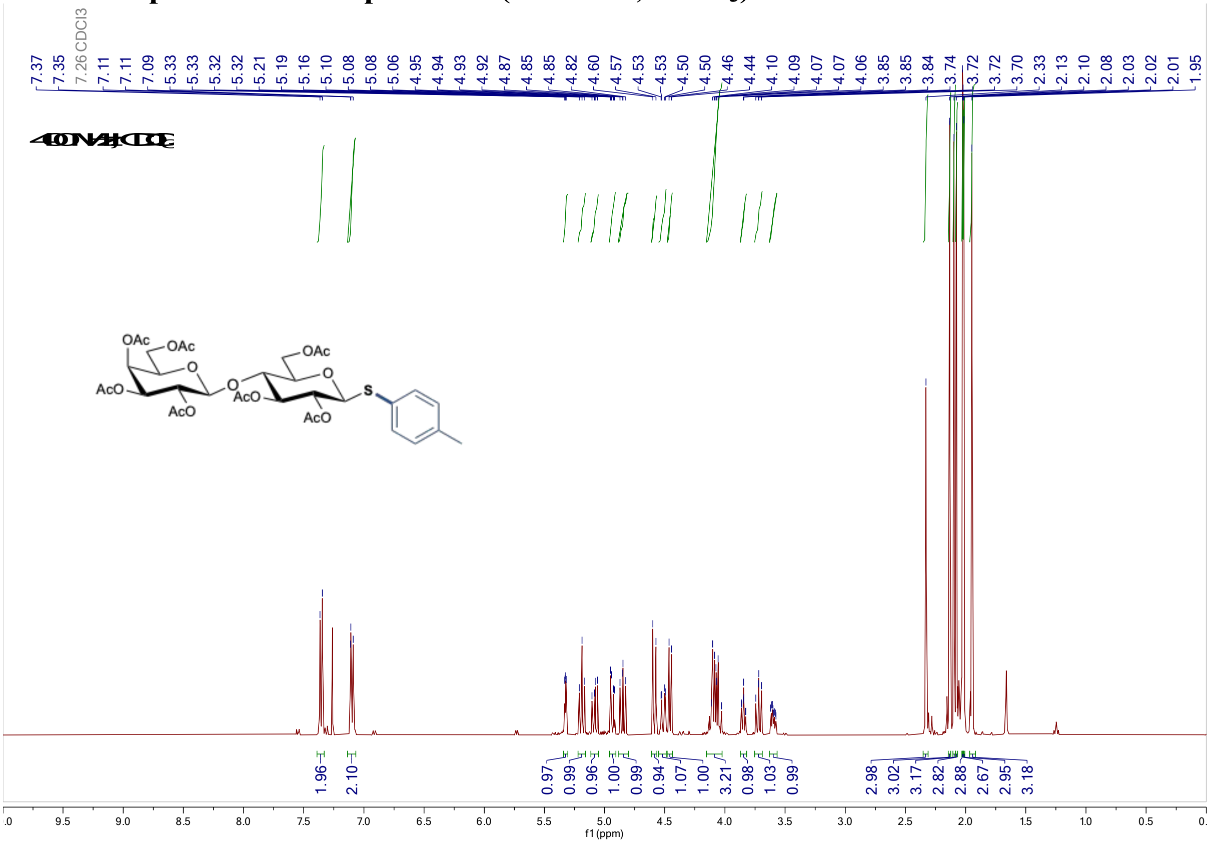

^{13}C{^{1}H} NMR Spectrum of compound 42 (101 MHz, $CDCl_3$)

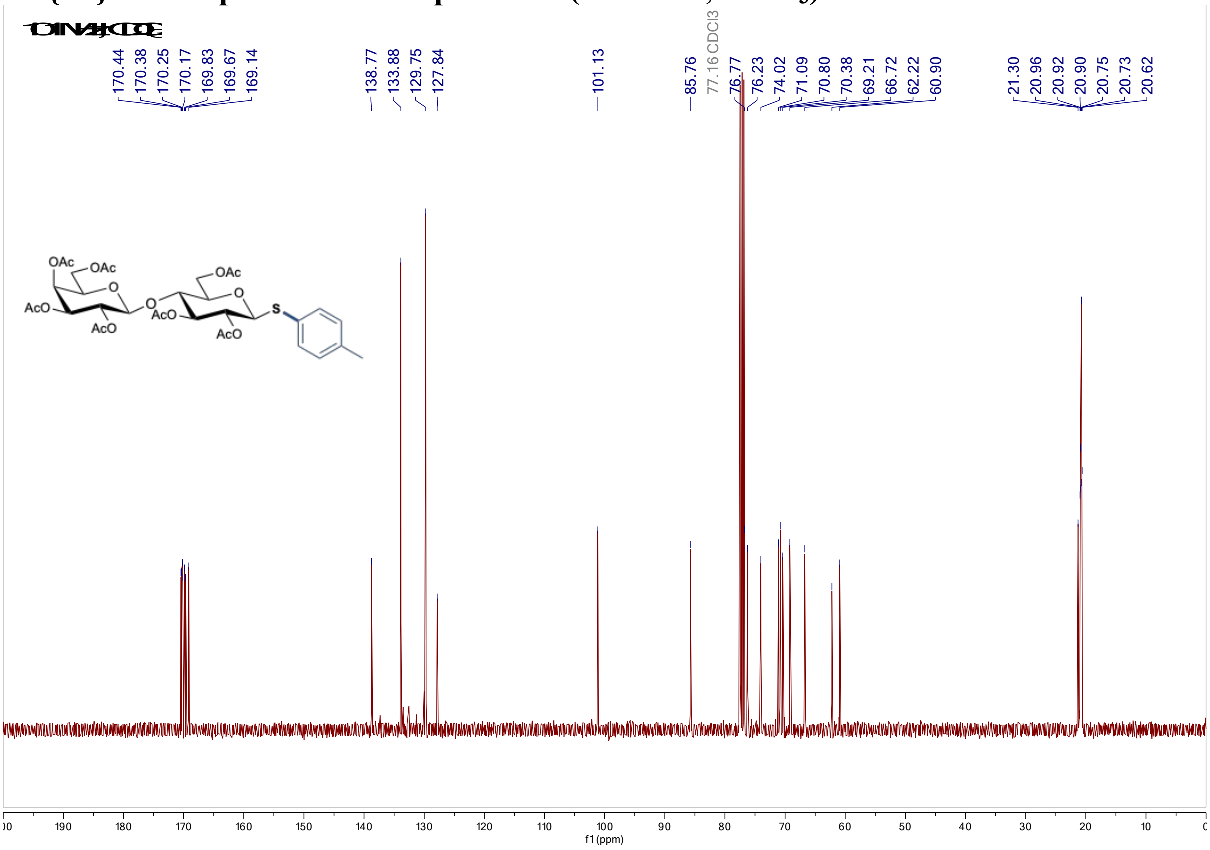

^{1}H NMR Spectrum of compound 43 (400 MHz, $CDCl_3$)

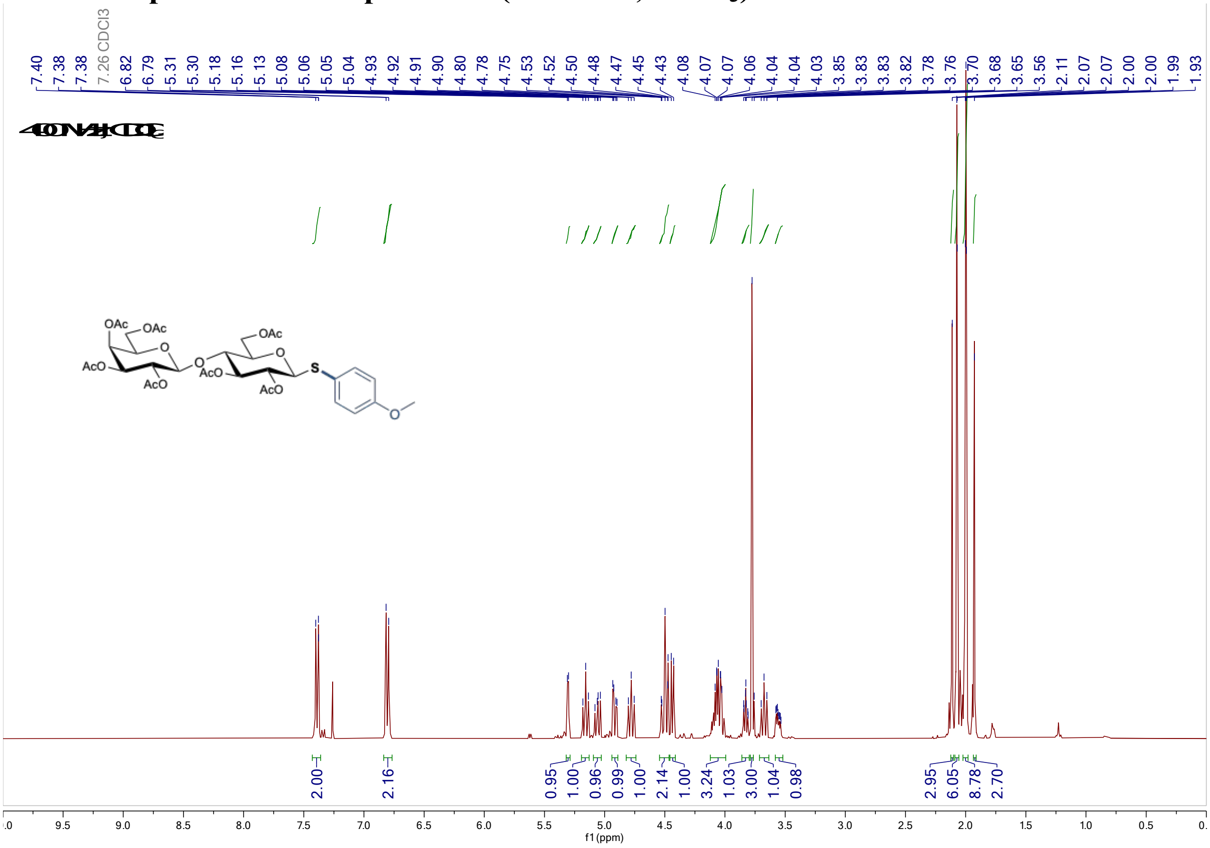

^{13}C{^{1}H} NMR Spectrum of compound 43 (101 MHz, $CDCl_3$)

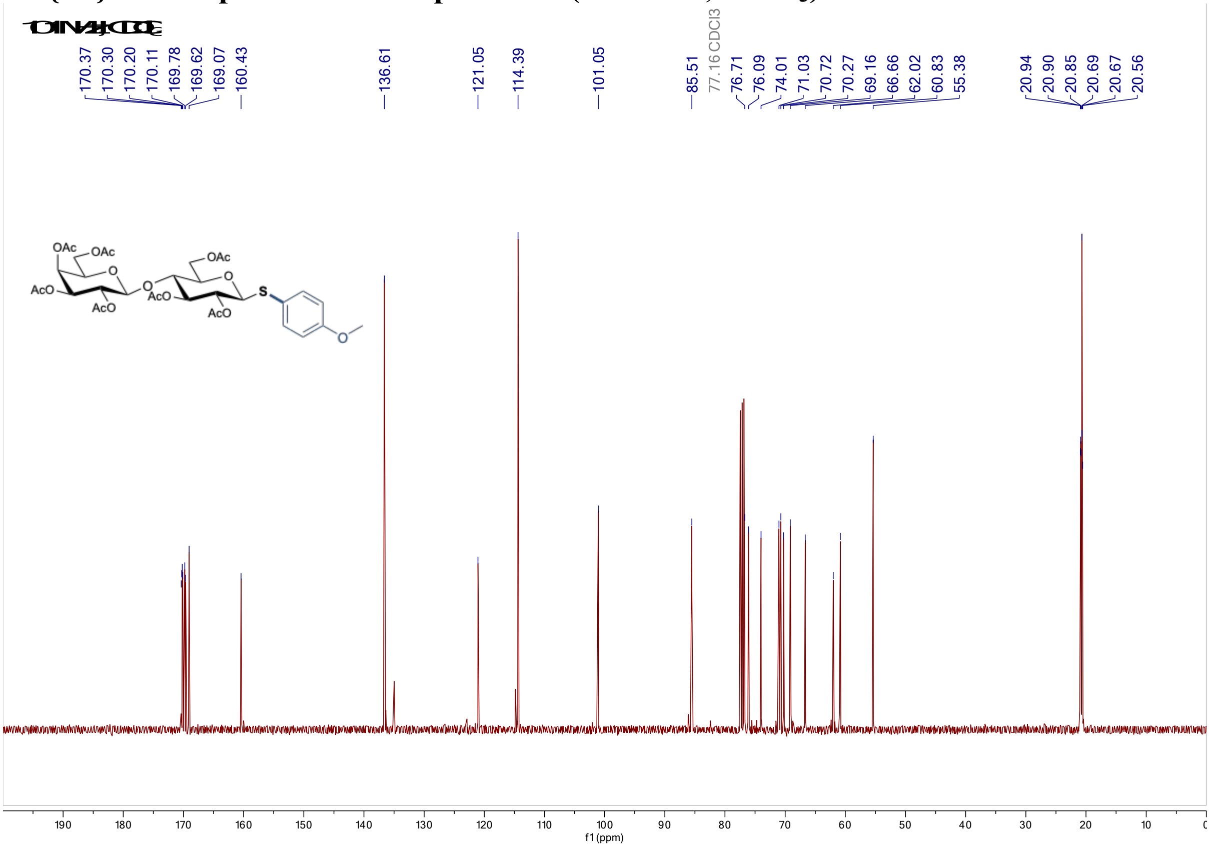

References

1. Yu, T. Y.; Pang, H. B.; Cao, Y. L.; Gallou, F.; Lipshutz, B. H., Safe, scalable, inexpensive, and mild nickel-catalyzed Migita-like C-S cross-couplings in recyclable water. *Angew. Chem. Int. Ed.* **2021,** *60*, 3708-3713.
2. HANSCH, C.; LEO, A.; TAFT, R., A Survey of Hammett Substituent Constants and Resonance and Field Parameters. *Chem. Rev.* **1991,** *91*, 165-195.
